# Efficacy and target engagement of dopamine agonist pramipexole for anhedonic depression: a randomized placebo-controlled trial

**DOI:** 10.1038/s41591-026-04465-9

**Published:** 2026-06-12

**Authors:** Filip Ventorp, Marie Asp, Sofia Olsson, Jesper Lindahl, Stefan Möller, Martina Svensson, Filip Ängeby, Armida Pravdinske, Johanna Tjernberg, Susann Schrey, Darya Ståhl, Viktor Magnusson, Emilia Eliasson, Anna Agelii-Weber, Erika Stålhammar, Danielle van Westen, Tomas Deierborg, Kristoffer N. T. Månsson, Diego A. Pizzagalli, Åsa Hammar, Åsa B. Tornberg, Johannes Björkstrand, Daniel Lindqvist

**Affiliations:** 1 Unit for Biological and Precision Psychiatry, Department of Clinical Sciences Lund, Lund University, Lund, Sweden; 2 Department of Psychiatry, Skåne University Hospital, Lund, Sweden; 3 Department of Psychiatry, Skåne University Hospital, Malmö, Sweden; 4 Department of Psychology, Lund University, Lund, Sweden; 5 Experimental Neuroinflammation Laboratory, Department of Experimental Medical Science, Lund University, Lund, Sweden; 6 Department of Clinical Chemistry and Pharmacology, Skåne University Hospital, Lund, Sweden; 7 Department of Clinical Sciences Lund, Lund University, Lund, Sweden; 8 Diagnostic Radiology, Department of Clinical Sciences Lund, Lund University, Lund, Sweden; 9 Image and Function, Skåne University Hospital, Lund, Sweden; 10 Center for Psychiatry Research, Department of Clinical Neuroscience, Karolinska Institutet, Stockholm, Sweden; 11 Department of Clinical Psychology and Psychotherapy, Babeș-Bolyai University, Cluj-Napoca, Romania; 12 Noel Drury, M.D. Institute for Translational Depression Discoveries, University of California, Irvine, Irvine, CA USA; 13 Department of Health Sciences, Lund University, Lund, Sweden

**Keywords:** Predictive markers, Depression, Combination drug therapy

## Abstract

Anhedonia is a core and disabling symptom of mood disorders with limited treatment options. We evaluated the efficacy and safety of the dopamine agonist pramipexole in patients with mood disorders characterized by clinically significant anhedonia. In this single-center, randomized, double-blind, placebo-controlled trial, adults with major depressive disorder, dysthymia or bipolar depression and elevated Snaith−Hamilton Pleasure Scale (SHAPS) scores were assigned (1:1) to flexible dose, once-daily oral pramipexole as add-on treatment or placebo for 9 weeks. The primary outcome was change in SHAPS score from baseline to week 9. Analyses were conducted in the modified intention-to-treat population. Eighty-five participants were randomized, and 82 were included in the analysis. The primary outcome was met: pramipexole was associated with a greater reduction in SHAPS scores compared to placebo (mean difference: −4.04, 95% confidence interval: −6.89 to −1.18, *P* = 0.006, Hedges’ *g* = 0.62). Exploratory analyses indicated that pramipexole was associated with increased light physical activity and relative preservation of reward-related ventral striatal activation. Improvements in anhedonia were sustained during a 6-month open-label extension. Pramipexole was generally well tolerated compared to placebo. Pramipexole significantly improved anhedonia and showed a favorable safety profile, supporting its potential as an augmentation strategy in mood disorders. ClinicalTrials.gov identifiers: NCT05355337 and NCT05825235.

## Abstract (web summary)

Pramipexole, in patients with major depressive disorder, dysthymia or bipolar depression, reduced Snaith−Hamilton Pleasure Scale scores significantly compared to placebo.

## Main

Mood disorders, including depressive and bipolar disorders, are leading causes of disability worldwide with enormous negative impact on society and the affected individuals^1^. Despite availability of multiple treatments, remission rates are low, and residual symptoms are common. Among these, anhedonia—the reduced ability to experience pleasure or motivation—is particularly disabling and is associated with poor treatment response, functional impairment and increased risk of suicide^2,3^. However, effective treatments specifically targeting anhedonia remain limited.

Currently, the selection of antidepressant treatments lacks objective measures, such as symptom profiles or biomarkers, to guide decision-making. Clinicians, therefore, often resort to trial-and-error methods, which can be time-consuming and less effective. The pursuit for precision in antidepressant treatments might be limited by a strict adherence to the categorical definitions of depressive disorders in diagnostic manuals when selecting participants for randomized controlled trials (RCTs). This approach may not fully capture the considerable clinical, and likely pathophysiological, heterogeneity of depression, leading to generic treatment recommendations rather than focusing on specific depression endophenotypes^4^. Anhedonia has emerged as a depression endophenotype of pivotal clinical relevance that generally lacks efficacious treatment^5,6^. Symptoms of anhedonia extend beyond major depressive disorder (MDD), with similar symptoms in bipolar depressive episodes and dysthymia. Clinical manifestations of anhedonia, including low motivation and reduced reward-seeking behavior, are assumed to be caused by dysregulation of reward processing within the brain. Central to this is the mesolimbic dopamine pathway, projecting from the ventral tegmental area to the ventral striatum^7^. Dopamine agonism has been proposed as a potential treatment target for a depression subtype marked by severe anhedonia^8,9^. The dopamine receptor D3, with high expression levels in the nucleus accumbens of the ventral striatum, has emerged as a promising target for such therapies. Pramipexole, a dopamine agonist with high affinity for the D3 receptor, is commonly used in Parkinson’s disease and alleviates not only motor symptoms but also anhedonia in patients with Parkinson’s disease^10^. Long-term administration of pramipexole leads to desensitization of dopamine autoreceptors and an increase in dopaminergic neurotransmission^11^, potentially normalizing a reduced dopaminergic tone, which may be a mechanism of anhedonic depression^9^. Pramipexole has shown robust antidepressant effects in individuals with treatment-resistant depression^12^, and clinical observations indicate that it ameliorates anhedonia in this patient group, with greater benefit observed at higher doses^8^. A pilot study conducted by our group provided preliminary evidence suggesting an antianhedonic effect of pramipexole^9^, which was partly supported by a recent RCT^12^.

In the present transdiagnostic RCT, we investigated the efficacy of high-dose pramipexole on anhedonia and related symptoms in mood disorders. Patients with MDD, dysthymia or bipolar depression, all with significant anhedonia at baseline, were randomized to receive pramipexole or placebo, added to ongoing antidepressant or mood-stabilizing medication, for 9 weeks. After the randomized controlled phase, patients could enter a 6-month open-label extension. Primary outcome was change in the total score on the Snaith−Hamilton Pleasure Scale (SHAPS)^13^. Secondary clinical outcomes included both expert-rating and self-rating scales, which assessed a wide range of depression-related symptoms. Cognitive performance, reward responsiveness and inflammatory and dopaminergic biomarkers were assessed before and after treatment in a subset of participants. Accelerometers were used to provide objective measures of treatment-associated changes in physical activity. We also aimed to demonstrate target engagement of pramipexole on reward-related neurocircuitry in the ventral striatum using functional magnetic resonance imaging (fMRI) in conjunction with the Monetary Incentive Delay (MID) task before and after treatment. Together, these multimodal assessments were designed to provide a comprehensive evaluation of pramipexole’s clinical efficacy and mechanistic effects on anhedonia.

## Results

### Patient disposition

**Fig. 1:**
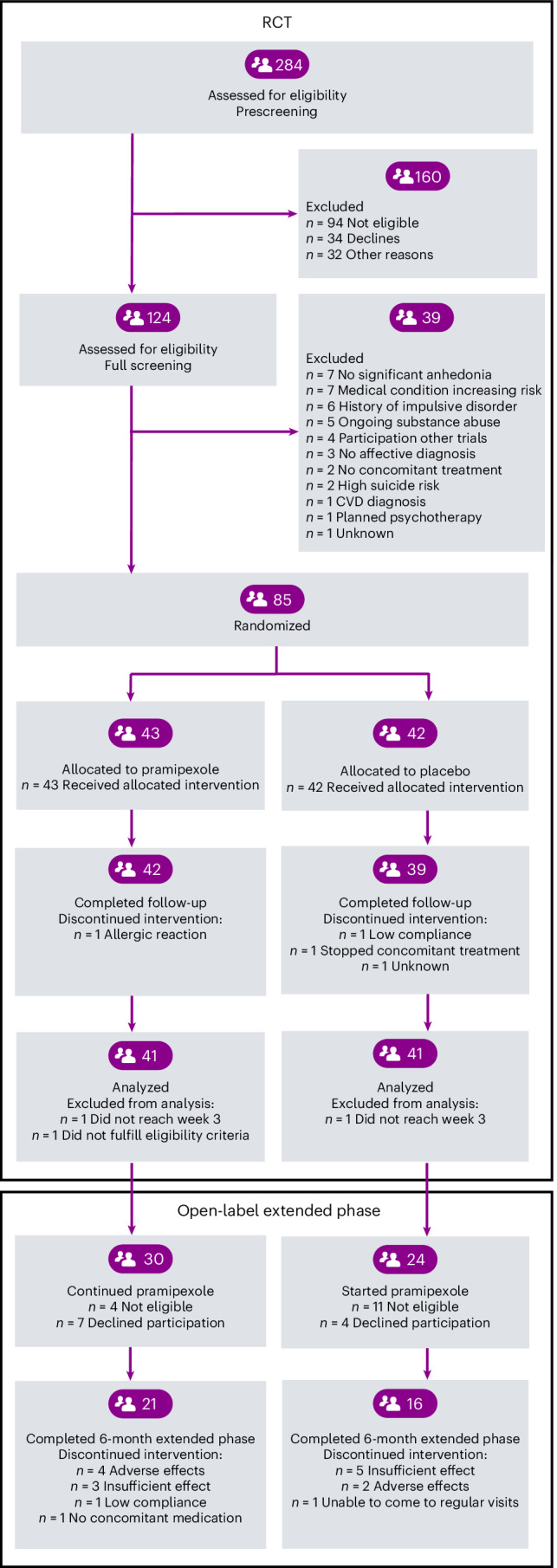
Participant flow through the trial. A CONSORT flow diagram illustrating the progression of participants through each stage of the RCT and in the open-label extended phase. CVD, cardiovascular disease.

The flow of study participants is summarized in the CONSORT diagram in Fig. 1. A total of 284 patients underwent screening, and 85 fulfilled eligibility criteria and were randomized to either pramipexole (*n* = 43) or placebo (*n* = 42). Patient enrollment in the RCT began on 16 February 2023 and was completed on 4 April 2025. All patients had a main diagnosis of MDD, dysthymia or bipolar disorder with a current depressive episode and significant anhedonia defined as 3 or 4 points on three or more items on the SHAPS^13^. In this 9-week RCT, participants had either flexible dose pramipexole or identical placebo added to their ongoing, stable treatment of antidepressant or mood-stabilizing medication.

Two participants did not reach week 3 and, therefore, had no post-baseline symptom data and were consequently excluded from the analysis. One of these participants (65-year-old male, MDD diagnosis, placebo group) discontinued due to adverse events and poor compliance, and one of these participants (50-year-old male, MDD diagnosis, pramipexole group) discontinued due to an allergic reaction that was later assessed as unrelated to pramipexole. One participant (39-year-old male, MDD diagnosis, pramipexole group) met the eligibility criteria at screening but not at baseline. Despite this, this individual was randomized but later confirmed by the external monitor to be ineligible for the study and was, therefore, excluded from the analysis.

Another two participants had at least one post-baseline assessment but dropped out before week 9 (end of study). Both were included in the modified intention-to-treat (ITT) analyses. One of these patients (placebo) dropped out after week 3 due to personal reasons, and the other patient (placebo) was withdrawn from the study after week 6 because he abruptly discontinued his regular antidepressant medication without informing the study team, which deviated from the protocol.

Demographic and clinical characteristics of the modified ITT sample are summarized in Table 1. Mean (s.d.) age was 47.22 (13.66) years. Thirty-seven (45.12%) participants were female. Most study participants were diagnosed with MDD, comprising 63.41% of the pramipexole group and 56.10% of the placebo group, for an overall prevalence of 59.76%. Dysthymia was the primary diagnosis for 34.15% of participants receiving pramipexole and for 41.46% of participants in the placebo group, totaling 37.80% across the sample. A small proportion (2.44%) in each group was diagnosed with bipolar disorder with a current depressive episode. Serotonin−norepinephrine reuptake inhibitors (SNRIs) were used by 19.51% of the patients in the pramipexole group and by 41.46% of the patients in the placebo group.

**Table 1:** Demographics and clinical characteristics for participants with at least one post-baseline assessment (*n* = 82)

	Pramipexole (*n* = 41)	Placebo (*n* = 41)	Overall (*n* = 82)
Demographics			
Age, mean (s.d.)	47.44 (13.14)	47.00 (14.32)	47.22 (13.66)
Percent women (self-reported)	46.34% (*n* = 19)	43.90% (*n* = 18)	45.12% (*n* = 37)
BMI, mean (s.d.)	27.63 (4.95)	27.88 (6.26)	27.76 (5.61)
Regular nicotine user (%)^a^	29.27% (*n* = 12)	29.27% (*n* = 12)	29.27% (*n* = 24)
Clinical characteristics			
Primary diagnosis			
Percent MDD	63.41% (*n* = 26)	56.10% (*n* = 23)	59.76% (*n* = 49)
Percent bipolar disorder with depressive episode	2.44% (*n* = 1)	2.44% (*n* = 1)	2.44% (*n* = 2)
Percent dysthymia	34.15% (*n* = 14)	41.46% (*n* = 17)	37.80% (*n* = 31)
Comorbid conditions			
Percent anxiety disorder	39.02% (*n* = 16)	39.02% (*n* = 16)	39.02% (*n* = 32)
History			
Percent previous ECT	9.76% (*n* = 4)	12.20% (*n* = 5)	10.98% (*n* = 9)
Percent previous suicide attempt(s)	9.76% (*n* = 4)	9.76% (*n* = 4)	9.76% (*n* = 8)
Total number of depressive episodes (s.d.)	5.07 (3.90)	4.83 (3.54)	4.95 (3.70)
Prior pharmacological treatments (s.d.)	3.98 (2.88)	3.51 (2.65)	3.74 (2.76)
Concomitant antidepressant treatments (%)			
SSRI	46.34% (*n* = 19)	34.15% (*n* = 14)	40.24% (*n* = 33)
SNRI	19.51% (*n* = 8)	41.46% (*n* = 17)	30.49% (*n* = 25)
Other serotonergic^b^	31.71% (*n* = 13)	24.39% (*n* = 10)	28.05% (*n* = 23)
NDRI	24.39% (*n* = 10)	24.39% (*n* = 10)	24.39% (*n* = 20)
TCA	7.32% (*n* = 3)	2.44% (*n* = 1)	4.88% (*n* = 4)
Mood stabilizer	12.20% (*n* = 5)	12.20% (*n* = 5)	12.20% (*n* = 10)
Psychotherapy	4.88% (*n* = 2)	2.44% (*n* = 1)	3.66% (*n* = 3)
Mean baseline total scores on rating scales			
SHAPS (s.d.)	41.54 (5.57)	39.51 (5.16)	40.52 (5.43)
HDRS-6 (s.d.)	7.37 (2.47)	7.20 (2.15)	7.28 (2.30)
MADRS-S (s.d.)	27.63 (6.43)	25.73 (6.99)	26.68 (6.74)
DARS (s.d.)	23.10 (10.34)	25.44 (12.10)	24.27 (11.25)
AES-S (s.d.)	50.71 (5.96)	48.66 (8.45)	49.68 (7.34)
GAD-7 (s.d.)	9.98 (4.81)	8.98 (5.13)	9.48 (4.97)
ISI (s.d.)	12.71 (5.44)	13.41 (6.12)	13.06 (5.76)
BBQ (s.d.)	16.59 (13.19)	19.07 (14.52)	17.83 (13.84)
Dosage			
Mean dose at endpoint, mg salt (s.d.)^c^	3.53 (1.13)	4.21 (0.51)	3.86 (0.94)

Data are expressed as mean (s.d.) or as percentages. NDRI, norepinephrine−dopamine reuptake inhibitor; SSRI, selective serotonin reuptake inhibitor; TCA, tricyclic antidepressants.

^a^Includes Swedish snus (moist tobacco) and e-cigarettes (vapes)

^b^Mirtazapine, vortioxetine and agomelatine

^c^Only participants who reached the endpoint and were included in ITT analyses are included (*n* = 80).

After completion of the randomized controlled phase, patients were evaluated for inclusion in an open-label extension study with pramipexole for up to an additional 6 months. This assessment was conducted by a non-blinded study physician who had not participated in the RCT phase. Of the 80 patients who completed the RCT, 65 were eligible for the extension. Among these, 54 chose to enroll, and 37 completed the full 6-month open-label treatment period.

Compliance was evaluated using a journal in which participants recorded each day that pramipexole or placebo was taken according to the dose titration schedule. Pill counts were also carried out systematically at each scheduled visit to assess compliance. A patient had 100% compliance if they had taken the study intervention each day in the correct dose. Mean compliance was above 96.47% at each treatment visit, across the randomized controlled phase and the open-label phase.

### Primary outcome

**Fig. 2:**
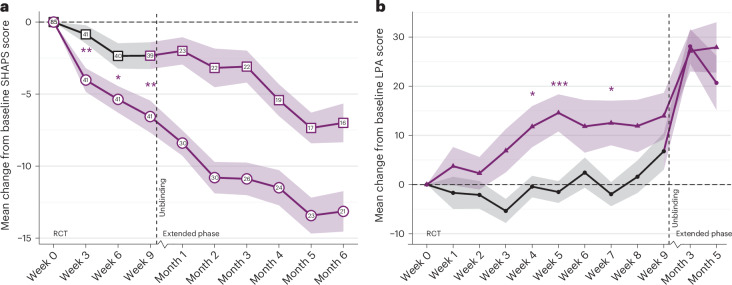
Effects of pramipexole on anhedonia and LPA over time. **a**,**b**, Longitudinal changes from baseline in SHAPS scores (**a**) and LPA levels (**b**). Mean changes from baseline in SHAPS total scores and LPA (mean minutes per day) are shown over the 9-week RCT period and the 6-month open-label extension phase for participants receiving either pramipexole (purple line) or placebo (gray line). In the ΔSHAPS line chart, each data point is annotated with the number of participants (*n*) at the corresponding timepoint. Valid LPA data were available for *n* = 29 (placebo) and *n* = 32 (pramipexole) participants per treatment arm during the RCT. Of these, *n* = 12 (former placebo) and *n* = 17 (former pramipexole) provided valid accelerometer data at month 3 and *n* = 11 (former placebo) and *n* = 15 (former pramipexole) at month 5 of the extension phase. After unblinding, the *x* axis timescale changes from weeks to months; month labels indicate time since unblinding, not time since RCT initiation. Group means are represented by solid lines, with shaded ribbons indicating ±1 s.e. Asterisks indicate RCT timepoints at which pairwise comparisons from the mixed model revealed statistically significant differences. Pairwise comparisons between treatment groups at each timepoint were performed using EMMs derived from the linear MMRM. All tests were two-sided, and no adjustments were made for multiple comparisons. For **a**, the *P* values were 0.005 at week 3, 0.03 at week 6 and 0.006 at week 9. For **b**, the *P* values were 0.03 at week 4, 0.0008 at week 5 and 0.01 at week 7. *P < 0.05, **P < 0.01, ***P < 0.001.

At baseline, SHAPS scores were similar between groups (pramipexole: mean = 41.54, s.d. = 5.57; placebo: mean = 39.51, s.d. = 5.16). Over the course of the trial, SHAPS scores decreased more in the pramipexole group than in the placebo group (Fig. 2a). By endpoint (week 9), mean scores were 34.98 (s.d. = 8.04) in the pramipexole group and 37.26 (s.d. = 7.43) in the placebo group. In the linear mixed-model repeated measures (MMRM) analysis, there was a significant main effect of treatment (*P* = 0.0022), indicating that pramipexole produced greater overall improvement in SHAPS scores compared to placebo across weeks 3–9.

Pairwise comparisons of the estimated marginal mean (EMM) showed significant differences favoring pramipexole at all three timepoints (Fig. 2a). At the 9-week primary endpoint, the estimated mean decrease in SHAPS score from baseline in the pramipexole group was 6.47 points compared to 2.44 points in the placebo group. The difference in mean change was −4.04 (95% confidence interval: −6.89 to −1.18, *P* = 0.006, Hedges’ *g* = 0.62). As shown in Fig. 2a, SHAPS scores continued to improve during the extended open-label phase, when all participants received pramipexole, among both those previously randomized to pramipexole and those who had initially received placebo.

### Secondary outcomes

After 9 weeks of treatment, pramipexole led to significant improvements in anhedonia and apathy symptoms measured by the Dimensional Anhedonia Rating Scale (DARS) (*P* = 0.008, Hedges’ *g* = 0.59) and by the self-reported version of the Apathy Evaluation Scale (AES-S) (*P* < 0.001, Hedges’ *g* = 0.75), relative to placebo. At week 9, there were no significant differences between pramipexole and placebo on the self-rated Montgomery−Åsberg Depression Rating Scale (MADRS-S) (*P* = 0.13, Hedges’ *g* = 0.34) or the Hamilton Depression Rating Scale 6-item subscale (HDRS-6) (*P* = 0.11, Hedges’ *g* = 0.36). However, when analyzing data across all three post-baseline timepoints, pramipexole showed a significant main effect on both MADRS-S (*P* = 0.012) and HDRS-6 (*P* = 0.0024), indicating an overall benefit of pramipexole over placebo on depressive symptoms. Similarly, no significant treatment effects were observed at week 9 on the Generalized Anxiety Disorder 7-item scale (GAD-7) (*P* = 0.25, Hedges’ *g* = 0.26), the Insomnia Severity Index (ISI) (*P* = 0.89, Hedges’ *g* = 0.03) or the Brunnsviken Brief Quality of Life (BBQ) scale (*P* = 0.12, Hedges’ *g* = 0.35).

In the pramipexole group, 39.0% showed a ≥50% reduction in dichotomized SHAPS scores, and 22.0% achieved remission of anhedonia symptoms (<3 dichotomized points; Methods) at the end of the RCT. In the placebo group, the corresponding rates were 30.77% for response and 12.82% for remission (*χ*^2^ tests, all *P* ≥ 0.283). Among those who completed 6 months of open-label treatment (*n* = 37), 59.46% were responders and 37.84% achieved remission at month 6.

After 9 weeks of treatment, 21.95% of patients in the pramipexole group and 17.07% of patients in the placebo group were responders, and 14.63% in each group were remitters according to the MADRS-S (response: ≥50% improvement; remission: MADRS ≤ 10). No significant between-group differences were observed for remission or response (*χ*^2^ tests, *P* ≥ 0.655). Among those who completed 6 months of open-label treatment (*n* = 37), 51.35% were responders and 40.54% achieved remission at month 6. When participants who did not enter the extension phase due to lack of efficacy were classified as not in remission, and when those in the placebo group who had already achieved remission during the RCT and were, therefore, not eligible to enter the extension phase were classified as in remission, the adjusted remission rate was 39.22%.

Complete descriptive summaries for the RCT phase and the extended phase are provided in Extended Data Tables 1 and 2, respectively. Statistical summary of the RCT phase is provided in Extended Data Table 3.

### Safety

#### RCT phase

All randomized participants (*n* = 85) were included in the safety population (Supplementary Table 1). No serious adverse events occurred during the RCT phase. Pramipexole was generally well tolerated; one patient discontinued due to a suspected allergic reaction later assessed as unrelated to treatment. The overall incidence of any adverse event was high in both groups, but several specific events were more frequent with pramipexole, including sleep disturbances (72.09% versus 33.33%), nausea (60.47% versus 14.29%), dizziness (32.59% versus 4.76%), fatigue (46.51% versus 30.95%) and anxiety (30.23% versus 7.14%). Nightmares were more common in the placebo group (16.67% versus 2.33%).

During the RCT phase and the extended open-label phase, patients self-reported selected and modified items from the Young Ziegler Mania Rating Scale (M-YMRS), the Problem Gambling Severity Index (M-PGSI) and the Questionnaire for Impulsive−Compulsive Disorders in Parkinson´s Disease (M-QUIP) (Supplementary Table 2). If patients self-reported any such symptoms, they were further evaluated by the study physician for clinical significance.

Two pramipexole-treated patients with dysthymia developed mild manic symptoms, including decreased need for sleep, increased energy and flight of ideas. In one case, symptoms resolved after dose reduction; in the other, symptoms remained mild, treatment continued and symptoms resolved after discontinuation at study end. No between-group differences were observed on the M-PGSI, indicating no increased gambling risk. At week 3, more pramipexole-treated patients reported difficulties controlling buying (25.58% versus 4.76%), but this difference was not present at weeks 6 or 9. No impulse control symptoms led to withdrawal; management typically involved dose reduction or halted escalation.

#### Open-label extension phase

In the open-label extension phase (*n* = 54), in which all participants received pramipexole, the most reported adverse events were sleep disturbances (*n* = 27), fatigue (*n* = 13), anxiety (*n* = 10), nausea (*n* = 9) and loss of appetite (*n* = 9) (Supplementary Table 1).

Three patients reported acute daytime sleepiness; symptoms resolved after dose reduction in one case and led to treatment discontinuation in another. Three serious adverse events occurred, two of which were in the same patient: one patient was hospitalized for myocardial infarction and later dyspnea related to anticoagulant therapy. One patient was hospitalized with dizziness and a suspected cerebellar cerebrovascular event. No pathology was detected on computed tomography angiography or MRI, and the patient fully recovered. None of these serious adverse events was considered related to pramipexole, and both patients completed the study.

Self-ratings on M-YMRS, M-PGSI and M-QUIP during the open-label phase (Supplementary Table 2) were followed-up clinically when positive. In no case were impulse control symptoms judged sufficiently severe to warrant discontinuation.

### Exploratory outcomes

#### Light physical activity

**Extended Data Fig. 1:**
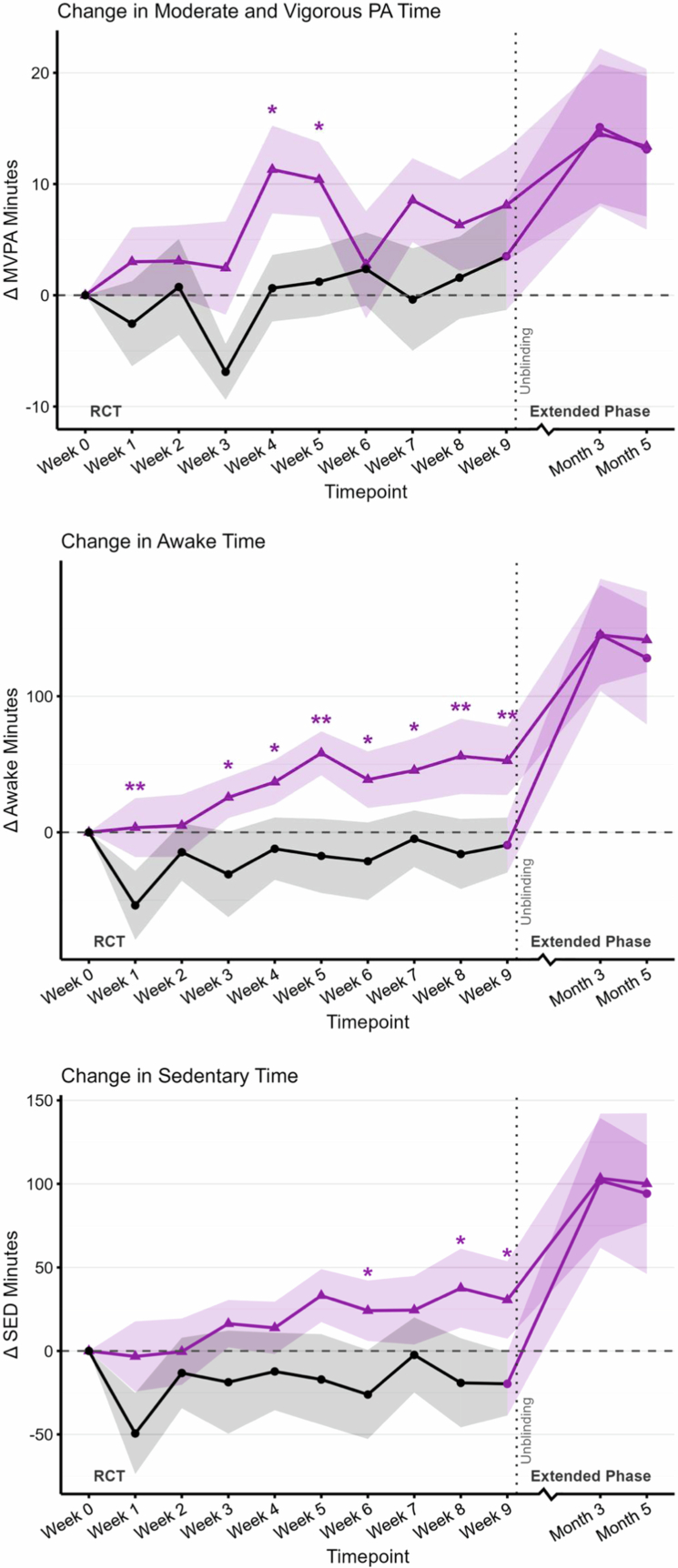
Treatment-associated change in awake time, sedentary time (SED), and moderate-to-vigorous physical activity (MVPA). Extended Data Table 1. Change from baseline in awake time, sedentary time (SED), and moderate-to-vigorous physical activity (MVPA), expressed as mean minutes/day. Valid data were available for n = 29 (placebo) and n = 32 (pramipexole) participants across all RCT time points. Of these, 29 (n = 12 former placebo; n = 17 former pramipexole) contributed data during the open-label extension. After unblinding, the x axis time scale changes from weeks to months; month labels indicate time since unblinding, not time since RCT initiation. Lines show group means with shaded ribbons representing ±1 SE. The dashed horizontal line indicates zero change. Purple denotes pramipexole and black placebo. Asterisks indicate significant between-group differences from mixed models of RCT data (p < 0.05, p < 0.01). Pramipexole increased awake time relative to placebo, with a significant main effect of treatment (p = 0.0096) and between-group differences at Weeks 1 and 3–9 (all p < 0.05). The largest effect occurred at Week 5 (Hedges’ g = 0.80, p = 0.001), consistent with a large treatment effect. Among participants initially assigned to placebo, awake time increased from Week 9 to Month 5 during the extension phase (Hedges’ g = 0.85, p = 0.023). No significant main effect of treatment was observed for SED (p = 0.098), indicating comparable overall trajectories between groups during the RCT. SED also did not change significantly from Week 9 to Month 5 in participants switching from placebo to pramipexole (Hedges’ g = 0.64, p = 0.069). However, exploratory pairwise comparisons revealed between-group differences at Weeks 6 (Hedges’ g = 0.54, p = 0.030), 8 (Hedges’ g = 0.58, p = 0.048), and 9 (Hedges’ g = 0.56, p = 0.024). For MVPA, neither the main effect of treatment (p = 0.13) nor the time-by-treatment interaction (p = 0.42) reached statistical significance during the RCT. However, pairwise contrasts revealed significant between-group differences at Week 4 (Hedges’ g = 0.59, p = 0.041) and Week 5 (Hedges’ g = 0.62, p = 0.014), suggesting modest mid-treatment increases in MVPA. Comparisons between Week 9 and Month 5 during the extension phase showed no significant change in MVPA among participants originally assigned to placebo (Hedges’ g = 0.56, p = 0.11). Taken together with the results for light physical activity (LPA; Fig. 2), these findings indicate that pramipexole increased both awake time and LPA but did not consistently reduce SED or increase MVPA. The increase in LPA without a corresponding reduction in SED may be explained by the overall extension of awake time.

Light physical activity (LPA), expressed as average minutes per day, was quantified using accelerometry, providing an objective measure of engagement in daily physical activities at different intensities^14^. Change scores of LPA are depicted in Fig. 2b. Additional accelerometry outcomes, including awake time, sedentary time (SED) and moderate-to-vigorous physical activity (MVPA), are shown in Extended Data Fig. 1, and information on wear time and valid periods is provided in Supplementary Data Fig. 1.

Consistent with our hypotheses, there was a significant main effect of treatment (*P* = 0.0031) in the MMRM analysis, which included all 9 weeks of the RCT, indicating that pramipexole produced greater overall improvement in LPA delta scores compared to placebo. A model excluding early-phase data (weeks 1 and 2) revealed an even stronger treatment effect (*P* = 0.0004). Pairwise comparisons of the EMMs revealed significant advantage for pramipexole at week 4 (*P* = 0.027), week 5 (*P* = 0.0008) and week 7 (*P* = 0.011), with Hedges’ *g* values ranging from 0.57 to 0.87. For the group that received placebo during the RCT and pramipexole in the extension, LPA significantly increased at month 5 compared to week 9 in the RCT (Hedges’ *g* = 0.72, *P* = 0.045). In mixed-effects analyses across all timepoints of the RCT and the extended phase, separating within-person and between-person effects, higher LPA was significantly associated with lower SHAPS scores (*P* < 0.001).

#### Reward-related blood oxygen level dependent activity response in the ventral striatum

Reward-related brain activity was assessed before and after treatment using 7-Tesla fMRI and a simplified version of the MID task. All participants were invited to take part in the fMRI substudy. Forty-eight participants (pramipexole: *n* = 25; placebo: *n* = 23) were included in the fMRI analysis. Several exclusions were made for reasons such as refusal to participate; medical contraindications such as implants or surgical history incompatible with the imaging center’s safety protocols; and technical difficulties encountered during the scanning. The participants included in the fMRI analyses were similar between the groups in terms of mean age and sex distribution. Specifically, the mean age was 43.88 years in the pramipexole group and 47.91 years in the placebo group, with women accounting for 40.00% and 43.48% of each group, respectively.

**Fig. 3:**
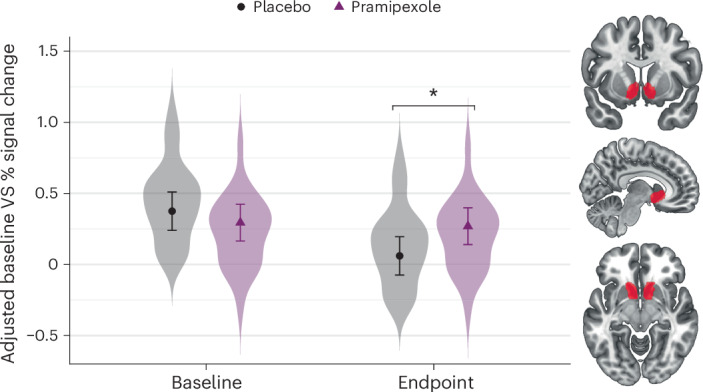
Effects of pramipexole on reward‑related ventral striatal BOLD activation. Violin plots show the distribution of model‑predicted BOLD signal percent signal change in the bilateral ventral striatum (VS), reflecting the contrast between high-reward and low-reward conditions during the MID task phase, for participants receiving pramipexole (*n* = 25) or placebo (*n* = 23). Circles (placebo) and triangles (pramipexole) represent EMMs obtained from the MMRM that included baseline BOLD percent signal change as a covariate. Error bars indicate 95% confidence intervals. Pairwise comparisons confirmed the hypothesis that VS activation was greater from before treatment to after treatment in the pramipexole group than in the placebo group (*P* = 0.030). The statistical test was two-sided, and no adjustments were made for multiple comparisons. To the right, representative anatomical brain images illustrate the bilateral VS ROIs used in the analysis. Asterisks indicate timepoints where pairwise comparisons from mixed model revealed statistically significant differences (*P* < 0.05).

To estimate reward-related brain activity, we extracted the mean blood oxygen level dependent (BOLD) signal from a bilateral ventral striatum region of interest (ROI) (Fig. 3) during the anticipation phase for high-reward versus low-reward trials for all participants before and after treatment. Subsequently, we estimated marginal means for each group and timepoint using a mixed model, and these adjusted means were used for group comparisons. For this contrast, we observed a significant group difference (*P* = 0.030, Hedges’ *g* = 0.64), indicating a preserved reward-related activity in the pramipexole group compared to decreased ventral striatum activity in the placebo group (Fig. 3). Within the pramipexole group, we observed a trend toward a significant negative correlation between treatment response (change in SHAPS score between baseline and week 9) and treatment-associated change in reward-related activity (*ρ* = −0.38, *P* = 0.060, *n* = 25). A non-significant positive correlation was observed in the placebo group (*ρ* = 0.26, *P* = 0.24, *n* = 23), leading to these independent correlations being statistically different (Fisher *r*-to-*z*: *z* = −2.16, *P* < 0.05). For the HDRS-6, our clinician-rated measure of depressive symptoms, the change in score from baseline to week 9 was significantly correlated with the change in reward-related ventral striatal activity (*ρ* = −0.40, *P* < 0.05, *n* = 25). This suggests that improvements in depressive symptoms overall, rather than anhedonia alone, were associated with greater reward-related brain activity in the ventral striatum. Change in reward-related activity between baseline and week 9 was also significantly correlated with change in HDRS-6 at 3 months (*ρ* = −0.62, *P* = 0.007, *n* = 17) and 4 months (*ρ* = −0.71, *P* = 0.003, *n* = 15) but not at the other timepoints (all *P* > 0.1). Change in reward-related activity was not significantly associated with change in SHAPS at any of the timepoints during the extended open-label phase (all *P* > 0.1).

#### Cognitive assessments and the probabilistic reward task

A subset of participants completed the probabilistic reward task (PRT) and underwent cognitive testing using the Repeatable Battery for the Assessment of Neuropsychological Status (RBANS) and the Color-Word Interference Test (CWIT) from the Delis–Kaplan Executive Function System (DKEFS). No significant differences were observed between the pramipexole and placebo groups for any of these measures (Supplementary Table 3).

#### Biomarkers

Inflammatory marker C-reactive protein (CRP) was measured in plasma, and the dopamine metabolites 3,4-dihydroxyphenylacetic acid (DOPAC) and homovanillic acid (HVA) were analyzed in plasma and cerebrospinal fluid (CSF) in a subset of participants, both before and after the 9-week RCT. CRP levels decreased significantly in the pramipexole group (*P* = 0.01) but not in the placebo group (*P* = 0.46). No significant changes were observed in the other biomarkers in either group (all *P* > 0.1). Results are shown in Supplementary Table 4.

#### Blinding integrity

Participants who reached week 9 were asked to guess the allocation arm, with the following response options: ‘placebo’, ‘pramipexole’ or ‘unsure’. A binomial test was performed to assess whether participants in the active treatment arm correctly identified their allocation more often than expected by chance. In the pramipexole group, 43.90% correctly identified their allocation, 36.59% guessed placebo and 19.51% were unsure. This proportion did not differ significantly from the 50% expected under random guessing (*P* = 0.117), indicating that participants in the pramipexole group were not better than chance at identifying their treatment, supporting effective blinding. However, in the placebo group, 66.66% correctly guessed that they were allocated to placebo, 20.51% guessed pramipexole and 12.82% were unsure. The proportion approached significance (*P* = 0.053) indicating a tendency toward better-than-chance guessing in the placebo group.

To help interpret correct guesses, we asked participants to provide reasons. Among placebo recipients who guessed correctly (*n* = 26), 46% (*n* = 12) cited lack of improvement, 31% (*n* = 8) cited lack of improvement plus absence or minimal side effects and 4% (*n* = 1) cited other reasons. For 19% (*n* = 5), no reason was available. Among pramipexole recipients who guessed correctly (*n* = 18), 33% (*n* = 6) cited improvement, 28% (*n* = 5) cited improvement and side effects and 11% (*n* = 2) cited side effects alone, and, for 28% (*n* = 5), no reason was available.

#### Sensitivity analysis

A sensitivity analysis with last observation carried forward (LOCF) to impute missing data yielded similar results as the main analysis for the primary outcome (mean difference in SHAPS: −4.06, 95% confidence interval: −6.90 to −1.23, *P* = 0.005, Hedges’ *g* = 0.63). Sensitivity analyses using LOCF yielded similar results for the secondary clinical outcomes.

#### Post hoc analysis

In a stepwise linear regression predicting change in SHAPS from baseline to week 9, treatment allocation emerged as the strongest predictor. In addition, MDD was significantly associated with an approximately 3-point greater change in SHAPS at week 9 (*P* = 0.038) compared to other affective diagnoses, primarily dysthymia. Demographic variables age, sex, body mass index (BMI), SNRI treatment and anxiety comorbidity did not contribute significantly to the model.

## Discussion

In this study, we demonstrate that pramipexole is an efficacious and safe augmentation strategy for treating anhedonia in mood disorders. By specifically including patients exhibiting significant anhedonia, our findings align with evidence linking reduced dopaminergic activity to this symptom of depression^15^.

Our results support previous observational studies^16^ and a recent large RCT (Browning et al.)^12^ while critically extending these findings by demonstrating engagement of the brain’s reward system and therapeutic effects probed by an objective measure of physical activity. We show that pramipexole not only alleviated self-reported symptoms of anhedonia but also increased activity levels as measured objectively. These findings suggest that incorporating objective outcome measures, such as accelerometry data, devoid of demand characteristics, into antidepressant trials may enhance future study designs. During the 6-month open-label extension, improvements in anhedonia, depressive symptoms and physical activity were sustained. However, because this phase lacked a control group, these findings should be interpreted descriptively and do not permit causal inference regarding efficacy but, instead, inform on longer-term safety and durability of response.

In a multisite RCT, Browning et al.^12^ recently demonstrated that pramipexole is efficacious in reducing general depressive symptoms as well as anhedonia in treatment-resistant (unipolar) MDD. Although previous studies support the antidepressant effects of pramipexole, our study is the first, to our knowledge, to specifically target patients with clinically significant anhedonia. Our findings extend this literature by focusing on a mechanistically defined subgroup characterized by prominent anhedonia. These results suggest that pramipexole may be a viable adjunctive treatment across depressive conditions (for example, MDD and dysthymia) when anhedonia is a prominent feature. However, exploratory post hoc analyses indicated that patients with MDD may be more responsive than those with dysthymia.

Although we applied a lower threshold for treatment resistance compared to Browning et al., our participants had extensive treatment histories, indicating a generally difficult-to-treat cohort. To reflect clinical practice, patients continued their ongoing antidepressants or mood stabilizers, which may have introduced pharmacological heterogeneity. Although SNRI use was imbalanced between groups, there is no evidence that SNRIs meaningfully affect pramipexole pharmacokinetics, and post hoc analyses indicated no impact on the results; however, residual confounding cannot be fully excluded. Clinical observations that pramipexole is more efficacious in patients with prominent anhedonia and low energy^8^ are consistent with evidence linking decreased dopaminergic activity in the mesocorticolimbic reward circuit to these symptoms^17–19^. Our transdiagnostic, mechanistic approach aligns with the principles of the Research Domain Criteria initiative^20^ and parallels similar trial designs, such as the RCT by Krystal et al.^21^ evaluating the antianhedonic effects of the κ receptor antagonist aticaprant. Both Krystal et al. and our study found that antidepressant treatments known to enhance dopaminergic signaling improved anhedonia and reward-related ventral striatal activation while exerting smaller effects on overall depression severity. This dissociation underscores the importance of targeted treatments and sample stratification based on specific clinical manifestations of depressive disorders rather than relying on a one-fits-all approach centered solely on global depression severity.

Unlike most antidepressant trials, we used an anhedonia-specific scale (SHAPS) as the primary outcome measure. Moreover, only patients with elevated baseline SHAPS scores were included. This predictive enrichment strategy, aligned with the drug’s mechanism of action, may help reduce heterogeneity in antidepressant trials. Clinically significant anhedonia occurs in at least half of patients with depression^22^ and often persists despite conventional treatments^23^. Interestingly, pramipexole showed similar effect sizes on both the SHAPS and the DARS, suggesting that it may improve not only consummatory but also motivational aspects of anhedonia. Our findings suggest that pramipexole may serve as a targeted treatment for clinical anhedonia across a spectrum of depressive disorders, offering a symptom-focused therapeutic approach.

Pramipexole augmentation was also associated with increased physical activity. Although the longitudinal analysis revealed a significant main effect of pramipexole on LPA, the between-group difference at week 9 did not reach statistical significance. This may partly reflect reduced compliance with accelerometer wear during the final assessment week, resulting in fewer valid observations. Accordingly, the advantages observed at intermediate timepoints (weeks 4, 5 and 7) should be interpreted as preliminary, rather than definitive, evidence of a treatment effect on physical activity. Among patients who entered the open-label extension phase, pramipexole was associated with marked increases in LPA in the original placebo group, bringing their activity levels to those of the original pramipexole group. Although accelerometers are commonly used to quantify higher levels of physical activity, in our study they were used to examine whether anhedonia-related sedentary behavior could be shifted toward greater engagement in everyday activities (that is, LPA and awake time). Although self-reports and clinician-rated measures of depressive symptoms provide valuable outcome data, they are inherently subjective and potentially susceptible to bias. By contrast, wearable technologies offer a more objective method for assessing treatment outcomes by capturing continuous, real-world behavioral data^24^. To our knowledge, this is the first antidepressant RCT to incorporate objective wearable measures alongside traditional symptom ratings.

Pramipexole was generally well tolerated, with a low dropout rate during the RCT phase. This favorable tolerability profile may partly reflect the relatively slower titration schedule applied in the trial^8^. Although the overall incidence of adverse events was high in both groups, most were mild to moderate in severity and manageable with dose adjustments. Two patients in the pramipexole group developed mild manic symptoms; both completed the trial after dose reduction or study completion with subsequent drug discontinuation. Pramipexole was not associated with an increased risk of gambling. However, transient increases in buying-related and eating-related impulse control symptoms were observed, emphasizing the importance of careful monitoring for dopaminergic side effects, including mania, sleep disturbance and impulse control symptoms.

Based on our results and the recent RCT by Browning et al.^12^, the effect sizes observed for pramipexole compare favorably with those reported for other commonly used adjunctive antidepressants^25,26^. A recent randomized open-label trial in treatment-resistant depression demonstrated that pramipexole augmentation was superior to quetiapine, a commonly prescribed second-generation antipsychotic for this condition^27^. Together, these findings further support pramipexole as a treatment option for treatment-resistant depressive states. In further support of implementing pramipexole in treatment guidelines, two recent health economic analyses indicated that pramipexole is a cost-effective intervention for bipolar and unipolar depression^28,29^. Pramipexole also represents an example of drug repositioning, a cost-effective and potentially lower-risk approach^30^, given that its tolerability and safety profiles are well established from prior use in Parkinson’s disease and restless legs syndrome.

Not all previous RCTs evaluating pramipexole for depression have demonstrated robust effects. This variability may reflect differences in study populations and dosing strategies. Given that some previous trials may have used insufficient doses, we implemented a flexible dosing strategy that allowed participants to reach a higher mean end dose compared to some earlier trials^31,32^. Clinical experience suggests that relatively higher doses of pramipexole are needed for optimal efficacy, particularly in older individuals, as dopaminergic receptor density may decline with age^33^. Although higher doses of pramipexole were associated with greater clinical improvement, separation from placebo emerged within 3 weeks across several outcome measures, suggesting efficacy across a range of doses when carefully titrated.

At the neural level, pramipexole modulated reward-related ventral striatal activation during the MID task. Consistent with findings of Krystal et al.^21^, the placebo group exhibited a decrease in ventral striatal activation, whereas this response was relatively preserved in the pramipexole group, possibly reflecting protection against task habituation^34^. In exploratory subsample analyses, which should be interpreted cautiously, we observed several significant associations between treatment-related changes in ventral striatal activation and long-term improvement in both anhedonia and overall depressive symptoms. These findings are consistent with the hypothesis that pramipexole may normalize reward-related and motivation-related neural function and raise the possibility of a biologically defined subgroup of patients who may particularly benefit from this treatment. To our knowledge, this is among the first studies in depression to experimentally evaluate the impact of a dopaminergic intervention on reward-related neural circuitry. In a previous positron emission tomography study, Whitton et al.^35^ demonstrated that decreased pretreatment ventral striatum D2/D3 receptor availability and blunted dopamine release were associated with response to pramipexole. These results further support this dopaminergic subgroup hypothesis. Taken together, the present fMRI findings provide evidence of neural target engagement by pramipexole and indicate that modulation of ventral striatal function during reward processing may contribute to its therapeutic effects on anhedonia.

In exploratory analyses, we found that pramipexole significantly reduced levels of the inflammatory marker CRP, an effect not observed in the placebo group. This finding replicates results from our previous pilot study^9^ and is consistent with preclinical evidence suggesting that pramipexole may modulate immune responses^36^. Future studies should further investigate the potential antiinflammatory effects of pramipexole and their relationship to dopaminergic neurotransmission.

This study has several limitations that should be considered when interpreting the findings. Few psychiatric RCTs formally evaluate blinding integrity, despite growing consensus that this should be standard practice^37^. In our trial, we prospectively assessed and transparently reported blinding. Approximately two-thirds of the patients in the placebo group correctly guessed their treatment allocation, whereas guess accuracy in the pramipexole group was near chance. This asymmetry represents a study limitation, as it may introduce expectancy effects and bias subjective symptom ratings. Participants most often attributed correct guesses to perceived clinical improvement (or lack thereof), sometimes in combination with the presence or absence of side effects. These findings highlight a broader challenge in RCTs relying on subjective outcomes: guess accuracy often tracks perceived response and, to a lesser extent, tolerability rather than systematic unblinding. To mitigate this limitation, we incorporated objective outcome and target engagement measures, including accelerometry and fMRI, which enhance the internal validity and interpretability of our findings. Although the inclusion of these exploratory outcome measures strengthens mechanistic interpretation, these findings should be considered supportive, rather than definitive, evidence of target engagement. Another limitation is that we did not adjust for multiplicity across secondary outcomes, increasing the risk of type I error and warranting cautious interpretation of these findings. At the same time, the mechanistic analyses were conducted in relatively small subsamples, reducing statistical power and increasing the risk of type II error. Together, these limitations indicate that both false-positive and false-negative findings are possible, underscoring the need for replication of these exploratory subanalyses in larger, adequately powered studies. Moreover, the lack of statistically significant differences in dichotomized SHAPS remission and response outcomes may reflect reduced statistical power and the inherent limitations of dichotomizing continuous measures. The use of a predictive enrichment strategy, enrolling only patients with prominent anhedonia, may limit generalizability to broader depression populations. However, this approach was deliberately chosen to align the study sample with pramipexole’s mechanism of action and to reduce clinical heterogeneity. Accordingly, the findings should be interpreted as primarily applicable to patients with clinically significant anhedonia.

In conclusion, this RCT demonstrates that the dopamine agonist pramipexole is an effective, safe and well-tolerated augmentation strategy for improving anhedonia in patients with mood disorders characterized by prominent anhedonic symptoms.

## Methods

Comprehensive information on the study protocol was previously published^38^.

### Trial design

This was an academic single-center trial conducted in Lund, Sweden. Patients were randomized to receive either add-on flexible dose pramipexole or identical placebo for 9 weeks, followed by an open-label extension phase for six additional months. The primary outcome measure was absolute change in the SHAPS total score between baseline and week 9, calculated using the Likert scale (1–4 points per item, total scores 14−56). All patients were asked to wear an accelerometer during the trial period with the option to opt out if inconvenient.

Optional substudies were conducted in a subset of participants at baseline and week 9, including 7-Tesla fMRI with the MID task, cognitive assessments, the PRT and blood and CSF sampling. Participation in these substudies was voluntary and associated with monetary compensation. Patients could take part in one or more of these substudies. The PRT was introduced in 2024, approximately halfway through the study.

### Intervention

Extended-release pramipexole tablets from STADA Nordic were labeled and repackaged consistent with European Union guidelines and Good Manufacturing Practice (GMP). Identical placebo tablets were produced by Ardena in line with GMP. All tablets were packed in boxes and assigned randomized numbers. Only Ardena and an unblinded research nurse (who was not involved in patient assessments) had access to the code list connecting the randomization numbers to treatment allocation. Pramipexole tablets were taken once daily, preferably at nighttime. Patients, research physicians and nurses involved in patient assessments were blinded for treatment allocation. Because both pramipexole and placebo were packaged by Ardena, eight different logs for the study medication were created, and four different strengths were ordered and combined to achieve the correct dosing. For each strength, both pramipexole and placebo were available. These logs were accessible only to the unblinded staff members who packed and labeled all boxes with the participants’ randomization numbers. The unblinded staff members were not involved in symptom assessments. Blinded study personnel then administered these to the patients using a flexible dosing schedule (Supplementary Table 5). An unblinded statistician at Clinical Studies Sweden Forum South prepared the random allocation sequence and established the randomization list. Apart from the preparation of the allocation sequence, the statistician was not involved in the study. The block size varied randomly between 4 and 6, and the order within the blocks was generated randomly using a computer-based algorithm. Only the statistician who created the randomization list was aware of the random variation in block size and was also the only person who had access to the randomization list. At the week 9 visit, after clinical ratings, patients were asked if they thought that they had been allocated to pramipexole or to placebo. At the start of the study, participants had only two options: pramipexole or placebo. During the study, several patients described their guess as a ‘coin toss’. We, therefore, added the possibility of answering ‘unsure’.

To maintain blinding in the RCT, a separate team of study physicians and nurses conducted the open-label extension phase. Thus, none of the physicians responsible for patient assessments in the RCT participated in the open-label phase, ensuring that they remained unaware of any side effects or efficacy signals that could compromise blinding during the randomized phase. All study medication used during the follow-up study was purchased from the pharmaceutical company STADA.

We used an individually tailored dose titration schedule based on recommended dose escalation strategies for Parkinson’s disease but adjusted according to side effects and efficacy (Supplementary Table 5). We increased pramipexole doses weekly and decreased the dose in the event of intolerable side effects and waited approximately 1 week before a new attempt of dose escalation was made. If patients had a Clinical Global Impression (CGI) Severity score of ≤2, dose escalation was discontinued for the remainder of the study, provided they did not show increased symptom burden at subsequent visits.

### Patient population

Patients were recruited via advertisements or clinical referrals from psychiatric or primary care units. They were prescreened via telephone and then screened on site to determine if they fulfilled eligibility criteria.

Inclusion criteria were age 18–75 years; a current diagnosis of MDD, dysthymia or bipolar disorder with a depressive episode; ongoing treatment with at least one antidepressant or mood-stabilizing medication for ≥4 weeks; and a history of at least one adequate antidepressant trial without remission. All participants provided written informed consent prior to study participation.

In addition, participants were required to have clinically significant anhedonia, defined as scores of 3 or 4 on at least three items of the SHAPS^13^.

This recruitment strategy aligns with the conceptualization of anhedonia as a cross-diagnostic symptom with a potentially shared pathophysiology involving reward circuit dysfunction and reduced dopaminergic activity.

Study participants underwent psychiatric and somatic evaluations including standard laboratory screening. Primary diagnoses were determined through diagnostic interviews conducted by psychiatry residents or specialists, combining relevant sections of the structured Mini International Neuropsychiatric Interview (MINI) with a review of each patient’s clinical history^39^. Exclusion criteria included pregnancy, breastfeeding or planned pregnancy; high suicide risk based on clinical assessment; substance use disorder within the past 6 months; current psychotic disorder; emotionally unstable personality disorder; compulsory psychiatric care; impulse control disorders (including binge-eating disorder) or attention-deficit/hyperactivity disorder with hyperactivity; and cognitive impairment (including intellectual disability or dementia) affecting the ability to provide informed consent.

Participants with significant medical comorbidities were excluded, including renal impairment (estimated glomerular filtration rate (eGFR) <50 ml min^−1^ 1.73 m^−^^2^), symptomatic cardiovascular disease (New York Heart Association (NYHA) class II or higher), hepatic insufficiency, Parkinson’s disease, active cancer not in remission for more than 1 year or previous bariatric surgery affecting drug absorption.

Additional exclusions included recent initiation of psychotherapy (<6 weeks) or planned initiation during the trial; ongoing electroconvulsive therapy (ECT), ketamine or repetitive transcranial magnetic stimulation (except maintenance treatment); use of medications affecting pramipexole pharmacokinetics or with similar or antagonistic dopaminergic mechanisms (except low-dose quetiapine ≤150 mg per day^40^); known hypersensitivity to the study drug; participation in other interventional studies; and any condition judged by the investigators to compromise safety, compliance or evaluability.

Patients were eligible for the 6-month open-label extension phase if they had received pramipexole during the RCT phase, wanted to continue treatment and were still not meeting any exclusion criteria. Participants who had received placebo in the RCT could also enter the extension and receive pramipexole, provided they continued to meet all original RCT eligibility criteria, and no exclusion criteria, at the end of the randomized phase. Participants who were already receiving pramipexole continued at the same dose unless dose reduction was warranted due to adverse events or dose escalation was indicated based on insufficient clinical response with a maximum dose of 4.5-mg salt. Participants who had previously received placebo underwent dose titration with pramipexole, in accordance with the titration schedule used in the RCT. These participants were followed-up by an additional phone call after 2 weeks to assess tolerability.

### Assessments

At all study visits (both the RCT phase and the open-label extended phase), participants were assessed on site by a study physician. At each in-person visit, the physician conducted clinical evaluations and participants completed self-rating scales, with a focus on both efficacy and tolerability of the intervention as well as any need for dose adjustments.

#### Rating scales

For clinical rating scales, data were collected from baseline, week 3, week 6 and week 9 during the randomized controlled phase and monthly during the extension open-label phase. The SHAPS^41^ was scored from 1 to 4 on each item, with a total score of 56. The SHAPS is a commonly used self-rating scale that mainly covers the consummatory phase of anhedonia. Response (≥50% reduction in SHAPS scores) was evaluated using the original dichotomized method (1–2 = 0, 3–4 = 1). Remission was defined according to the dichotomized scoring method, with a score of <3 indicating remission. We used the self-rating version of MADRS-S, and response was defined as ≥50% improvement and remission as ≤10 (ref. ^42^) at week 9. The HDRS-6 (ref. ^43^) is an expert rating scale including six core depression items from the HDRS: ‘depressed mood’, ‘feelings of guilt’, ‘work and interest’, ‘psychic anxiety’, ‘general somatic symptoms’ and ‘retardation’. This subscale has shown higher sensitivity for antidepressant signals than the full HDRS^44^. In addition to the SHAPS, anhedonia was also evaluated using the DARS^45^ as it includes additional anhedonia domains and separates the motivational and consummatory phases of anhedonia. The AES-S is a self-rating scale evaluating apathy symptoms that are conceptually related to anhedonia^46^. Generalized anxiety symptoms were assessed with the GAD-7 (ref. ^47^). Insomnia symptoms were evaluated with the ISI^48^. Quality of life was measured with the BBQ^49^.

#### Accelerometry

Physical activity outcomes were assessed using accelerometers (ActiGraph GT3X-BT; ActiGraph) as further described in the study protocol^38^. The accelerometers were initialized to sample three-dimensional acceleration data at 30 Hz, with ActiGraph’s idle sleep mode enabled. Study participants were instructed to wear the accelerometer day and night for 10 weeks in total, on the non-dominant wrist. The first week was used as a baseline measure before the intervention started. During the remaining 9 weeks, raw data were extracted every third week using ActiLife software version 6.13.4 (ActiGraph). During the open-label follow-up phase, participants who consented received an accelerometer at the month 3 and month 5 visits and wore it for seven consecutive days on each occasion, following the same instructions as in the RCT phase. Data were extracted as gt3x files and processed in R (version 4.5.0) with the R package GGIR (version 3.2.6) as previously described^50^. Data were aggregated using the Euclidean Norm Minus One *g* (ENMO)^51^ metric and averaged over 1-second epochs. A day was considered valid if it included at least 16 hours of wear time across the full 24-hour period and covered a minimum of two-thirds of waking hours^52^. Non-wear and awake time were estimated using the default algorithms in GGIR^51,53^. Data were analyzed for weekly physical activity. Patients having fewer than four valid days during one of the weeks did not reach the valid week criteria and were excluded from physical activity analyses at that timepoint, consistent with established practice^14^. To estimate SED and physical activity at various intensities during awake time, ENMO cutpoints developed for the non-dominant wrist by Hildebrand et al.^54,55^ were applied (SED < 44.8 mg; LPA: 44.8−100.6 mg; MVPA > 100.6 mg). For physical activity analysis, a measurement day was defined as the period from wake-up to wake-up. A configuration file with all settings used for GGIR data processing is available as Supplementary Data 1.

In addition to the accelerometry outcomes reported in this paper, the protocol also specified the following secondary variables that are not reported here: detailed sleep parameters (for example, sleep latency, wake after sleep onset, deep sleep and sleep efficiency), step count and distributional measures of movement patterns.

#### fMRI and MID task

Our a priori aim was to assess treatment effects on ventral striatal activity during reward anticipation. To accomplish this, we employed a simplified MID task focusing on appetitive processing^9,56,57^. The task design followed earlier simplified incentive delay paradigms: on each trial, participants viewed one of two cues signaling either a high (€0.50) or a low (€0.01) potential reward. Reward delivery depended on response speed to a subsequent target cue, with rewards administered on 75–80% of trials. Scans were acquired using a 7-Tesla whole-body magnetic resonance scanner (Philips Achieva; Philips Medical Systems) using a 32-channel head coil (Nova Medical). Anatomical three-dimensional T1-weighted images were acquired with the following parameters: repetition time (TR) = 5.0 ms, echo time (TE) = 1.97 ms, inversion time (TI) = 1,200 ms, shot interval = 3,500 ms, Turbo factor = 450, flip angle = 6°, voxel size = 1.0 × 1.0 × 1.0 mm^3^. BOLD fMRI was collected using a simultaneous multislice echo-planar imaging (SMS-EPI) sequence with the following parameters: TR = 1,500 ms, TE = 25 ms, flip angle = 5°, voxel size = 2.0 × 2.0 × 2.0 mm^3^, slice gap = 0.2 mm, slices = 50, multislice acceleration factor = 2, SENSE acceleration factor = 3. In addition, a fluid-attenuated inversion recovery (FLAIR) sequence (TR = 6,000 ms, TE = 316 ms, TI = 1,825 ms, flip angle =55°, voxel size = 1.0 × 1.0 × 1.0 mm^3^) was acquired and read by an experienced neuroradiologist; no relevant lesions were detected. Moderate white matter lesions thought to represent small vessel disease were present in two individuals (placebo, *n* = 1; pramipexole, *n* = 1) and more severe in one individual (pramipexole).

Preprocessing was performed using fMRIPrep 23.1.4 (ref. ^58^) (RRID: SCR_016216), which is based on Nipype 1.8.6 (ref. ^59^) (RRID: SCR_002502) (see Supplementary Data 2 for details). Modeling of BOLD activity was performed using SPM12 (SPM12, https://fil.ion.ucl.ac.uk/spm/software/spm12/). Volumes were first smoothed using a 6-mm full width at half maximum (FWHM) Gaussian filter. First-level models were then estimated for each individual and session with six movement parameters as well as onsets and durations of all stimuli presentations (high-reward and low-reward cues and outcomes, target presentation and reward anticipation) convolved with the canonical hemodynamic response function from SPM12. The contrast between activity during the anticipation phase for high-reward cues versus low-reward cues was used for group-level analysis. For each participant, average activation statistics were extracted from a bilateral ROI encompassing the ventral striatum (Fig. 3) before and after treatment. The ROI was created using the accumbens region from the probabilistic Harvard-Oxford Subcortical Atlas. Given the spatial uncertainty of fMRI activation patterns, we chose to use a liberal definition of this brain region by employing non-thresholded ROI similar to a previous study by Krystal et al.^21^.

#### Cognitive assessments

Testing was administered by trained staff in a standardized, quiet setting, and scoring followed established test manuals.

Cognitive function was assessed using the RBANS^60^ and the CWIT^61^ from the DKEFS. The RBANS evaluates multiple cognitive domains, including memory, attention, language and visuospatial abilities, and alternate forms (A and B) were used to minimize practice effects. Several RBANS subtests showing substantial ceiling effects were excluded from analyses. Executive function was assessed using CWIT, including contrast scores to isolate inhibition and cognitive flexibility.

The protocol also included the Wechsler Adult Intelligence Scale, Fourth Edition (WAIS-IV^62^), the Conners Continuous Performance Test (CPT) and the Perceived Deficits Questionnaire (PDQ); however, results from these measures are not presented in this paper. WAIS-IV was only assessed at baseline to ensure that groups were similar in general cognitive ability. WAIS-IV scores and education history showed that groups were balanced with regard to education and general cognitive ability. CPT data were not collected due to technical difficulties. PDQ results are not reported because the Swedish version of this scale has not been validated.

#### PRT

Reward responsiveness was assessed using the PRT^63^. In each trial, participants discriminated between two visually similar stimuli (short versus long mouth) by pressing one of two buttons. Correct identification of one stimulus (‘rich’) was rewarded three times more frequently than the other (‘lean’), leading to the development of a response bias toward the more frequently rewarded stimulus. Reward learning was operationalized as the change in response bias from the initial to the final block (delta response bias), reflecting sensitivity to reward.

#### Biomarkers

Blood and CSF samples were collected in the morning under fasting conditions. Dopamine metabolites HVA and DOPAC were quantified at Pronexus Analytical AB using ultra-high-performance liquid chromatography coupled with electrospray tandem mass spectrometry, a validated method described in detail elsewhere^64^. Calibration curves were constructed over a range of 2.5–2,000 ng ml^−1^, with limits of quantification of 8 ng ml^−1^ for DOPAC and 7 ng ml^−1^ for HVA. The coefficient of determination (*R*^2^) for the calibration curves within this range was 0.9945 for DOPAC and 0.9927 for HVA.

Plasma CRP levels were measured in duplicate using a high-sensitivity electrochemiluminescence-based multiplex immunoassay (U-PLEX Human CRP Assay; Meso Scale Discovery). The intra-assay coefficient of variation was 19.74%, and the lower limit of detection (LLOD) was 0.66 pg ml^−1^.

#### Safety assessments

At each visit, including telephone follow-ups, participants were systematically asked about adverse events. All reported adverse events were documented in an individual patient log. Each adverse event was evaluated by the study physician with regard to (1) severity, (2) likelihood of relatedness to pramipexole and (3) whether it met criteria for a serious adverse event. The study physicians regularly discussed adverse event assessments to promote consistent and harmonized evaluation across patients. Adverse events that were ongoing at the end of the RCT were prospectively followed throughout the open-label follow-up phase until resolution or until study completion in cases of non-resolution.

To evaluate adverse events related to impulsivity, gambling behavior and mood instability, we extracted relevant self-rating items from the QUIP^65^, the PGSI^66^ and the YMRS^67^. Modifications were implemented to reduce administration time and improve compatibility, and the prefix ‘M-’ was added to each scale to denote these changes. Study physicians reviewed self-ratings of these items and, if there were positive responses, conducted interviews to determine if these symptoms were of clinical relevance.

##### M-QUIP

The original QUIP contains four domains assessing different impulsive−compulsive behaviors. In our adaptation, the final question from each domain, specifically targeting Parkinson-related medication, was removed. Additionally, the entire fourth domain concerning hobbyism (behavioral addictions) and punding (non-goal-oriented and repetitive behaviors) was excluded. Furthermore, the response format was simplified by reducing the number of response options from five to four.

##### M-PGSI

In the modified PGSI, items 3, 4, 6 and 8 were omitted to avoid repetition. The remaining items were retained in their original format.

##### M-YMRS

The YMRS was adapted both in structure and mode of administration. Items 3, 7, 9 and 11 were excluded. Additionally, the scale was administered as a self-report rather than through clinician interview, and the number of response options per item was reduced from five to four.

### Oversight

The study was preregistered at ClinicalTrials.gov (NCT05355337 and NCT05825235) and was approved by the Swedish Ethical Review Authority (reference number 2023-01927-02) and the Swedish Medical Products Agency (EudraCT 2022-001563-26 and 2022-502270-17-00). Clinical Studies Sweden Forum South monitored the study in accordance with Good Clinical Practice. Patients signed an informed consent form after receiving oral and written information about the study. The Department of Psychiatry at Skåne University Hospital was the sponsor.

### Statistical analyses

An independent statistician performed the analyses of the primary and secondary outcomes that were assessed using rating scales.

In our original power calculation, which was performed for MMRM treatment main effect from week 3 to endpoint, we estimated an effect size for pramipexole of 0.27, based on reports from other antidepressant studies^68^. This corresponds to a clinically significant MADRS score improvement of approximately 4 points, which aligns with a ≥4-point change on the SHAPS. We based the correlation coefficient between repeated MADRS measures on data from our previous pilot study (*r* = 0.5). To achieve 80% power with an *α* of 0.05, we initially calculated that 74 participants were necessary. Considering an estimated 5% dropout rate from our pilot data, we aimed to recruit 80 participants to start treatment. A prespecified interim analysis was conducted by an independent statistician in June 2024. The number of dropouts was controlled, and the sample size was recalculated with an updated standard deviation. Based on these results, the recruitment target was increased by five participants. The interim analysis did not affect any other methodological decisions. Eighty patients with complete efficacy data through week 9 were needed. The power calculation was based on the SHAPS as the primary outcome and was not specifically tailored to the secondary accelerometry or fMRI analyses, which were conducted in smaller subsamples.

Statistical analyses of longitudinal outcome data were conducted using MMRM, including fixed effects, treatment group (placebo versus pramipexole), time (week) and their interaction (group × time) plus the baseline value of the respective outcome to adjust for individual differences at study entry. The unstructured covariance structure was chosen. Hedges’ *g* value effect size was calculated. Pairwise comparisons between treatment groups at each timepoint were performed on change scores (delta from baseline), using EMMs derived from the fitted mixed-effects model. The primary estimand was the between-group difference in change in SHAPS total score from baseline to week 9, as prespecified in the statistical analysis plan (SAP). The MMRM treatment main effect was reported as a secondary, supportive estimand, making use of all available longitudinal data.

Because no treatment effect was expected before week 3, accelerometry data were analyzed using both data from all weeks and models excluding weeks 1 and 2.

The fMRI MID task outcome was analyzed using an MMRM, including pairwise between‑group comparisons with adjustment for baseline differences. PRT, RBANS and DKEFS outcomes were analyzed using the same MMRM framework. CRP and dopamine metabolite biomarkers were analysed using Wilcoxon signed‑rank tests, as prespecified in the protocol. In the open-label extension phase, we report descriptive statistics for the primary and secondary outcomes at each timepoint.

No formal adjustment for multiple testing was applied to secondary or exploratory outcomes, in accordance with the SAP.

In the SAP, we prespecified that primary and secondary efficacy outcomes for pramipexole would be analyzed in a modified ITT population, defined as all randomized patients with at least one follow-up assessment (that is, all patients who completed the week 3 visit). In accordance with this definition, two participants without any post-baseline data and one randomized but ineligible participant were excluded from the primary efficacy analyses. All three are, however, included in the CONSORT flow chart. The two participants who did not complete the week 3 assessment contributed no information to the primary endpoint, as no follow-up data for any of the outcome measures are available from which a change score could be derived.

Non-parametric correlation analyses (Spearman) were conducted to examine associations between ventral striatal activation and SHAPS change scores.

SPSS Statistics versions 28 and 30 (IBM Corporation), SAS Enterprise Guide 8.3 with SAS PROC MIXED and R (version 4.5.0) with the nlme (version 3.1-168) and emmeans (version 1.11.2-8) packages were used for statistical analysis. Data visualization was performed in R using ggplot2 (version 4.0.3). The statistical significance level was set to *P* < 0.05 using two-tailed tests.

### Reporting summary

Further information on research design is available in the Nature Portfolio Reporting Summary linked to this article.

## Online content

Any methods, additional references, Nature Portfolio reporting summaries, source data, extended data, supplementary information, acknowledgements, peer review information; details of author contributions and competing interests; and statements of data and code availability are available at 10.1038/s41591-026-04465-9.

## Supplementary information

Supplementary Information Supplementary Tables 1–5, Fig. 1, Data 1 and 2, study protocol, statistical analysis plan and CONSORT checklist.

Reporting Summary

Peer Review File

## Data Availability

The datasets generated and analyzed during this study contain sensitive personal information and are protected under the European Union General Data Protection Regulation (GDPR). Deidentified participant data underlying the findings of this study will be made available from the corresponding author, subject to approval by the relevant institutional ethics committee and in accordance with GDPR and local data protection regulations. Requests will be acknowledged within 2 weeks. After approval and completion of any required data-sharing agreements, data will typically be made available within 4–8 weeks. The SAP was uploaded at ClinicalTrials.gov (NCT05355337), and the study protocol is published alongside the main paper as supplementary material.

## Extended data

**Extended Data Table 1:** Descriptive statistics of primary and secondary outcome rating scales in ITT sample

Outcome	Time Point	Mean Pramipexole Group (SD)	Mean Placebo Group (SD)
SHAPS	Baseline	41.54 (5.57)	39.51 (5.16)
	Week 3	37.51 (7.53)	38.66 (5.99)
	Week 6	36.17 (7.74)	37.25 (7.14)
	Endpoint	34.98 (8.04)	37.26 (7.43)
HDRS-6	Baseline	7.37 (2.47)	7.20 (2.15)
	Week 3	5.54 (2.74)	6.66 (2.24)
	Week 6	5.00 (2.37)	5.85 (2.56)
	Endpoint	4.56 (2.57)	5.36 (2.31)
MADRS-S	Baseline	27.63 (6.43)	25.73 (6.99)
	Week 3	22.49 (7.64)	23.17 (7.28)
	Week 6	20.66 (7.81)	21.25 (7.66)
	Endpoint	19.95 (7.78)	20.67 (8.86)
DARS	Baseline	23.10 (10.34)	25.44 (12.10)
	Week 3	25.98 (12.52)	24.78 (13.04)
	Week 6	28.63 (13.77)	25.20 (12.64)
	Endpoint	30.76 (14.22)	25.92 (13.36)
AES-S	Baseline	50.71 (5.96)	48.66 (8.45)
	Week 3	46.68 (7.56)	47.61 (7.86)
	Week 6	44.90 (7.49)	45.90 (8.27)
	Endpoint	42.54 (7.51)	46.59 (8.36)
GAD-7	Baseline	9.98 (4.81)	8.98 (5.13)
	Week 3	7.66 (4.94)	6.68 (4.17)
	Week 6	6.76 (4.68)	6.05 (3.93)
	Endpoint	7.20 (4.93)	5.69 (3.95)
ISI	Baseline	12.71 (5.44)	13.41 (6.12)
	Week 3	12.22 (5.57)	12.05 (5.39)
	Week 6	11.41 (6.07)	11.75 (6.08)
	Endpoint	10.88 (5.94)	11.72 (5.99)
BBQ	Baseline	16.59 (13.19)	19.07 (14.52)
	Week 3	23.41 (15.83)	20.46 (15.23)
	Week 6	25.39 (16.09)	25.75 (15.55)
	Endpoint	30.24 (20.96)	26.38 (17.82)

Descriptive statistics for primary and secondary outcome rating scales in the intention-to-treat population of the randomized controlled trial (baseline–week 3: n = 82 [pramipexole n = 41, placebo n = 41]; week 6: n = 81 [pramipexole n = 41, placebo n = 40]; week 9: n = 80 [pramipexole n = 41, placebo n = 39]). Total scores are summarized as mean values with standard deviations in parentheses.

**Extended Data Table 2:** Descriptive statistics of primary and secondary outcome rating scales in the open-label extended phase

Outcome	Time Point	Mean Pramipexole Group (SD)	Mean Placebo Group (SD)
SHAPS	Month 1	33.33 (8.72)	38.30 (6.89)
	Month 2	30.93 (9.90)	37.27 (8.35)
	Month 3	30.50 (9.26)	37.36 (7.85)
	Month 4	29.83 (10.40)	35.58 (7.90)
	Month 5	28.00 (10.66)	34.00 (8.34)
	Endpoint	29.10 (10.72)	34.25 (8.77)
HDRS-6	Month 1	2.77 (2.03)	4.61 (3.23)
	Month 2	2.40 (2.24)	3.64 (1.56)
	Month 3	2.69 (2.81)	3.64 (2.75)
	Month 4	1.83 (1.81)	3.16 (2.32)
	Month 5	1.61 (2.06)	3.18 (2.79)
	Endpoint	1.48 (1.99)	2.44 (2.19)
MADRS-S	Month 1	17.07 (8.07)	22.00 (8.38)
	Month 2	16.10 (9.54)	20.55 (7.71)
	Month 3	15.38 (10.56)	20.36 (9.78)
	Month 4	13.75 (9.66)	17.68 (8.82)
	Month 5	12.96 (10.20)	18.41 (9.01)
	Endpoint	13.05 (10.37)	16.00 (9.00)
DARS	Month 1	35.92 (16.17)	41.07 (13.95)
	Month 2	28.80 (16.69)	42.82 (12.96)
	Month 3	27.12 (17.37)	44.64 (10.37)
	Month 4	32.71 (18.88)	30.91 (13.72)
	Month 5	25.65 (16.96)	38.94 (16.50)
	Endpoint	26.50 (18.18)	38.25 (13.74)
AES-S	Month 1	40.40 (7.01)	48.30 (7.07)
	Month 2	38.23 (9.33)	46.00 (7.84)
	Month 3	38.12 (9.67)	46.00 (7.50)
	Month 4	37.33 (9.64)	43.05 (10.55)
	Month 5	35.91 (8.83)	43.18 (10.27)
	Endpoint	36.19 (10.30)	44.25 (9.57)
GAD-7	Month 1	10.93 (6.49)	11.91 (7.26)
	Month 2	10.10 (6.82)	11.23 (7.89)
	Month 3	9.77 (5.64)	10.73 (7.75)
	Month 4	9.50 (5.79)	10.26 (6.58)
	Month 5	8.17 (6.15)	10.06 (6.36)
	Endpoint	8.55 (6.75)	10.25 (6.85)
ISI	Month 1	7.13 (5.98)	4.91 (4.29)
	Month 2	6.63 (6.06)	5.45 (4.23)
	Month 3	5.85 (6.16)	5.82 (5.16)
	Month 4	4.71 (5.13)	5.58 (4.06)
	Month 5	4.61 (6.06)	4.78 (4.86)
	Endpoint	3.82 (4.94)	4.12 (3.91)
BBQ	Month 1	35.10 (20.93)	21.48 (13.12)
	Month 2	42.20 (25.82)	25.05 (17.44)
	Month 3	42.77 (26.54)	24.00 (16.01)
	Month 4	47.96 (29.00)	31.11 (20.49)
	Month 5	48.74 (29.44)	26.50 (21.19)
	Endpoint	46.00 (31.20)	27.81 (22.09)

Descriptive statistics for primary and secondary outcome rating scales during the open-label extension phase in the intention-to-treat population. Total scores are presented as means with standard deviations in parentheses. All participants received pramipexole during the open-label phase; however, data are stratified according to treatment allocation during the randomized controlled phase (pramipexole vs placebo). Sample sizes by time point (former pramipexole vs former placebo group) were as follows: month 1, 30 vs 23; month 2, 30 vs 22; month 3, 26 vs 22; month 4, 24 vs 19; month 5, 23 vs 17; and month 6, 21 vs 16.

**Extended Data Table 3:** Summary of statistical analyses for primary and secondary outcome rating scales in the RCT

Outcome			Change with Treatment		Group Differences			
	Time	Group	LS mean (SE)	95% CI	Est (SE)	95% CI	p-value	Effect Size
SHAPS	W3-BL	Pramipexole	−3.94 (0.83)	−5.57; −2.30				
		Placebo	−0.95 (0.64)	−2.21; 0.31	−2.99 (1.05)	−5.07; −0.91	0.0052	−0.63
	W6-BL	Pramipexole	−5.28 (0.92)	−7.10; −3.45				
		Placebo	−2.40 (0.88)	−4.15; −0.66	−2.88 (1.28)	−5.41; −0.34	0.0265	−0.50
	W9-BL	Pramipexole	−6.47 (1.10)	−8.65; −4.30				
		Placebo	−2.44 (0.93)	−4.28; −0.60	−4.04 (1.45)	−6.89; −1.18	0.0059	−0.62
HDRS6	W3-BL	Pramipexole	−1.80 (0.32)	−2.44; −1.16				
		Placebo	−0.59 (0.28)	−1.14; −0.034	−1.22 (0.43)	−2.06; −0.37	0.0051	−0.63
	W6-BL	Pramipexole	−2.34 (0.32)	−2.97; −1.70				
		Placebo	−1.39 (0.38)	−2.14; −0.64	−0.95 (0.50)	−1.93; 0.033	0.0582	−0.42
	W9-BL	Pramipexole	−2.78 (0.37)	−3.50; −2.06				
		Placebo	−1.92 (0.38)	−2.67; −1.17	−0.86 (0.53)	−1.90; 0.18	0.1058	−0.36
MADRS-S	W3-BL	Pramipexole	−5.02 (0.77)	−6.53; −3.50				
		Placebo	−2.71 (0.72)	−4.12; −1.29	−2.31 (1.05)	−4.40; −0.23	0.0299	−0.49
	W6-BL	Pramipexole	−6.85 (0.98)	−8.78; −4.92				
		Placebo	−4.63 (0.90)	−6.41; −2.85	−2.22 (1.33)	−4.85; 0.42	0.0984	−0.37
	W9-BL	Pramipexole	−7.55 (1.07)	−9.67; −5.44				
		Placebo	−5.30 (1.03)	−7.34; −3.26	−2.25 (1.49)	−5.19; 0.69	0.1321	−0.34
DARS	W3-BL	Pramipexole	2.77 (1.24)	0.31; 5.23				
		Placebo	−0.50 (1.09)	−2.66; 1.65	3.27 (1.66)	−0.0043; 6.55	0.0503	0.44
	W6-BL	Pramipexole	5.43 (1.59)	2.28; 8.57				
		Placebo	−0.033 (1.40)	−2.66; 1.65	5.46 (2.12)	1.26; 9.66	0.0111	0.57
	W9-BL	Pramipexole	7.55 (1.82)	3.96; 11.14				
		Placebo	1.42 (1.40)	−1.34; 4.18	6.13 (2.29)	1.60; 10.66	0.0083	0.59
AES-S	W3-BL	Pramipexole	−3.77 (1.13)	−6.00; −1.54				
		Placebo	−1.38 (0.75)	−2.86; 0.095	−2.39 (1.36)	−5.07; 0.30	0.0812	−0.39
	W6-BL	Pramipexole	−5.55 (1.09)	−7.70; −3.40				
		Placebo	−3.19 (0.82)	−4.81; −1.58	−2.36 (1.36)	−5.05; 0.34	0.0862	−0.38
	W9-BL	Pramipexole	−7.92 (1.16)	−10.20; −5.63				
		Placebo	−2.94 (0.90)	−4.72; −1.16	−4.97 (1.47)	−7.88; −2.07	0.0009	−0.75
GAD-7	W3-BL	Pramipexole	−2.14 (0.58)	−3.28; −0.99				
		Placebo	−2.49 (0.37)	−3.21; −1.76	0.35 (0.69)	−1.01; 1.70	0.6137	0.11
	W6-BL	Pramipexole	−3.04 (0.53)	−4.09; −1.99				
		Placebo	−3.09 (0.55)	−4.17; −2.01	0.053 (0.76)	−1.46; 1.56	0.9443	0.016
	W9-BL	Pramipexole	−2.60 (0.57)	−3.72; −1.48				
		Placebo	−3.48 (0.49)	−4.45; −2.50	0.88 (0.75)	−0.61; 2.36	0.2459	0.26
ISI	W3-BL	Pramipexole	−0.57 (0.61)	−1.79; 0.64				
		Placebo	−1.29 (0.51)	−2.30; −0.28	0.72 (0.80)	−0.86; 2.30	0.3703	0.20
	W6-BL	Pramipexole	−1.38 (0.77)	−2.90; 0.14				
		Placebo	−1.51 (0.49)	−2.48; −0.54	0.13 (0.91)	−1.67; 1.94	0.8839	0.032
	W9-BL	Pramipexole	−1.92 (0.82)	−3.54; −0.29				
		Placebo	−1.78 (0.48)	−2.74; −0.82	−0.14 (0.96)	−2.02; 1.75	0.8875	−0.031
BBQ	W3-BL	Pramipexole	6.64 (1.85)	2.98; 10.30				
		Placebo	1.68 (1.35)	−0.99; 4.34	4.96 (2.30)	0.43; 9.50	0.0321	0.48
	W6-BL	Pramipexole	8.61 (2.04)	4.58; 12.65				
		Placebo	6.96 (1.83)	3.34; 10.58	1.65 (2.75)	−3.77; 7.08	0.5484	0.13
	W9-BL	Pramipexole	13.47 (2.87)	7.80; 19.14				
		Placebo	7.91 (2.01)	3.95; 11.88	5.55 (3.51)	−1.37; 12.48	0.1152	0.35

Adjusted means (least-squares means), 95% confidence intervals, model estimates, p-values, and effect sizes (Hedges’ g) are reported. Pairwise comparisons between treatment groups at each time point were performed on change scores (delta from baseline), using estimated marginal means derived from the linear mixed model repeated measures. All tests were two-sided and no adjustments were made for multiple comparisons.

## Code availability

The SAS syntax and R scripts used for the analyses in this study are publicly available under the MIT license at https://github.com/UnitforBAPP/PRIME-PRAXOL_RCT/.
