# Supplementary material for: Efficacy and target engagement of dopamine agonist pramipexole for anhedonic depression: a randomized placebo-controlled trial
Source: Nat Med. 2026 Jun 12;32(7):2570–8. doi: 10.1038/s41591-026-04465-9 (PMC13375643; doi:10.1038/s41591-026-04465-9)
Supplement: Supplementary file 1 — Supplementary Tables 1–5, Fig. 1, Data 1 and 2, study protocol, statistical analysis plan and CONSORT checklist. [file 41591_2026_4465_MOESM1_ESM.pdf]

nature medicine

Supplementary information

https://doi.org/10.1038/s41591-026-04465-9

Efficacy and target engagement of dopamine agonist pramipexole for anhedonic depression: a randomized placebo-controlled trial

In the format provided by the authors and unedited

Supplementary Table 1. Adverse events in the safety population, i.e. all patients that were randomized to either pramipexole or placebo (n=85); and in the extended phase (n=54, table below). The adverse events were documented based on patient reports during conversations with study staff, while the symptoms listed in Supplementary Table 2 are based on self-ratings at each study visit. As a result, there might be discrepancies between the tables regarding impulse control-related symptoms.

PRIME-PRAXOL (RCT)

**Adverse event**	**Pramipexole**<br>**N= 43**	**Placebo**<br>**N = 42**
Any adverse event	43	40
Any serious adverse event	0	0
**CNS**		
Headache	21	18
Dizziness	14	2
Fatigue	20	13
Anxiety	13	3
Restlessness	6	2
Increased irritability	4	4
Sleep disturbances	31	14
Restless legs	2	3
Depressed mood	1	5
Cognitive symptoms	2	2
Tremor	1	1
Nightmares	1	7
Stress	0	1
Asthenia	1	2
Hallucinations	0	1
Migraine	1	2
Obsessions	1	0
Suicidal thoughts	0	1
Derealization	3	1
Hypomanic symptoms	2	0
Mood swings	1	0
Balance disturbance	1	0
Enhanced sense of taste and hearing	1	0
Increased alcohol consumption	1	0
Increased nicotine cravings	0	1
Increased thoughts about sex/buying	1(sex)	1(buying)
**Gastrointestinal/metabolic/nutritional**		
Nausea	26	6
Loss of appetite	9	2
Increased appetite	2	3
Weight loss	3	0

Weight gain	1	0
Obstipation	9	3
Gastritis	4	4
Excessive gas or flatulence	1	0
Diarrhea	3	2
Gastroenteritis	1	1
Abdominal pain	5	0
Vomiting	2	0
**Infection/inflammation**		
Rhinopharyngitis	7	7
Pharyngitis	2	1
Gingivitis	0	1
Influenza	0	2
Fever	2	1
Pneumonia	1	0
Bronchitis	1	0
UTI	2	0
Covid 19	1	1
Seasonal allergy	1	1
**Otorhinolaryngology**		
Blocked ears	1	1
Dysphagia	1	0
Sinus pain	1	0
Rhinorrhoea	1	1
Dry mouth	2	0
Tinnitus	0	2
Cough	0	2
Hoarseness	1	0
**Ophthalmology**		
Blurry vision	1	2
Dilated pupils	1	0
**Musculoskeletal system**		
Arthralgia	1	2
Muscle tension	1	3
Back pain	1	2
Muscle weakness	1	1
Whiplash	1	0
**Circulation/hematology/respiratory**		
Hypotension	1	0
Hematoma	0	1
Flashes	2	3
Thrombosis	0	1
Dyspnoea	0	2
**Urogenital**		
Ejaculation problem	1	0
Urgency	1	0

Pruritus	1	0
**Lab**		
Blood samples out of range liver	0	1
Blood samples out of range kidney	0	1
**Dermatology**		
Urticaria	1	0
Allergic reaction	1	0
Pruritus	1	0
Acne	1	0

LONG-PRAXOL (Extended Phase)

Number of patients with any AE: 46

Number of patients with SAE: 2

**CNS**	
Anxiety	10
Balance disturbance	1
Blocked ears	1
Brain fog	1
Cognitive symptoms	1
Depressed mood	2
Derealization	1
Dizziness	8
Enhanced sense of taste and hearing	1
Fatigue	10
Acute daytime sleepiness	3
Headache	9
Hiccups	1
Hypomanic symptoms	3
Increased irritability	5
Increased thoughts about buying	3
Increased thoughts about sex	2
Mood swings	2
Other	2
Paresthesia	2
Restlessness	9
Sleep disturbances	27
Suicidal thoughts	1
Tremor	1
**Gastrointestinal/metabolic/nutritional**	
Diarrhoea	1
Excessive gas or flatulence	2
Gastroenteritis	1
Increased appetite	8
Loss of appetite	9

Nausea	9
Obstipation	8
Weight loss	2
**Infection/Inflammation**	
Fever	2
Influenza	2
Pharyngitis	2
Pneuomonia	1
Rhinopharyngitis	6
Shingles	1
**Circulation/hematology/respiratory**	
Dyspnoea	1
Flashes	1
Hypertension	1
Hypotension	3
Myocardial infarction	1
Oedema	3
Palpitations	2
**Musculoskeletal system**	
Arthralgia	2
Back pain	3
Muscle tension	1
Myalgia	1
Sprain	1
Stiffness	1
Trochanteric bursitis	1
**Ophthalmology**	
Asthenopia	1
Blurry vision	2
Dilated pupils	1
Increased intraocular pressure	1
Visual disturbances	3
**Dermatology**	
Acne	1
Allodynia	1
Increased hair loss	1
Urticaria	2
**Urogenital**	
Ejaculation problem	1
**Otorhinolaryngology**	
Dry mouth	1

Supplementary Table 2. M-YMRS, M-PGSI, and M-QUIP. Prevalence (%) of items with scores >1. All scales were modified to a uniform Likert scale ranging from 0 to 3. P-values are based on chi-square tests. If patients self-reported any such symptoms, they were further evaluated by the study physician for clinical significance. Table S2A-C contains symptom ratings of the RCT. Table S2D-F contains symptom ratings of the Extended Phase.

Table S2A. Prevalence of positive responses on modified items from the Young Mania Rating Scale at each time point during the RCT in the active treatment and placebo groups. Values are presented as n/N (%). Responses are based on ratings from the past 3 days. "–" indicates no statistical test performed.

YMRS item	Week	Active treatment, n/N (%)	Placebo, n/N (%)	p-value
Elevated mood	3	24/43 (55.81%)	18/42 (42.86%)	0.28
Increased motor activity	3	15/43 (34.88%)	7/42 (16.67%)	0.08
Reduced sleep	3	26/43 (60.46%)	14/42 (33.34%)	**0.02**
Irritability	3	20/43 (46.51%)	16/42 (38.10%)	0.51
Speech (rate and amount)	3	10/43 (23.26%)	1/42 (2.38%)	**0.01**
Language–thought disorder	3	13/43 (30.23%)	6/42 (14.29%)	0.12
Appearance	3	5/43 (11.63%)	0/42 (0.00%)	0.06
Elevated mood	6	24/43 (55.81%)	16/42 (38.10%)	0.13
Increased motor activity	6	20/43 (46.51%)	5/42 (11.90%)	**0.00**
Reduced sleep	6	26/43 (60.46%)	17/42 (40.48%)	0.12
Irritability	6	17/43 (39.53%)	14/42 (33.34%)	0.65
Speech (rate and amount)	6	9/43 (20.93%)	6/42 (14.29%)	0.57
Language–thought disorder	6	10/43 (23.26%)	8/42 (19.05%)	0.79
Appearance	6	7/43 (16.28%)	4/42 (9.52%)	0.52
Elevated mood	9	23/43 (53.49%)	16/42 (38.10%)	0.27
Increased motor activity	9	14/43 (32.59%)	7/42 (16.67%)	0.13
Reduced sleep	9	28/43 (65.12%)	14/42 (33.34%)	**0.01**
Irritability	9	7/43 (16.28%)	12/42 (28.57%)	0.19
Speech (rate and amount)	9	6/43 (13.95%)	3/42 (7.14%)	0.48
Language–thought disorder	9	10/43 (23.26%)	6/42 (14.29%)	0.41
Appearance	9	5/43 (11.63%)	3/42 (7.14%)	0.71

Table S2B. Prevalence of positive responses on modified items from the Problem Gambling Severity Index at each time point during the RCT in the active treatment and placebo groups. Values are presented as n/N (%). Responses are for the past 12 months. "–" indicates no statistical test performed due to zero counts.

PGSI item	Week	Active treatment, n/N (%)	Placebo, n/N (%)	p-value
Have you bet more than you could really afford to lose?	3	0/42 (0.00%)	0/41 (0.00%)	–
Have you needed to gamble with larger amounts of money to get the same feeling of excitement?	3	0/42 (0.00%)	0/41 (0.00%)	–
Have you felt that you might have a problem with gambling?	3	0/42 (0.00%)	0/41 (0.00%)	–
Have people criticized your betting or told you that you had a gambling problem, regardless of whether or not you thought it was true?	3	0/42 (0.00%)	0/41 (0.00%)	–
Have you felt guilty about the way you gamble or what happens when you gamble?	3	0/42 (0.00%)	0/41 (0.00%)	–
Have you bet more than you could really afford to lose?	6	0/42 (0.00%)	0/40 (0.00%)	–
Have you needed to gamble with larger amounts of money to get the same feeling of excitement?	6	0/42 (0.00%)	0/40 (0.00%)	–
Have you felt that you might have a problem with gambling?	6	0/42 (0.00%)	0/40 (0.00%)	–
Have people criticized your betting or told you that you had a gambling problem, regardless of whether or not you thought it was true?	6	0/42 (0.00%)	0/40 (0.00%)	–
Have you felt guilty about the way you gamble or what happens when you gamble?	6	1/42 (2.38%)	0/40 (0.00%)	1.00
Have you bet more than you could really afford to lose?	9	0/42 (0.00%)	1/39 (2.56%)	0.48
Have you needed to gamble with larger amounts of money to get the same feeling of excitement?	9	0/42 (0.00%)	1/39 (2.56%)	0.48
Have you felt that you might have a problem with gambling?	9	0/42 (0.00%)	0/39 (0.00%)	–
Have people criticized your betting or told you that you had a gambling problem, regardless of whether or not you thought it was true?	9	0/42 (0.00%)	0/39 (0.00%)	–
Have you felt guilty about the way you gamble or what happens when you gamble?	9	0/42 (0.00%)	2/39 (5.12%)	0.23

Table S2C. Prevalence of positive responses on modified items from the Questionnaire for Impulsive-Compulsive Disorders in Parkinson's Disease at each time point during the RCT in the active treatment and placebo groups. Values are presented as n/N (%).

**Activity**	**Week**	**Question stem**	**Active treatment, n/N (%)**	**Placebo, n/N (%)**	**p-value**
Buying	3	Strong urge	8/43 (18.60%)	3/42 (7.14%)	0.19
Eating	3	Strong urge	8/43 (18.60%)	9/42 (21.43%)	0.96
Gambling	3	Strong urge	1/43 (2.33%)	0/42 (0%)	1.00
Hobbyism	3	Strong urge	12/43 (27.90%)	14/42 (33.33%)	0.76
Punding	3	Strong urge	6/43 (13.95%)	11/42 (26.19%)	0.25
Sex	3	Strong urge	6/43 (13.95%)	3/42 (7.14%)	0.48
Buying	3	Difficulty controlling	11/43 (25.58%)	2/42 (4.76%)	**0.01**
Eating	3	Difficulty controlling	9/43 (20.93%)	7/42 (16.67%)	0.82
Gambling	3	Difficulty controlling	0/43 (0.00%)	0/42 (0.00%)	1.00
Hobbyism	3	Difficulty controlling	7/43 (16.28%)	5/42 (11.90%)	0.79
Punding	3	Difficulty controlling	6/43 (13.95%)	7/42 (16.67%)	0.96
Sex	3	Difficulty controlling	1/43 (2.33%)	1/42 (2.38%)	1.00
Buying	6	Strong urge	6/43 (13.95%)	1/42 (2.38%)	0.11
Eating	6	Strong urge	8/43 (18.60%)	8/42 (19.05%)	1.00
Gambling	6	Strong urge	0/43 (0.00%)	0/42 (0.00%)	0.91
Hobbyism	6	Strong urge	7/43 (16.28%)	7/42 (16.67%)	1.00
Punding	6	Strong urge	3/43 (6.98%)	11/42 (26.19%)	**0.02**
Sex	6	Strong urge	6/43 (13.95%)	5/42 (11.90%)	1.00
Buying	6	Difficulty controlling	3/43 (6.98%)	2/42 (4.76%)	1.00
Eating	6	Difficulty controlling	6/43 (13.95%)	8/42 (19.05%)	0.69
Gambling	6	Difficulty controlling	0/43 (0.00%)	0/42 (0.00%)	0.91
Hobbyism	6	Difficulty controlling	4/43 (9.30%)	6/42 (14.29%)	0.51
Punding	6	Difficulty controlling	1/43 (2.33%)	5/42 (11.90%)	0.10
Sex	6	Difficulty controlling	1/43 (2.33%)	1/42 (2.38%)	1.00
Buying	9	Strong urge	4/43 (9.30%)	4/42 (9.52%)	1.00
Eating	9	Strong urge	4/43 (9.30%)	7/42 (16.67%)	0.34
Gambling	9	Strong urge	0/43 (0.00%)	0/42 (0.00%)	0.82
Hobbyism	9	Strong urge	9/42 (20.93%)	7/42 (16.67%)	0.91
Punding	9	Strong urge	6/43 (13.95%)	6/42 (14.29%)	1.00
Sex	9	Strong urge	3/43 (6.98%)	7/42 (16.67%)	0.18
Buying	9	Difficulty controlling	3/43 (6.98%)	3/42 (7.14%)	1.00
Eating	9	Difficulty controlling	3/43 (6.98%)	8/42 (19.05%)	0.11
Gambling	9	Difficulty controlling	0/43 (0.00%)	0/42 (0.00%)	0.82
Hobbyism	9	Difficulty controlling	6/43 (13.95%)	4/42 (9.52%)	0.74
Punding	9	Difficulty controlling	5/43 (11.63%)	5/42 (11.90%)	1.00
Sex	9	Difficulty controlling	1/43 (2.33%)	2/42 (4.76%)	0.61

Table S2D. Items from the Young Mania Rating Scale were modified to allow self-rating of manic symptoms during the extended open-label phase. Below, we report the number of patients with a score ≤1 (on a 0–3 scale) relative to the total number of patients assessed at each time point.

item	1 month	2 months	3 months	4 months	5 months	6 months
Increased motor activity	14/54	14/52	11/48	11/43	11/40	10/37
Irritability	16/54	17/52	13/48	15/43	10/40	8/37
Language–thought disorder	11/54	11/52	8/48	12/43	7/40	7/37
Reduced sleep	31/54	35/52	28/48	32/43	25/40	24/37
Speech (rate and amount)	8/54	8/52	5/48	6/43	4/40	7/37
Appearance	7/54	7/52	5/48	8/43	4/40	4/37
Elevated mood	27/54	26/52	19/48	17/43	14/40	15/37

Table S2E. Items from the Problem Gambling Severity Index were modified to allow self-rating of gambling problems during the extended open-label phase. Below, we report the number of patients with a score ≤1 (on a 0–3 scale) relative to the total number of patients assessed at each time point.

item	1 month	2 months	3 months	4 months	5 months	6 months
Have you bet more than you could really afford to lose?	0/53	0/52	0/48	0/43	0/40	0/37
Have you felt guilty about the way you gamble or what happens when you gamble?	0/53	0/52	0/48	0/43	0/40	0/37
Have you felt that you might have a problem with gambling?	0/53	0/52	0/48	0/43	0/40	0/37
Have you needed to gamble with larger amounts of money to get the same feeling of excitement?	0/53	0/52	0/48	0/43	0/40	0/37

Table S2F. Items from the Questionnaire for Impulsive–Compulsive Disorders in Parkinson's Disease were modified to allow self-rating of impulse control related symptoms during the extended open-label phase. Below, we report the number of patients with a score ≤1 (on a 0–3 scale) relative to the total number of patients assessed at each time point.

item	1 month	2 months	3 months	4 months	5 months	6 months
Buying – Strong urge	9/53	4/52	6/48	2/43	6/40	6/37
Eating – Strong urge	11/53	7/52	9/48	8/43	9/40	8/37
Sex – Strong urge	5/53	5/52	4/48	3/43	7/40	3/37
Gambling – Strong urge	0/53	0/52	1/48	0/43	0/40	0/37
Buying – Difficulty controlling	3/54	5/52	5/48	4/43	6/40	7/37
Eating – Difficulty controlling	13/53	14/52	12/48	9/43	9/40	11/37
Sex – Difficulty controlling	3/53	2/52	3/48	2/43	3/40	1/37
Gambling – Difficulty controlling	0/53	0/52	0/48	0/43	0/40	0/37

Supplementary Table 3. The PRT and cognitive tests pre- and post RCT phase.

	Pramipexole		Placebo		
	**Baseline**	**Week 9**	**Baseline**	**Week 9**	**p-value[1]**
**PRT**					
N	18	18	16	16	
**RB Block 1**					0.65
Mean	0.04	0.12	0.11	0.15	
SD	0.26	0.22	0.17	0.23	
**RB Block 2**					0.26
Mean	0.07	0.21	0.14	0.12	
SD	0.20	0.28	0.14	0.22	
**RB Block 3**					0.37
Mean	0.06	0.16	0.19	0.24	
SD	0.29	0.22	0.22	0.27	
**RBANS[2]**					
N	25	25	23	23	
**Total Score**					0.96
Mean	85.04	85.60	87.96	85.48	
SD	11.62	13.49	16.50	10.74	
**Immediate Memory**					0.49
Mean	100.56	91.88	99.43	95.57	
SD	16.41	16.40	21.03	19.78	
**Visuospatial**					0.96
Mean	73.64	72.28	73.17	72.39	
SD	7.04	9.14	8.09	8.83	
**Language**					0.21
Mean	99.68	105.00	103.26	99.22	
SD	17.70	15.37	16.91	12.97	
**Attention**					0.81
Mean	96.56	101.64	102.48	102.65	
SD	14.16	16.41	13.89	13.27	
**Delayed Memory**					0.89
Mean	81.32	80.92	81.35	81.48	
SD	12.98	13.74	17.77	10.33	
**D-KEFS CWIT**					
N	25	25	23	23	
**Inhibition**					0.41
Mean	26.28	19.76	24.30	22.13	
SD	10.18	6.67	11.51	10.70	
**Inhibition/Switching**					0.90
Mean	6.68	2.00	5.00	1.65	
SD	10.82	10.36	9.25	8.22	
**Switching**					0.27
Mean	5.52	5.84	4.17	3.04	
SD	9.03	7.81	9.22	8.84	

1 MMRM with LS-mean contrasts at endpoint.

2 Index scores.

Lower scores on D-KEFS CWIT indicate better cognitive performance

RB=Response Bias

PRT= Probabilistic Reward Task

Repeatable Battery for the Assessment of Neuropsychological Status=RBANS

Color-Word Interference Test=CWIT

Delis–Kaplan Executive Function System D-KEFS

Supplementary table 4. Plasma and cerebrospinal fluid (CSF) biomarkers pre/post RCT phase. Mean values are presented for all available samples at baseline and week 9, whereas Wilcoxon signed-rank tests were performed only in participants with complete paired samples at both time points (CRP: n=39 pramipexole, n=37 placebo; plasma DOPAC: n=34 pramipexole, n=31 placebo; plasma HVA: n=40 pramipexole, n=39 placebo; CSF DOPAC: n=10 pramipexole, n=10 placebo; CSF HVA: n=10 pramipexole, n=11 placebo).

	**Pramipexole**			**Placebo**		
	**Baseline**	**Week 9**	**P-value[1]**	**Baseline**	**Week 9**	**P-value[1]**
**CRP (plasma)**			0.01			0.46
N	40	40		38	40	
Mean (mg/L)	7.12	3.23		5.84	5.57	
SD	13.09	6.44		10.66	10.35	
**DOPAC (plasma)**			0.80			0.18
N	38	35		37	35	
Mean (ng/mL)	114.88	93.27		91.24	68.48	
SD	221.76	175.98		132.96	136.39	
**HVA (plasma)**			0.84			0.10
N	41	40		40	40	
Mean (ng/mL)	205.31	237.37		170.95	187.50	
SD	543.66	429.64		234.49	269.15	
**DOPAC (CSF)**			0.80			0.96
N	12	10		11	11	
Mean (ng/mL)	6.13	6.34		6.40	6.40	
SD	1.31	0.79		0.97	1.17	
**HVA (CSF)**			0.72			0.48
N	12	10		12	11	
Mean (ng/mL)	172.95	164.85		202.20	230.14	
SD	93.28	110.73		108.29	96.98	

1 Wilcoxon signed-rank test

CRP= C-reactive protein

DOPAC= 3,4-dihydroxyphenylacetic acid

HVA=homovanillic acid

Supplementary table 5. Dose titration schedule in RCT

Week	Step	Total dose (mg base)	Total dose (mg salt)
1	1	0.26	0.375
2	2	0.52	0.75
3	3	1.05	1.5
4	4	1.57	2.25
5	5	2.1	3.0
6	6	2.62	3.75
7	7	3.15	4.5
8		3.15	4.5
9		3.15	4.5

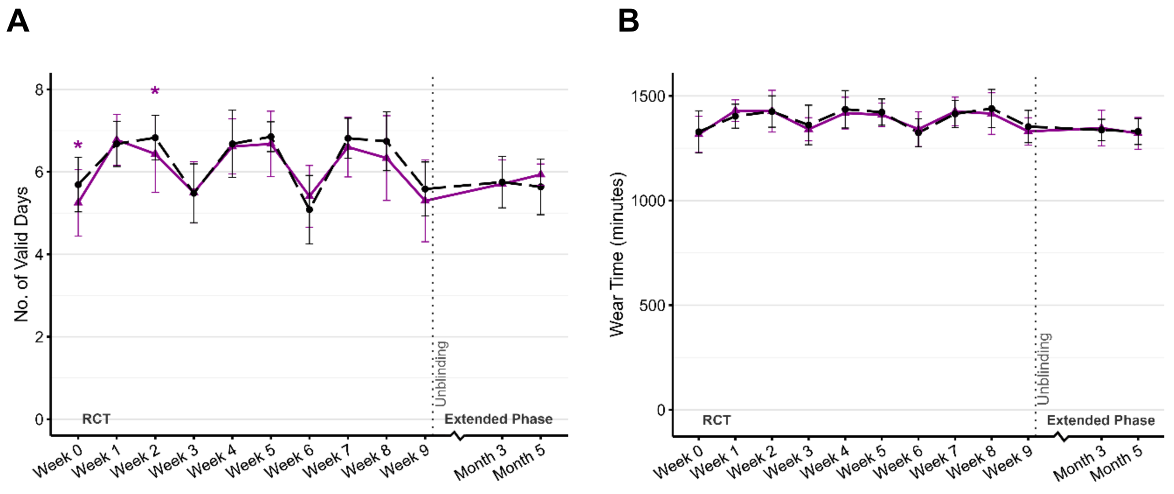

Supplementary Fig. 1 | Validation analysis of accelerometer data.

Physical activity data only includes participants with a valid baseline measurement (week 0; *n* = 61). During the RCT, participants received either pramipexole (purple solid line) or placebo (black dashed line). Following the start of the intervention, participants wore accelerometers during three distinct measurement periods of the RCT: weeks 1–3, weeks 4–6, and weeks 7–9. Each period was separated by a follow-up clinic visit for data collection. Participants (n=29) with a valid baseline measurement that agreed to participate in the extension phase received pramipexole and were instructed to wear the accelerometer for seven consecutive days following month 3 and 5 visits. Data from an individual was considered missing during the weeks not reaching the validity threshold of at least four valid days. After unblinding, the x axis time scale changes from weeks to months; month labels indicate time since unblinding, not time since RCT initiation. **A**, Mean number of valid measurement days per week for each treatment group (± 1 SD). **B**, Mean weekly wear time during the full 24-hour period (wake-up to wake-up the next day), calculated across all valid days for each treatment group (± 1 SD). Analysis revealed a consistent reduction in valid wear time during the final week of each measurement period in the RCT across both treatment groups. Pairwise contrasts conducted on RCT data between the final week and either the first or second week of the same period revealed statistically significant reductions, with p-values ranging from < 0.05 to < 0.001. No significant differences in average wear time were observed between the groups. However, the number of valid days differed significantly between the groups during two time points ($p < 0.05$).

Suppl Data S1

```
argument,"value","context"
config_file_in_outputdir,"","not applicable"
datadir,"","not applicable"
do.report,"c(2,4,5)","not applicable"
f0,"1","not applicable"
f1,"72","not applicable"
LC_TIME_backup,"Swedish_Sweden.utf8","not applicable"
mode,"c(1,2,3,4,5)","not applicable"
outputdir,"","not applicable"
studyname,"c()","not applicable"
GGIRread_version,"1.0.4","not applicable"
GGIRversion,"3.2.6","not applicable"
R_version,"R version 4.5.0 (2025-04-11 ucrt)","not applicable"
qwindow,"c(0,24)","params_247"
qlevels,"c()","params_247"
qwindow_dateformat,"%d-%m-%Y","params_247"
ilevels,"c()","params_247"
IVIS_windowsize_minutes,"60","params_247"
IVIS_epochsize_seconds,"c()","params_247"
IVIS.activity.metric,"2","params_247"
IVIS_acc_threshold,"20","params_247"
qM5L5,"c()","params_247"
MX.ig.min.dur,"10","params_247"
M5L5res,"10","params_247"
winhr,"5","params_247"
iglevels,"c()","params_247"
LUXthresholds,"c(0,100,500,1000,3000,5000,10000)","params_247"
LUX_cal_constant,"c()","params_247"
LUX_cal_exponent,"c()","params_247"
LUX_day_segments,"c()","params_247"
L5M5window,"c(0,24)","params_247"
cosinor,"FALSE","params_247"
part6CR,"FALSE","params_247"
part6HCA,"FALSE","params_247"
part6Window,"c(start,end)","params_247"
part6DFA,"FALSE","params_247"
clevels,"c(30,150)","params_247"
SRI2_WASOmin,"30","params_247"
includedaycrit,"16","params_cleaning"
ndayswindow,"7","params_cleaning"
strategy,"2","params_cleaning"
data_masking_strategy,"2","params_cleaning"
maxdur,"21","params_cleaning"
hrs.del.start,"0","params_cleaning"
hrs.del.end,"0","params_cleaning"
includedaycrit.part5,"0.666666666666667","params_cleaning"
excludefirstlast.part5,"FALSE","params_cleaning"
TimeSegments2ZeroFile,"c()","params_cleaning"
do.imp,"TRUE","params_cleaning"
```

data_cleaning_file,"c()","params_cleaning"
minimum_MM_length.part5,"23","params_cleaning"
excludefirstlast,"FALSE","params_cleaning"
includenightcrit,"16","params_cleaning"
excludefirst.part4,"FALSE","params_cleaning"
excludelast.part4,"FALSE","params_cleaning"
max_calendar_days,"0","params_cleaning"
nonWearEdgeCorrection,"TRUE","params_cleaning"
nonwear_approach,"2023","params_cleaning"
segmentWEARcrit.part5,"0.5","params_cleaning"
segmentDAYSPTcrit.part5,"c(0.9,0)","params_cleaning"
study_dates_file,"c()","params_cleaning"
study_dates_dateformat,"%d-%m-%Y","params_cleaning"
includecrit.part6,"c(0.666666666666667,0.666666666666667)","params_cleaning"
includenightcrit.part5,"0","params_cleaning"
nonwearFiltermaxHours,"c()","params_cleaning"
nonwearFilterWindow,"c()","params_cleaning"
overwrite,"FALSE","params_general"
acc.metric,"ENMO","params_general"
maxNcores,"c()","params_general"
print.filename,"TRUE","params_general"
do.parallel,"TRUE","params_general"
windowsizes,"c(1,900,3600)","params_general"
desiredtz,"Europe/Stockholm","params_general"
configtz,"c()","params_general"
idloc,"5","params_general"
dayborder,"0","params_general"
part5_agg2_60seconds,"FALSE","params_general"
sensor.location,"wrist","params_general"
expand_tail_max_hours,"c()","params_general"
recordingEndSleepHour,"c()","params_general"
dataFormat,"raw","params_general"
maxRecordingInterval,"c()","params_general"
extEpochData_timeformat,"%d-%m-%Y %H:%M:%S","params_general"
recording_split_times,"c()","params_general"
recording_split_timeformat,"%d/%m/%Y %H:%M","params_general"
recording_split_overlap,"0","params_general"
recording_split_ignore_edges,"FALSE","params_general"
do.anglex,"FALSE","params_metrics"
do.angley,"FALSE","params_metrics"
do.anglez,"TRUE","params_metrics"
do.zcx,"FALSE","params_metrics"
do.zcy,"FALSE","params_metrics"
do.zcz,"FALSE","params_metrics"
do.enmo,"TRUE","params_metrics"
do.lfenmo,"FALSE","params_metrics"
do.en,"FALSE","params_metrics"
do.mad,"FALSE","params_metrics"
do.enmoa,"FALSE","params_metrics"
do.roll_med_acc_x,"FALSE","params_metrics"

do.roll_med_acc_y,"FALSE","params_metrics"
do.roll_med_acc_z,"FALSE","params_metrics"
do.dev_roll_med_acc_x,"FALSE","params_metrics"
do.dev_roll_med_acc_y,"FALSE","params_metrics"
do.dev_roll_med_acc_z,"FALSE","params_metrics"
do.bfen,"FALSE","params_metrics"
do.hfen,"FALSE","params_metrics"
do.hfenplus,"FALSE","params_metrics"
do.lfen,"FALSE","params_metrics"
do.lfx,"FALSE","params_metrics"
do.lfy,"FALSE","params_metrics"
do.lfz,"FALSE","params_metrics"
do.hfx,"FALSE","params_metrics"
do.hfy,"FALSE","params_metrics"
do.hfz,"FALSE","params_metrics"
do.bfx,"FALSE","params_metrics"
do.bfy,"FALSE","params_metrics"
do.bfz,"FALSE","params_metrics"
do.brondcounts,"FALSE","params_metrics"
do.neishabouricounts,"FALSE","params_metrics"
hb,"15","params_metrics"
lb,"0.2","params_metrics"
n,"4","params_metrics"
zc.lb,"0.25","params_metrics"
zc.hb,"3","params_metrics"
zc.sb,"0.01","params_metrics"
zc.order,"2","params_metrics"
zc.scale,"1","params_metrics"
actilife_LFE,"FALSE","params_metrics"
epochvalues2csv,"FALSE","params_output"
save_ms5rawlevels,"TRUE","params_output"
save_ms5raw_format,"RData","params_output"
save_ms5raw_without_invalid,"FALSE","params_output"
storefolderstructure,"FALSE","params_output"
timewindow,"c(WW,MM)","params_output"
viewingwindow,"1","params_output"
dofirstpage,"TRUE","params_output"
visualreport,"FALSE","params_output"
week_weekend_aggregate.part5,"FALSE","params_output"
do.part3.pdf,"FALSE","params_output"
outliers.only,"FALSE","params_output"
criterror,"3","params_output"
do.visual,"TRUE","params_output"
do.sibreport,"TRUE","params_output"
do.part2.pdf,"TRUE","params_output"
sep_reports,",","params_output"
sep_config,",","params_output"
dec_reports,".","params_output"
dec_config,".","params_output"
visualreport_without_invalid,"TRUE","params_output"

old_visualreport,"FALSE","params_output"
visualreport_hrsPerRow,"36","params_output"
visualreport_focus,"day","params_output"
visualreport_validcrit,"0","params_output"
require_complete_lastnight_part5,"FALSE","params_output"
method_research_vars,"c()","params_output"
mvpathreshold,"100.6","params_phyact"
boutcriter,"0.8","params_phyact"
mvpadur,"c(1,5,10)","params_phyact"
boutcriter.in,"0.9","params_phyact"
boutcriter.lig,"0.8","params_phyact"
boutcriter.mvpa,"0.8","params_phyact"
threshold.lig,"44.8","params_phyact"
threshold.mod,"100.6","params_phyact"
threshold.vig,"428.8","params_phyact"
boutdur.mvpa,"c(1,5,10)","params_phyact"
boutdur.in,"c(10,20,30)","params_phyact"
boutdur.lig,"c(1,5,10)","params_phyact"
frag.metrics,"c()","params_phyact"
part6_threshold_combi,"44.8_100.6_428.8","params_phyact"
chunksize,"1","params_rawdata"
spherecrit,"0.3","params_rawdata"
minloadcrit,"168","params_rawdata"
printsummary,"TRUE","params_rawdata"
do.cal,"TRUE","params_rawdata"
backup.cal.coef,"retrieve","params_rawdata"
dynrange,"c()","params_rawdata"
minimumFileSizeMB,"2","params_rawdata"
rmc.dec,".","params_rawdata"
rmc.firstrow.acc,"c()","params_rawdata"
rmc.firstrow.header,"c()","params_rawdata"
rmc.header.length,"c()","params_rawdata"
rmc.col.acc,"c(1,2,3)","params_rawdata"
rmc.col.temp,"c()","params_rawdata"
rmc.col.time,"c()","params_rawdata"
rmc.unit.acc,"g","params_rawdata"
rmc.unit.temp,"C","params_rawdata"
rmc.unit.time,"POSIX","params_rawdata"
rmc.format.time,"%Y-%m-%d %H:%M:%OS","params_rawdata"
rmc.bitrate,"c()","params_rawdata"
rmc.dynamic_range,"c()","params_rawdata"
rmc.unsignedbit,"TRUE","params_rawdata"
rmc.origin,"1970-01-01","params_rawdata"
rmc.desiredtz,"c()","params_rawdata"
rmc.configtz,"c()","params_rawdata"
rmc.sf,"c()","params_rawdata"
rmc.headername.sf,"c()","params_rawdata"
rmc.headername.sn,"c()","params_rawdata"
rmc.headername.recordingid,"c()","params_rawdata"
rmc.header.structure,"c()","params_rawdata"

rmc.check4timegaps,"FALSE","params_rawdata"
rmc.noise,"13","params_rawdata"
nonwear_range_threshold,"150","params_rawdata"
rmc.col.wear,"c()","params_rawdata"
rmc.doresample,"FALSE","params_rawdata"
interpolationType,"1","params_rawdata"
imputeTimegaps,"TRUE","params_rawdata"
frequency_tol,"0.1","params_rawdata"
rmc.scalefactor.acc,"1","params_rawdata"
anglethreshold,"5","params_sleep"
timethreshold,"5","params_sleep"
ignorenonwear,"TRUE","params_sleep"
HASPT.algo,"HDCZA","params_sleep"
HASIB.algo,"vanHees2015","params_sleep"
Sadeh_axis,NA,"params_sleep"
longitudinal_axis,"c()","params_sleep"
HASPT.ignore.invalid,"FALSE","params_sleep"
loglocation,"c()","params_sleep"
colid,"1","params_sleep"
coln1,"2","params_sleep"
nnights,"c()","params_sleep"
relyonguider,"FALSE","params_sleep"
def.noc.sleep,"1","params_sleep"
sleeplogsep,"c()","params_sleep"
sleepwindowType,"SPT","params_sleep"
possible_nap_window,"c()","params_sleep"
possible_nap_dur,"c()","params_sleep"
possible_nap_gap,"0","params_sleep"
possible_nap_edge_acc,"Inf","params_sleep"
nap_model,"c()","params_sleep"
sleepefficiency.metric,"1","params_sleep"
HDCZA_threshold,"c()","params_sleep"
oakley_threshold,"20","params_sleep"
consider_marker_button,"FALSE","params_sleep"
impute_marker_button,"FALSE","params_sleep"
sib_must_fully_overlap_with_TimeInBed,"c(TRUE,TRUE)","params_sleep"
nap_markerbutton_method,"0","params_sleep"

Suppl Data S2

Anatomical data preprocessing

A total of 2 T1-weighted (T1w) images were found within the input BIDS dataset. All of them were corrected for intensity non-uniformity (INU) with N4BiasFieldCorrection (Tustison et al. 2010), distributed with ANTs (version unknown) (Avants et al. 2008, RRID:SCR_004757). The T1w-reference was then skull-stripped with a Nipype implementation of the antsBrainExtraction.sh workflow (from ANTs), using OASIS30ANTs as target template. Brain tissue segmentation of cerebrospinal fluid (CSF), white-matter (WM) and gray-matter (GM) was performed on the brain-extracted T1w using fast (FSL (version unknown), RRID:SCR_002823, Zhang, Brady, and Smith 2001). An anatomical T1w-reference map was computed after registration of 2 T1w images (after INU-correction) using mri_robust_template (FreeSurfer 7.3.2, Reuter, Rosas, and Fischl 2010). Volume-based spatial normalization to one standard space (MNI152NLin2009cAsym) was performed through nonlinear registration with antsRegistration (ANTs (version unknown)), using brain-extracted versions of both T1w reference and the T1w template. The following template was were selected for spatial normalization and accessed with TemplateFlow (23.0.0, Ciric et al. 2022): ICBM 152 Nonlinear Asymmetrical template version 2009c [Fonov et al. (2009), RRID:SCR_008796; TemplateFlow ID: MNI152NLin2009cAsym].

Functional data preprocessing

For each of the 2 BOLD runs found per subject (across all tasks and sessions), the following preprocessing was performed. First, a reference volume and its skull-stripped version were generated using a custom methodology of fMRIPrep. Head-motion parameters with respect to the BOLD reference (transformation matrices, and six corresponding rotation and translation parameters) are estimated before any spatiotemporal filtering using mcflirt (FSL , Jenkinson et al. 2002). The BOLD time-series (including slice-timing correction when applied) were resampled onto their original, native space by applying the transforms to correct for head-motion. These resampled BOLD time-series will be referred to as preprocessed BOLD in original space, or just preprocessed BOLD. The BOLD reference was then co-registered to the T1w reference using mri_coreg (FreeSurfer) followed by flirt (FSL , Jenkinson and Smith 2001) with the boundary-based registration (Greve and Fischl 2009) cost-function. Co-registration was configured with six degrees of freedom. Several confounding time-series were calculated based on the preprocessed BOLD: framewise displacement (FD), DVARS and three region-wise global signals. FD was computed using two formulations following Power (absolute sum of relative motions, Power et al. (2014)) and Jenkinson (relative root mean square displacement between affines, Jenkinson et al. (2002)). FD and DVARS are calculated for each functional run, both using their implementations in Nipype (following the definitions by Power et al. 2014). The BOLD time-series were resampled into standard space, generating a preprocessed BOLD run in MNI152NLin2009cAsym space. First, a reference volume and its skull-stripped version were generated using a custom methodology of fMRIPrep. All resamplings can be performed with a single interpolation step by composing all the pertinent transformations (i.e. head-motion transform matrices, susceptibility distortion correction when available, and co-registrations to anatomical and output spaces). Gridded (volumetric) resamplings were performed using antsApplyTransforms (ANTs), configured with Lanczos interpolation to minimize the smoothing effects of other kernels (Lanczos 1964). Non-gridded (surface) resamplings were performed using mri_vol2surf (FreeSurfer).

References

Abraham, Alexandre, Fabian Pedregosa, Michael Eickenberg, Philippe Gervais, Andreas Mueller, Jean Kossaifi, Alexandre Gramfort, Bertrand Thirion, and Gael Varoquaux.

2014. “Machine Learning for Neuroimaging with Scikit-Learn.” *Frontiers in Neuroinformatics* 8. https://doi.org/10.3389/fninf.2014.00014.

Avants, B. B., C. L. Epstein, M. Grossman, and J. C. Gee. 2008. “Symmetric Diffeomorphic Image Registration with Cross-Correlation: Evaluating Automated Labeling of Elderly and Neurodegenerative Brain.” *Medical Image Analysis* 12 (1): 26–41. https://doi.org/10.1016/j.media.2007.06.004.

Ciric, R., William H. Thompson, R. Lorenz, M. Goncalves, E. MacNicol, C. J. Markiewicz, Y. O. Halchenko, et al. 2022. “TemplateFlow: FAIR-Sharing of Multi-Scale, Multi-Species Brain Models.” *Nature Methods* 19: 1568–71. https://doi.org/10.1038/s41592-022-01681-2.

Fonov, VS, AC Evans, RC McKinstry, CR Almli, and DL Collins. 2009. “Unbiased Nonlinear Average Age-Appropriate Brain Templates from Birth to Adulthood.” *NeuroImage*47, Supplement 1: S102. https://doi.org/10.1016/S1053-8119(09)70884-5.

Greve, Douglas N, and Bruce Fischl. 2009. “Accurate and Robust Brain Image Alignment Using Boundary-Based Registration.” *NeuroImage* 48 (1): 63–72. https://doi.org/10.1016/j.neuroimage.2009.06.060.

Jenkinson, Mark, Peter Bannister, Michael Brady, and Stephen Smith. 2002. “Improved Optimization for the Robust and Accurate Linear Registration and Motion Correction of Brain Images.” *NeuroImage* 17 (2): 825–41. https://doi.org/10.1006/nimg.2002.1132.

Jenkinson, Mark, and Stephen Smith. 2001. “A Global Optimisation Method for Robust Affine Registration of Brain Images.” *Medical Image Analysis* 5 (2): 143–56. https://doi.org/10.1016/S1361-8415(01)00036-6.

Lanczos, C. 1964. “Evaluation of Noisy Data.” *Journal of the Society for Industrial and Applied Mathematics Series B Numerical Analysis* 1 (1): 76–85. https://doi.org/10.1137/0701007.

Power, Jonathan D., Anish Mitra, Timothy O. Laumann, Abraham Z. Snyder, Bradley L. Schlaggar, and Steven E. Petersen. 2014. “Methods to Detect, Characterize, and Remove Motion Artifact in Resting State fMRI.” *NeuroImage* 84 (Supplement C): 320–41. https://doi.org/10.1016/j.neuroimage.2013.08.048.

Reuter, Martin, Herminia Diana Rosas, and Bruce Fischl. 2010. “Highly Accurate Inverse Consistent Registration: A Robust Approach.” *NeuroImage* 53 (4): 1181–96. https://doi.org/10.1016/j.neuroimage.2010.07.020.

Tustison, N. J., B. B. Avants, P. A. Cook, Y. Zheng, A. Egan, P. A. Yushkevich, and J. C. Gee. 2010. “N4ITK: Improved N3 Bias Correction.” *IEEE Transactions on Medical Imaging* 29 (6): 1310–20. https://doi.org/10.1109/TMI.2010.2046908.

Zhang, Y., M. Brady, and S. Smith. 2001. “Segmentation of Brain MR Images Through a Hidden Markov Random Field Model and the Expectation-Maximization Algorithm.”*IEEE Transactions on Medical Imaging* 20 (1): 45–57. https://doi.org/10.1109/42.906424.

Adjuvant Treatment with Pramipexole for Anhedonia Symptoms in Depression - PRIME-PRAXOL

Clinical trial protocol

Version: 8,0

Dated: 2025- 06-19

Protocol ID: NTC05355337

EU CT number 2024-512495-35-00

Sponsor: Region Skåne, Adult Psychiatry Lund.

Principal investigator: Daniel Lindqvist.

2. Table of contents

Contents

1. Title ... 1

2. Table of contents ... 2

3. Signature page ... 7

4. Abbreviations ... 8

5. Contact information ... 13

6. Synopsis ... 14

7. CONSORT Flowchart ... 20

8. Background ... 21

9. Risk-benefit assessment ... 24

    9.1 Experiences from our pilot study in Region Skåne (proof of concept) ... 26

10. Aims and questions ... 27

    10.1 Primary question and variable ... 28

    10.2 Secondary questions and variables ... 28

11. Trial design ... 30

12. Study population ... 31

    12.1. Criteria ... 31

        12.1.1. Inclusion criteria ... 31

        12.1.2. Exclusion criteria ... 31

    12.2. Criteria for withdrawal from the trial ... 32

13. Trial implementation ... 34

13.1. Recruitment and screening visits ... 36
13.2. Process for obtaining Informed Consent ... 37
13.3. Before trial treatment – Baseline (7-14 days after screening) ... 38
13.4. Treatment allocation ... 39
13.5. Visits during treatment ... 39
13.6. Final visit ... 40
13.7. Visits in the event of premature study withdrawal ... 40
13.8. Trial Compliance ... 41
13.9. Biological sampling procedures ... 41
13.9.1. Handling, storage and destruction of biological samples ... 41
13.9.2 Total blood volume and volume of spinal fliud per research subject ... 42
13.9.3. Biobank ... 42
14. Trial drugs ... 43
14.1. Investigational medicinal products ... 43
14.2. Handling and labelling of Investigational Medicinal Products ... 44
14.2.1. Labelling of Investigational Medicinal Products ... 44
14.2.2. Traceability of Investigational Medicinal Products ... 44
14.2.3. Storage of Investigational Medicinal Products ... 44
14.3. Dosage and Drug Administration ... 44
14.4. Non-trial medicines ... 46
14.5. Concomitant medication ... 46
14.6. Randomisation ... 47
14.7. Blinding ... 47

14.8. Code breaking 488

15. Methods for collecting study variables 49

15.1. Effect variables 49

15.2. Safety and tolerability variables 49

15.2.1. Laboratory variables 49

15.2.2. Vital signs 49

15.2.3. Tolerability variables 50

16. Safety monitoring and reporting 51

16.1. Definition of an adverse event 51

16.2. Reference safety information 52

16.3. Assessment of incident severity 52

16.4. Assessment of incidentcausality 52

16.5. Incident registration 54

16.6 Pregnancy 55

16.7 Incident reporting 55

16.7.1 Reporting of adverse events (AEs) and adverse reactions (ARs) 55

16.7.2 Reporting of serious incidents and serious adverse reactions (SAEs and SARs) 55

16.7.3 Reporting of SUSARs - suspected unexpected serious adverse reactions 55

16.8 Annual safety report 56

16.9 Procedure in case of overdose or other emergency 56

17. Statistical considerations 57

17.1. Dimensional calculation 57

17.2. Statistical analysis of primary, secondary and exploratory variables 57

17.3. Interim analysis 58

18. Data management 59

18.1. Data flow and processing 59

18.2. CRF 59

18.3. Source data documents 59

18.4. Storage and archiving of study data and source documents / Access to data 60

19. Quality Control and Monitoring 61

20. Storage and archiving 62

21. Ethical and regulatory concerns 63

21.1. Major changes to the trial 63

21.2. Serious breaches 63

21.3. Reporting the end of the trial 64

21.4. Informing research subjects and obtaining consent 64

21.5 Compensation for sub-trial participation 64

21.6 Data protection 65

22. Timetable and definition of end of the trial 66

23. Insurance 67

24. Funding 67

25. Registration, reporting and publication 67

25.1. Registration 67

25.2. Reporting and publication 67

25.3. Results, preliminary and final, may be presented at national and international meetings. 68

26. References 69

27. Changes of the protocol after initial approval……………………………………………73

3. Signature page

I have read this protocol and it contains all the essential elements to conduct the trial. With my signature, I agree to conduct the trial in all its aspects according to this protocol, the informed consent, and to comply with the ICH-GCP, the Declaration of Helsinki and the national and international regulatory frameworks relevant to the current clinical trial.

I will share the protocol and any other important trial-related information with my staff so that they can conduct the trial properly. I am aware of my responsibility to keep staff working on the trial informed and trained.

I understand that any information provided to me in connection with this trial that has not been previously published is considered confidential information.

……………………………………………………………………………………………………

Signature of principal investigator Date (dd-mm-2021)

Daniel Lindqvist

Department of Psychiatry, Habilitation and Assistive Technology, Region Skåne

4. Abbreviations

7T MR	7-Tesla Magnetic Resonance Imaging
AE	Adverse event/Incident - undesirable medical event
ADR	Adverse drug reaction
AES	The Apathy Evaluation Scale
BOLD	Blood-oxygen-level dependent imaging
BBQ	Brunnsviken Brief Quality-of-life scale
CGI-S	The Clinical Global Impressions Scale
COVID-19	Coronavirus disease 2019
CPT-3	Conners Continius Auditory test of Attention
CRF	Case report form
CRP	C-reactive protein
CSF	Cerebrospinal fluid
D3 receptor	D3-type dopamine receptor
DARS/DARS-SV-MOD	Dimensional Anhedonia Rating Scale (SV-MOD stands for a modified Swedish translation)
D-KEFS	Delis-Kaplan Executive Function System
DOPA	Levodopa
DOPAC	3,4-Dihydroxyphenylacetic acid

DSF	European Union Data Protection Regulation (EU 2016/679) (GDPR)
DSM-5	Diagnostic and Statistical Manual of Mental Disorders 5 ed.
eCRF	Electronic Case Report Form
ECT	Electroconvulsive therapy
eGFR	Estimated glomerular filtration rate
EMA	European Medicines Agency
EIPS	Emotionally Unstable Personality Disorder
EU	European Union
EU CTR	EU Clinical Trials Register
FASS	Farmaceutiska Specialiteter I Sverige
fMRI	Functional magnetic resonance imaging
GAD-7	Generalized Anxiety Disorder 7-item scale
GCP	Good clinical practice
GMP	Good manufacturing practice
Hb	Haemoglobin
HDRS-17	Hamilton Rating Scale for Depression, 17 items
HDRS-6	Hamilton Rating Scale for Depression, 6 items
HVA	*Homovanillic acid*

ICD-10	International Statistical Classification of Diseases and Related Health Problems 10 ed.
ICH	International Council for Harmonisation
ICMJE	International Committee of Medical Journal Editors
IL	Interleukin
ISF	*Investigator Site File*
ISI	Insomnia Severity Index
KVB	*Kvalitetsregister, vårddatabaser och beredning*, a medical records authorisation body
Source data	All original information and certified copies of original documents of clinical findings, observations or other activities in a clinical trial that are necessary for the reconstruction and evaluation of the trial. Source data is available in source documents (original or certified copies). (ICH-GCP E6)
LOCF	last observation carried forward
LPT	The Compulsory Psychiatric Care Act
LV	The Swedish Medical Products Agency
LVFS 2011:19	Guidance to the Medical Products Agency's regulations on clinical trials of medicinal products in humans
MADRS-S	Montgomery-Åsberg Depression Rating Scale (S indicates self-evaluation)

MAO inhibitors	monoamine oxidase inhibitors
MID task	Monetary Incentive Delay task
MINI	Mini International Neuropsychiatric Interview
Monitor	An independent person who verifies that the trial is conducted, documented and reported in accordance with the approved trial protocol, Standard Operating Procedures (SOPs), Good Clinical Practice (GCP), and applicable regulatory requirements.
MTA	Methoxytyramine
NAc	The nucleus accumbens
PAL	Doctor in charge of the patient in a psychiatric ward or health centre
PoC	Proof of concept
PDQ-5	Perceived Deficits Questionnaire 5-item version
PRT	Probabilistic Reward Task
Investigator/Principal investigator	A licensed general practitioner or dentist conducting a clinical trial of a medicinal product at an investigation site.
PS	Parkinson's disease
RBANS	Repeatable Battery of the Assessment of Neuropsychological Status
EPM	The Swedish Ethical Review Authority
RCT	Randomised controlled trial

SAE	Serious Adverse Event- undesirable medical event
SD	Standard deviation
SHAPS-C	Snaith-Hamilton Pleasure Scale (C stands for *clinical*)
SmPC	Summary of Product Characteristics (SPC) - document intended for doctors/medical staff written by the manufacturer when a medicine is registered; a summary of the medicine's characteristics and use.
SSRIS	Selective serotonin reuptake inhibitors
SUAS	Suicide Assessment Scale
SUS	Skåne University Hospital
SUSAR	Suspected Unexpected Serious Adverse Reaction
TNF	Tumour necrosis factor
WAIS-IV	Weschler adult Intelligence Scale (4th edition)
QUIP	Questionnaire for Impulsive-Compulsive Disorders in Parkinson's Disease

5. Contact information

Trial role	Name and title	Contact info:
Trial sponsor and Principal Investigator	Daniel Lindqvist; Docent and Senior Lecturer at Lund University, IKVL, Psychiatry.<br><br>Psychiatric Specialist, Department of Psychiatry, Habilitation and Assistive Technology, Region Skåne.	**Address:** Forskningsenheten, Baravägen 1, 221 85 Lund<br><br>**e-mail:** daniel.lindqvist@med.lu.se<br><br>**Phone:** 046 - 173 885
Investigator	Filip Ventorp; PhD, Registered Medical Practitioner	**Address:** Vuxenpsykiatri Lund, Baravägen 1, 221 85 Lund<br><br>**e-mail:** filip.ventorp@med.lu.se<br><br>**Phone:** 046 – 174 843
Investigator	Jesper Lindahl; Doctoral student, Registered Medical Practitioner	**Address:** Vuxenpsykiatri Lund, Baravägen 1, 221 85 Lund<br><br>**e-mail:** jesper.lindahl@med.lu.se<br><br>**Phone:** 046 – 174 457
Head of Adult Psychiatry Lund (authorised representative of the research principal)	Eva-Lena Brönmark	**Address:** Vuxenpsykiatri Lund, Baravägen 1, 221 85 Lund<br><br>**e-mail:** eva-lena.bronmark@skane.se<br><br>**Phone:** 046 – 174 018
Monitor	Clinical Studies Sweden - Forum South	**Address:** Skåne University Hospital,<br><br>221 85 Lund

6. Synopsis

Title	Adjuvant Treatment with Pramipexole for Anhedonia Symptoms in Depression
Sponsor's Protocol ID	NCT05355337
EudraCT no.	EU CT-number 2024-512495-35-00<br><br>EudraCT: 2022-001563-26
Background and Expected Benefits	The majority of depressed patients do not achieve remission with current treatment methods. A common and debilitating symptom after treatment is anhedonia - the inability to feel joy and diminished motivation. At present, there is no specific and effective treatment for anhedonia. Pramipexole is a dopamine agonist used in Parkinson's disease which has been shown in several clinical trials to be effective against anhedonia symptoms in this disease. Clinical experience has shown treatment efficacy with high-dose pramipexole in therapy-refractory depression, but randomised clinical trials have only been conducted with low-dose pramipexole and have not investigated the specific effects on anhedonia. In a recently completed pilot study by our research team, about one-third of patients showed a treatment effect on high-dose pramipexole.
Target	**Primary question:** Will treatment with pramipexole at the highest possible dose (without intolerable adverse reactions, max 3.15 mg base) reduce anhedonia symptoms over a nine-week period compared to placebo treatment?

	**Secondary question 1:** Will treatment with pramipexole at the highest possible dose (without intolerable adverse reactions, max 3.15 mg base) reduce depression symptoms (Bech-6 scale) over a nine-week period?<br><br>**Secondary question 2:** Will treatment with pramipexole at the highest possible dose (without intolerable adverse reactions, max 3.15 mg base) increase daily physical activity, reduce stress, and improve sleep quality as recorded by activity meters over a nine-week period?<br><br>**Secondary question 3:** Will treatment with pramipexole at the highest possible dose (without intolerable adverse reactions, max 3.15 mg base) reduce psychological symptoms as measured by the DARS-SV-MOD, MADRS, AES, *Insomnia Severity Scale,* BBQ and GAD-7 rating scales over a nine-week period?<br><br>**Secondary question 4:** Will treatment with pramipexole at the highest possible dose (without intolerable adverse reactions, max 3.15 mg base) increase the activity of the ventral striatum in the context of the MID task at fMRI?<br><br>**Secondary question 5:** Can inflammatory and dopamine turnover markers in blood and CSF, activity and connectivity in the ventral striatum during MID task predict the treatment response of pramipexole?<br><br>**Secondary question 6:** Is **P**ramipexole a safe and tolerable treatment anhedonic depression?

	**Secondary question 7:** Is cognitive function related to state (symptom severity/anhedonia) or scar/trait (duration/vulnerability) after treatment with pramipexole?<br><br>**Secondary question 8**: Will treatment with pramipexole at the highest possible dose (without intolerable adverse events, max 3.15 base) improve PRT performance over a nine-week period?
Variable	**Primary variable:** SHAPS-C<br><br>**Secondary variable 1:** HDRS6 scores (sub-scale of HDRS-17).<br><br>**Secondary variables 2:** Number of steps per day, distribution of movement pattern over the day, walking distance, time spent in light, moderate and intense physical activity, resting heart rate, oxygen saturation in blood, heart rate variability (stress scores), sleep latency (time to fall asleep), sleep awakening (how often one wakes up during the night), wakefulness (time in minutes awake during one night), time in deep sleep, sleep efficiency (sleep time vs. total time spent in bed). Measured using activity meters.<br><br>**Secondary variables 3:** Total scores of DARS-SV-MOD, MADRS, AES, Insomnia *Severity Scale* and GAD-7.<br><br>**Secondary variable 4**: BOLD activity in the nucleus accumbens during fMRI (MID task).<br><br>**Secondary variables 5:** Biomarkers related to dopamine and inflammation, measured in blood and CSF. BOLD activity in the

	nucleus accumbens during MID task fMRI and connectivity during diffusion tensor imaging.<br><br>**Secondary variables 6**: Adverse Event<br><br>**Secondary varibles 7:** Neuropsychological test battery consisting WAIS-IV, RBANS, D-KEFS, CPT-3, and cognitive self-assessment PDQ-5.<br><br>**Secondary variables 8:** Probablistic Reward task (PRT)
Investigational medicinal product/Treatment	Pramipexole extended-release tablet, 0.26 mg base to 3.15 mg base/day for 9 weeks (individually varying dose up-titrated during these weeks) and corresponding placebo tablets.
Study population	**Inclusion (incomplete list):** Age between 18 and 75 years. Diagnosis of unipolar depressive episode, bipolar disorder in depressive phase, or dysthymia. All patients should have pronounced symptoms of anhedonia (SHAPS score 3 or 4 on ≥ 3 items). Participants must have been on stable treatment with at least one antidepressant/mood-stabilising drug for at least 4 weeks.<br><br>**Exclusion (incomplete list):** Currently pregnant, breastfeeding or planned pregnancy. High suicide risk. Substance abuse. Mental illness. EIPS. Treatment under LPT. History of impulse-control disorder. Diagnosis of moderate/severe renal failure or severe cardiovascular disease. Recently started psychotherapy. Ongoing ECT treatment.
Trial design	Randomised controlled trial with two arms (pramipexole or placebo). All included research subjects will also be asked to participate

	in the sub-trial for fMRI examination and lumbar puncture(before and after 9 weeks of treatment with pramipexole)
Timetable	**Planned trial start:** 1 October 2022<br><br>**Planned trial end:** 1 December 2026.
Statistical considerations	To answer the primary research question, we use a mixed design analysis of variance (ANOVA) with severity of anhedonia symptoms (SHAPS-C total score) as the dependent variable and time (in weeks) and treatment (pramipexole or placebo) as the independent variable.
Methodological description	Recruitment of research subjects is done through various channels, such as recruitment from clinics or departments within the health care system or through advertising of various kinds (including social media, web, newspapers, radio or TV). Those who meet inclusion and no exclusion criteria at the screening visit undergo a baseline visit where assessment with self-rating scales is performed as well as blood sampling and lumbar puncture (sub-trial). Participants are also asked to submit to an fMRI examination of the brain's reward system (MID task) and a neuropsychological testing including Probabilistic Reward Task (PRT). Thereafter, trial treatment begins according to schedule with weekly dose increases until treatment effect, maximum dose, or intolerable adverse reactions occurs. In case of intolerable adverse reactions, the research subject will return to the last tolerable dose. One staff member will be unblinded and handle medications, but will neither see nor assess patients. A new attempt to increase the amount is made after seven days on a maximum of two occasions. After nine weeks of

	pramipexole treatment, a final visit is made with a new assessment using self-assessment scales and blood tests as at the baseline visit (including a new fMRI scan and lumbar puncture for sub-trial participants). After the end of the trial, patients will be taken over by a doctor not involved in the present trial. The unblinded staff member (who is in charge of randomisation) will communicate to this doctor whether the trial participant has received placebo or pramipexole. In the event that the study participant has received pramipexole, they will be offered to continue this treatment in the regular care setting.
Funding	The study has received funding from the Swedish Research Council (grant number 2020-01428), Swedish governmental funding of clinical research (ALF) (Award/Grant number is not applicable), grants from the province of Scania (Award/Grant number is not applicable), the Crafoord Foundation (grant number 20220522), the Brain Foundation (FO2022-0050 & FO2025-0058-HK-168), Ellen and Henrik Sjöbring Foundation (Award/Grant number is not applicable), Söderström – Königska Foundation (grant number SLS-969692), Region Kronoberg (Award/Grant number is not applicable), Bror Gadelius Foundation (Award/Grant number is not applicable), the Engkvist Foundation (grant number 214-0363), John Hains Foundation, and the Sten K. Johnson Foundation.

7. CONSORT Flowchart

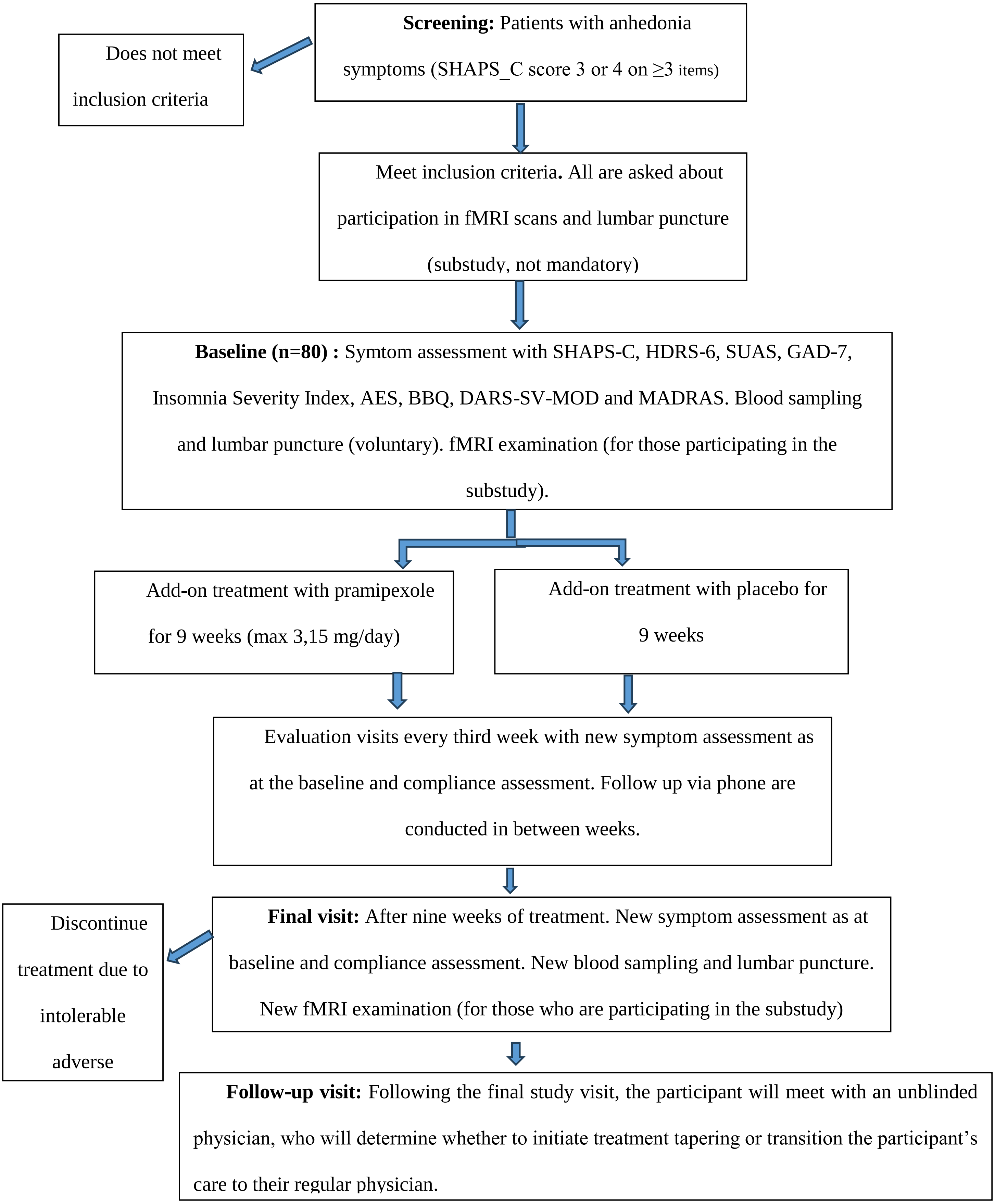

8. Background

Please note: In the scientific literature on pramipexole, the dosage of pramipexole is used in the salt form, whereas in Sweden and in FASS it is listed in the base form. In the protocol, concentration is in base unless otherwise stated.

Affective disorders are psychiatric syndromes whose main symptoms involve altered affects or moods. Examples of such disorders are major depression (unipolar depression), bipolar disorder and dysthymia. Current treatments for these conditions are often inadequate. From the start of a drug treatment, it usually takes several weeks before the treatment effect is noticeable, although possible adverse reactions often occur earlier[1]. In addition, a large proportion of patients do not respond at all to drug treatment despite multiple treatment attempts, and many of those who do respond to treatment have severe and debilitating residual symptoms such as anhedonia and lack of motivation[2].

It is estimated that up to one third of patients with depression do not achieve remission despite repeated attempts at treatment with different drugs[3]. One reason is that the diagnostic criteria in manuals and systems such as DSM-5 and ICD-10 do not take into account the biological causes of diseases. In an attempt to better study depression and other affective disorders, psychiatric syndromes can be seen as a result of different endophenotypes[4], i.e., characteristic features (leading in this case to different psychiatric symptoms) with a clear biological and genetic correlate that may occur across diagnoses and vary within the same diagnosis. Anhedonia has been suggested as such an endophenotype[5], which occurs in several psychiatric diagnoses, but where the biological basis may be common and where a specific treatment may be a feasible way forward.

Anhedonia - the inability to feel pleasure or reduced motivation to seek pleasure - is associated with affective disorders, but also with other psychiatric disorders such as addiction and schizophrenia[6]. Severe anhedonia is present in about 40% of all cases of major depression and is a risk factor for developing depression with more severe symptoms, a longer course of illness and treatment resistance, as well as relapse into depression[7,8]. Currently, there is no effective and/or specific treatment for anhedonia. The onset of anhedonia itself is thought to be due to dysfunction in the brain's reward

system[9]. An important structure in the brain's reward system is the nucleus accumbens (NAc). fMRI studies have shown that neural activity in the NAc increases when there is an expectation of a reward. In patients with symptoms of depression and anhedonia, the corresponding activity is reduced compared to healthy individuals[10]. In the NAc, there are neurons that express the dopaminergic receptor D3[11]. The D3 receptor is considered a potential target for the treatment of anhedonia, and the antidepressant effects of D3 receptor agonists have been demonstrated in animal studies[12]. Most of today's drug treatments for depression, such as monoamine oxidase (MAO) inhibitors and bupropion, affect dopaminergic neurotransmission but none acts as a specific D3 receptor agonist.

Anhedonia and similar symptoms have also been associated with low-grade, chronic systemic inflammation[13]. For example, depressed patients with increased levels of inflammatory markers in their blood have reduced connectivity ("communication") between the reward system and other parts of the brain[14]. Several studies have also shown that inflammation affects the turnover of dopamine in the brain, and patients with concomitant low-grade systemic inflammation and depression respond better to bupropion (which increases dopamine in the synapses) compared to other patients with depression without increased inflammation[15]. Thus, drugs that affect dopaminergic nerve transmission are likely to have a better treatment effect in depressed patients with signs of systemic inflammation compared to patients with depression at large.

Pramipexole is a selective D3 receptor agonist mainly used as a treatment for Parkinson's disease (PD) and Willis-Ekbom disease (Restless Legs Syndrome). Patients with PD often suffer from depression with anhedonia symptoms and it has been suggested that pramipexole has antidepressant effects in these patients by affecting dopaminergic neurotransmission[16]. The presumed antidepressant effect of pramipexole has also been investigated in several randomised trials in unipolar and bipolar depression[17-19]. The clinical effect has nevertheless been too small to justify broadly the use of pramipexole in place of conventional antidepressants, which have fewer adverse reactions. However, these studies have used relatively low doses of pramipexole (on average 0.75 mg base [equivalent to about 1 mg salt]), while PS is treated with pramipexole at between 0.26 and 3.15 mg base (3.15 mg base is equivalent to 4.5 mg salt). *The American Journal of Psychiatry* published a clinical review of patients

with refractory bipolar or unipolar depression treated with pramipexole as adjuvant therapy. Interestingly, 76% reported an improvement that persisted up to 16 months after completion of treatment with an average dose of 1.75 mg base pramipexole (equivalent to 2.5 mg salt)[20]. These results are promising because the patients in the study had a high degree of resistance to therapy (several had not responded to ECT, for example), but the study was uncontrolled and took place within "clinical routine". Thus, well-controlled and systematic studies are needed before pramipexole can be recommended as a treatment for these patients.

Depression is associated with impaired cognitive functioning, leading to difficulties in memory, attention, psychomotor speed, and executive functions (EF) (Hammar and Ardal, 2009). Cognitive impairments affect many areas of everyday life and may have important implications for diagnosis, treatment, and rehabilitation. The literature describes three profiles that may explain neurocognitive difficulties in depression (Hammar, Ronold et al., 2022). One explanation is that impaired cognitive function is related to depressive symptoms and therefore improves in parallel with treatment response (State). Another profile suggests that neurocognitive difficulties are irreversible due to the neurotoxic depressive state (Scar), while the final perspective proposes that there is a neurocognitive vulnerability that predicts depression (Trait). However, it remains unclear what the neurocognitive functioning profile looks like in treatment-resistant depression, which is characterized by high anhedonic symptom burden, and how it is affected by dopamine-regulating medication.

In this randomised and placebo-controlled trial, we want to test the antidepressant effect of adjuvant treatment with pramipexole in the higher dose range (> 1.05 mg, max 3.15 mg base) in patients with different forms of depression but where all have a symptom profile with significant anhedonia symptoms. We also perform fMRI and measure biomarkers (blood and cerebrospinal fluid) before and after treatment to detect target engagement and to identify factors that may predict a good treatment response.

9. Risk-benefit assessment

Affective disorders are associated with increased mortality in terms of increased risk of suicide, but there is also a link to increased mortality from other diseases such as cardiovascular disease[21]. The WHO has estimated that depression is the leading cause of disability in the world and that as many as one in four Swedes are at risk of suffering from depression requiring treatment at some point in their lives[22]. Given the disease's often long duration, this causes severe personal suffering and high societal costs. Affective disorders with anhedonia symptoms are associated with increased overall severity of illness, more relapses, and are also associated with increased risk of suicide[23]. There is also a high risk that anhedonia symptoms will persist after depression has otherwise improved, and there is currently no effective treatment specifically targeting anhedonia. There is therefore a great need to find a specific and effective treatment for anhedonia in depression.

Treatment with pramipexole may cause dose-dependent adverse reactions such as nausea, vomiting, orthostatic hypotension, headache or dizziness. In our pilot study, which included 12 patients with "anhedonic depression", headache, nausea and sleep disturbance were the most common adverse reactions. In elderly individuals, motor disturbances, hallucinations, delusions, confusion, mania and impaired impulse control may develop in rare but severe cases after long-term treatment with pramipexole for PS [24]. To reduce this risk in the current trial, we will screen out patients with psychotic illness, addiction and impulse control disorder, and we will follow patients over the trial period, focusing on these types of rare adverse reactions with relevant assessment instruments. We will include patients with bipolar disorder in depressive phase in our trial. A review of the literature has shown that the proportion of patients who experience a transition from depression to a manic state when treated with pramipexole is comparable to (and in some cases smaller than) those who receive placebo[25]. Unlike other dopamine receptor agonists, pramipexole can induce somnolence (especially at doses above 1.05 mg base/day according to FASS) and, in rare cases, patients have fallen asleep without warning[24]. Therefore, patients suffering from somnolence are instructed to refrain from driving and operating heavy machinery until this adverse reaction has ceased. According to FASS, pramipexole is not contraindicated in any specific disease. When treating PS , the dose should be reduced in case of concomitant renal

impairment (pramipexole is eliminated via the kidneys). Caution should also be exercised in patients with psychotic conditions and concomitant administration of anti psychotic drugs should be avoided.

Pramipexole does not have an approved indication for the treatment of anhedonia symptoms and will be administered in this trial mainly according to the Summary of Product Characteristics (SmPC) for the treatment of Parkinson's disease (maximum dose 3.15 mg base/day). This has also been done in previous clinical studies on depressed patients[17,19,26]. Some of these studies also treated with pramipexole doses above 3.15 mg[17,20]. As this is likely to increase the risk of neuropsychiatric adverse reactions[24] and is not an approved dose range in Sweden, this has not been done in the present trial. Adverse reactions are systematically monitored and recorded within the trial.

The trial is being conducted as the COVID-19 pandemic is ongoing and the risk to research subjects of participation in a drug trial during the pandemic has been addressed by following EMA guidelines (EMA/158330/2020 Ver. 1) by creating an action plan in advance in case of a new wave of COVID-19. For example, study staff will adhere to the current hygiene requirements of the clinical site where research subjects will be located.

The risk of severe adverse reactions is very low with dose ranges below 3.15 mg base/day, as confirmed by several previous clinical studies with pramipexole in depressed patients[20] as well as our own pilot data on 12 severely depressed patients. Thus, the trial's potential benefit in demonstrating an effect in refractory depression with severe anhedonia symptoms outweighs the risks for the included research subjects, since it may lead to successful treatment in these groups and increase the potential for individual-based treatment.

9.1 Experiences from our pilot study in Region Skåne (proof of concept)

In spring 2021, the pilot study PILOT-PRAXOL (EudraCT: 2019-001907-19), in which we treated 12 patients (eight women and four men) with pramipexole, was completed. This was a difficult-to-treat group which had tried a large number of antidepressants in the past without a satisfactory and sustained effect. Four patients had also previously tried ECT. Eight research subjects also underwent fMRI scanning before and after treatment. Patients were followed for a 10-week period, with doctor's visits every two weeks, where symptom assessments with MADRS, DARS-SV-MOD, SHAPS-C, AES, FSS and MAP-SR were performed. Blood samples were taken before starting treatment and after ten weeks, for analysis of inflammatory markers (hs-CRP, leukocytes, IL-6 and TNFα). The dose of pramipexole was escalated gradually. The mean dose of pramipexole among participants was 2.51 mg base/day (equivalent to 3.59 mg salt/day). Among adverse reactions that occurred, headache and nausea were the most common. All reported adverse reactions were mild to moderate in degree. Only in two cases did adverse reactions persist after study completion, with one patient experiencing persistent sleep disturbance and another experiencing persistent anxiety and elevated liver transaminases, the latter considered unlikely to be related to pramipexole treatment. Adverse reactions did not cause any study drop outs.

Treatment with pramipexole was associated with a significant improvement in both anhedonic symptoms (as measured by SHAPS and DARS-SV-MOD) and general depression symptoms (as measured by MADRS). In addition, we saw a significant decrease in CRP during the treatment period. Four patients showed an improvement in MADRS of at least 50%. In seven patients, anhedonic symptoms, as measured by DARS-SV-MOD, were reduced by at least 50%.

10. Aims and questions

The aim of the project is to administer pramipexole as an adjuvant to ongoing antidepressant treatment of patients with affective disorders with a symptom profile characterised by anhedonia. This trial may fill an important knowledge gap because i) we will include only those patients with significant anhedonia symptoms regardless of baseline diagnosis, ii) we will use anhedonia symptoms as the primary outcome measure, iii) we will use higher doses of pramipexole compared to previous studies and iv) we will perform fMRI and explore biomarkers to predict treatment response in the future.

In order to achieve a higher dosage and sufficient treatment effect compared to previously, and avoid intolerable adverse reactions, a flexible dosing schedule will be used. According to the literature, this should be an average dose of about 1.75 mg base/day but is expected to vary between individuals. In our pilot study, the mean dose of pramipexole among participants was 2.51 mg base/day (equivalent to 3.59 mg salt/day). For example, D3 receptors are known to vary with age (fewer at older ages) and thus older individuals tolerate and require higher doses to achieve treatment efficacy.

In order to get a better understanding of the biological mecanisms related to the symptom anhedonia blood and CSF(substudy) samples are taken for analysis of biomarkers related to inflammation, cellular health (incl. neurodegeneration), consentration of pramipexole, cellular stress and metabolism, growth factors, monoamine biomarkers (and receptors) and for genetic analysis relevant to these biological systems. We will also analyze biomarkers related to the blood-brain barrier and its drug transporters and the concentration of the investigational drug.

In addition, an fMRI study (MID task: reward-system test) is planned to be performed before and after treatment with pramipexole, which can be used in future follow-up studies to investigate reward-system dysfunction in anhedonia and for predictive analyses of the treatment effect of pramipexole. In order to compare fMRI examinations and biomarkers between depressed patients and healthy controls, we will also recruit up to ~~40~~ 60 healthy controls who will undergo blood sampling, lumbar puncture, fMRI and neuropsychological testing (see below) including Probabilistic Reward Task (PRT) according to the same protocol as the depressed patients. The healthy controls are recruited through advertisements

in various ways (e.g., social media, web, newspapers, radio, community, or TV). An attempt will be made to match the healthy controls with the depressed patients regarding sex, age, educational level, and physical illnesses. The healthy controls receive the same financial compensation as depressed patients for their participation in the sub-studies MRI, lumbar puncture, and neuropsychological testing, including the Probabilistic Reward Task (PRT).

Regarding neuropsychological testing and self-assessment of cognitive function, the cognitive functions of psychomotor speed and attention are expected to be related to the treatment effect on anhedonia (the State profile), with an expected improvement at testing after 9 weeks of treatment and after 6 months, compared to before treatment initiation. Executive function is expected to be Trait/Scar-related, and therefore no change in executive function is expected; instead, it is expected to be related to relapse/recurrence. No change in memory functions is expected.

10.1 Primary question and variable

Primary question: Will treatment with pramipexole at the highest possible dose (without intolerable adverse reactions, max 3.15 mg base) reduce anhedonia symptoms over a nine-week period compared to placebo treatment?

Primary variable 1: Total SHAPS-C scores at baseline, week 3, week 6 and week 9 (endpoint).

10.2 Secondary questions and variables

Secondary question 1: Will treatment with pramipexole at the highest possible dose (without intolerable adverse reactions, max 3.15 mg base) reduce depression symptoms over a nine-week period?

Secondary variable 1: HDRS6 scores (subscale of HDRS-17) at baseline, week 3, week 6 and week 9 (endpoint).

Secondary question 2: Will treatment with pramipexole at the highest possible dose (without intolerable adverse reactions, max 3.15 mg base) increase daily physical activity, reduce stress and improve sleep quality over a nine-week period?

Secondary variables 2: Number of steps/day, movement pattern distribution over the day, walking distance, time spent in light, moderate and intense physical activity, resting heart rate, blood oxygen saturation, heart rate variability *(stress scores*), sleep latency (time to fall asleep), sleep awakening (how often you wake up during the night), wakefulness (time in minutes awake during a night), time in deep sleep, sleep efficiency (time asleep vs. total time in bed). All variables are measured using activity meters which are CE-marked.

Secondary question 3: Will treatment with pramipexole at the highest possible dose (without intolerable adverse reactions, max 3.15 mg base) reduce psychological symptoms and increase quality of life?

Secondary variables 3: Total scores of DARS-SV-MOD, MADRS-S, *Insomnia Severity Scale,* AES, GAD-7 and BBQ.

Secondary question 4: Will treatment with pramipexole at the highest possible dose (without intolerable adverse reactions, max 3.15 mg base) increase the activity of the ventral striatum in the context of the MID test at fMRI?

Secondary variables 4: BOLD activity in the nucleus accumbens during MID task fMRI.

Secondary question 5: Can biomarkers in blood and CSF, ventral striatum connectivity and activity during MID task predict treatment response of pramipexole? Can genetic variants linked to dopamine and inflammatory systems be linked to improved treatment response?

Secondary variables 5: Biomarkers and genetic variants linked to inflammation, cellular health (incl. neurodegeneration), cellular stress and metabolism, growth factors and monoamine turnover.(and its receptors), the blood-brain barrier and its drug transporters, and the concentration of the investigational drug. BOLD activity in the nucleus accumbens during MID task fMRI and connectivity during diffusion tensor imaging.

Secondary question 6: Is pramipexole a safe and tolerable treatment for anhedonic depression?

Secondary variables 6: Adverse events and reactions in pramipexole and placebo groups according to the definiition section 16 below.

Secondary question 7: Is cognitive function profile realted to state (symtomload/anhedonia) or scar/trait (duration/vulnerability) after treatment with pramipexole?

Secondary varibles 7: Neuropsychological test battery consisting of WAIS-IV, RBANS, D-KEFS, CPT-3 and cognitive self-assessment PDQ-5.

Secondary question 8: Will treatment with pramipexole at the highest possible dosage (without intolerable adverse reactions, max 3,15 base) improve PRT performance over a nine week period ?

Secondary variables 8: Probabilistic Reward Task.

11. Trial design

This is a randomised, double-blind drug trial in which 80 patients are included and treated with adjuvant pramipexole or placebo for 9 weeks. The treatment trial will be supplemented by three sub-trials with fMRI examination, lumbar puncture and neuropsychological and cognitive testing in research subjects who wish to participate (not mandatory for participation in the main trial).

60 psychiatrically healthy controls will meet a research physician for diagnostic assesment and will then perform symptom assessments, lumbar puncture, fMRI, bloodsamples and neuropsychological testing with self-assessment of cognitive function including the PRT according to the same protocol as the depressed patients

12. Study population

80 research subjects with unipolar depression, bipolar disorder in depressive phase or dysthymia will be recruited to receive nine weeks of pramipexole treatment; all research subjects will have severe anhedonia as defined in Section 12.1. All research subjects are asked to participate in the sub-study (fMRI and lumbar puncture, neuropsychological testing PRT included). Only those being treated under the Health and Medical Service Act (i.e. not under compulsory care) may be included in the study.

Research subjects who meet all inclusion criteria and no exclusion criteria as described below may be included in this study.

12.1. Criteria

12.1.1. Inclusion criteria

1. Age ≥18 years ≤75 years.
2. Diagnosis of: unipolar depressive episode or bipolar disorder in depressive phase, dysthymia
3. Anhedonia symptoms: 3 or 4 points on ≥ 3 items of the Snaith-Hamilton Pleasure Scale (SHAPS-C). This has been adopted in previous studies as a definition of "clinically significant anhedonia"[27].
4. Ongoing treatment with at least one antidepressant or mood stabilizing medication ≥ 4 weeks. Has tried an antidepressant at a therapeutic dose but not achieved remission (refractory stage 1 depression)[28]).
5. The research subject has given informed consent to participate in the study.

12.1.2. Exclusion criteria

1. Pregnancy, breastfeeding or planned pregnancy (if female).
2. High suicide risk according to the overall clinical assessment of the research physician.
3. Ongoing substance abuse (within 6 months).
4. Diagnosis of current psychosis.
5. Known diagnosis of Emotionally Unstable Personality Disorder.

6. Treatment under LPT.
7. History of or strong clinical suspicion of impulse control disorder (including current binge-eating disorder) or a current ADHD diagnosis with hyperactivity.
8. Diagnosis of intellectual disability, dementia, or other circumstance that makes it difficult to understand the meaning of participating in the trial and give informed consent.
9. Diagnosis of renal failure (eGFR < 50 ml/min/1.73^{m2}) or severe cardiovascular disease (specifically symptomatic heart failure NYHA >Class II).
10. Recently started psychotherapy (within 6 weeks) or planning to start such treatment during participation in the trial.
11. Ongoing ECT, ketamine or rTMS treatment, excluding maintenance ECT, ketamine or rTMS. (Maintenance treatment is defined as the use of ECT/ketamine/rTMS for a period exceeding 3 months after a series of ECT/ketamine/rTMS treatment in order to prevent the onset of a new episode).
12. Other medical conditions, other ongoing interventions or other concomitant drug treatment (see section 14.5) which, in the opinion of the investigators, may affect the evaluability of the trial or conditions that increase trial risk. For example, Parkinson's disease, hepatic insufficiency, ongoing cancer not in remission for more than one year, obesity surgery whith known effects on the absorption of extended-release tablets in gastrointestinal system.
13. Known or suspected allergy to any active substance or excipient in the medicinal product included in the trial.
14. Participation in other treatment studies.
15. Other reason, as assessed by the investigator, that prevents the research subject's participation, such as the risk that the research subject is unable to complete the trial (non-compliance).

12.2. Criteria for withdrawal from the trial

1. Non-compliance with trial drugs and doctor's visits (see section 13.8).
2. New-onset acute suicidality.
3. In the event of unacceptable adverse reactions such as development of manic state.

4. Pregnancy.
5. The research subject may withdraw from the study at any time without consequences for his/her further treatment.
6. If research subjects are required to start a treatment mentioned in Section 14.5.

The principal investigator may discontinue a subject's participation in the study at any time, for example due to unacceptable adverse reactions, deterioration of mental health, or non-compliance with the study protocol procedures (see study compliance section 13.8).

In case of withdrawal by the research subject, a plan for discontinuation (tapering schedule) of pramipexole treatment will be established.

13. Trial implementation

Visit	Screening	Baseline(7-14 days after screening)	Between weeks 1-2, weeks 4-5, weeks 7-8, +/- 4 days	week 3, week 6, +/- 4 days	week 9 (end) +/-4 days	Follow-up by phonecall (week 13) +/- 4 days
Informed consent	X					
Medical history and diagnostic assessment	X					
Clinical assessment of co-morbidity if necessary by MINI+ (DSM Dysthymia), SUAS-S	X					
Physical examination including blood pressure, height and weight	X					
Inclusion/exclusion criteria	X					
Blood tests β-hCG (if female of childbearing age), eGFR, liver status, Hb.	X				X	
Randomisation		X				
Blood samples for biobank		X			X	
fMRI (sub-trial)		(X*)<br>‘before baseline			(X*)<br>*before baseline	
Neuropsychological testing/cognitive testing and PRT<br>(subtrial)		(X*)<br>*before baseline			(X*)<br>*before baseline	
Lumbar puncture (subtrial)		(X)			(X)	
Distribution of Medicines		X		X	(X)	
Activity measurement	X	X	X	X	X	
HDRS-6		X		X	X	
SHAPS-C	X	X		X	X	

DARS-SV-MOD, MADRS-S, Insomnia Severity Scale, AES, GAD-7 and BBQ (self-assessment)		X		X	X	
Assessment of adverse reactions			X	X	X	
U-hCG (women of childbearing age)				X (only week 3)		
Telephone contact			X			X
CGI-S				X	X	
Adverse Events			X	X	X	X

13.1. Recruitment and screening visits

We will recruit study participants in several ways: through information sent to appropriate clinics or departments where such patients are expected to be present, and by various forms of advertisement (e.g., social media, web, newspapers, radio, in the community or TV). The trial will not replace regular health care.

- Potential study participants will first be pre-screened by phone and receive study information by post or e-mail. Screening number will be assigned.
- Informed consent is obtained.
- During the screening visit, all inclusion and exclusion criteria are reviewed, including assessment with SHAPS-C[29]
- Clinical suicide-risk assessment is done based on psychiatric status and if necessary using the SUAS-S[32]. We use relevant parts of the Mini International Neuropsychiatric Interview (MINI)[33] with the addition of the DSM criteria for dysthymia as a tool in the diagnostic assessment.
- Contraindications for MRI and lumbar puncture are reviewed. If the research subject will participate in fMRI examination or lumbar puncture, information on the procedure will be provided and separate consent will be obtained.

In connection with the screening visit, the research subject will be asked about participation in a substudy involving neuropsychological testing and self-assessment of cognitive function and the test Probablistic Reward Task. After informed concent has been obtained, ,this procedure is carried out on a separate study visit.

- A clinical check of cardiac status, pulmonary status and blood pressure is performed and a note is made in the medical record of the research subject.
- The research subject's initials and birth year as well as the time of the screening visit are recorded in a screening log.

- CE-marked activity meters are distributed to start using immediately after the screening visit (baseline measurement).
- The research subject will receive a referral for testing, which should be performed at least 14days before the baseline/FMRI study (β-hCG [if female of childbearing age], eGFR, liver status and Hb). The samples are mandatory to participate in the study.
- If blood samples are normal, randomisation takes place and each research subject is identified with a unique number (enrolment log).

Before the baseline visit, the investigator has ensured that the blood test results (of hCG, eGFR, liver status and Hb) are assessed and cancels the visit if these are abnormal (referral to the appropriate health care facility for further investigation/action is then sent if necessary).Randomisation to either placebo or active treatment is performed by a staff member who is unblinded and has no contact with the patient during the trial.

One member of the study team, who does not assess patients, is unblinded during the course of the study. This staff member places packets of placebo or active treatment of different concentrations in a box designated for each research subject. From this box, investigators can then retrieve drugs for administration to research subjects during the trial without the unblinded staff member’s presence, as we have a flexible dose in the trial based on possible adverse reactions.

If a research subject does not meet the inclusion- or exclusion criteria, rescreening is allowed after at least one month.

13.2. Process for obtaining Informed Consent

After the pre-screening interview, trial information is sent home to the potential trial participant. During the screening visit, the research subject is informed orally about what trial participation means, and the research subject is given time to read the information and have any questions answered before signing the consent together with the investigator. In this context, the research subject is again informed that it is possible to discontinue the trial at any time, and that assistance with the tapering of pramipexole

will be provided if needed. The participant will be informed about the three substudies (lumbar puncture ,fMRIand neuropsychological testing including PRT) and separate inform consent forms are obtained for these voluntary sub-studies.

13.3. Before trial treatment – Baseline (7-14 days after screening)

- Prior to the baseline visit, fMRI screening is performed for the research subjects participating in the fMRI sub-trial.
- Before the research subject starts treatment with pramipexole or placebo, blood samples (and CSF if the research subject is participating in this CSF sub-trial) will be taken for storage in a biobank. In case its not possible to draw blood samples the participant is still allowed to continue the study (it doesn't include the routine samples described in 13,1).
- Study participants assessed with SHAPS-C, HDRS-6 and self-assessed with DARS-SV-MOD[34] , MADRS-S[35], AES[36], Insomnia Severity Scale[37], GAD-7[38] and BBQ[39].
- Trial drugs are dispensed by trial staff and the study participant begins treatment according to the titration schedule described in section 14.3. The dosing diary is distributed and the research subject is instructed to bring this completed diary to the next visit.
- Activity measurement results between screening and baseline are noted and activity measurement continues while treatment with pramipexole is given.

Before starting pramipexole treatment, an fMRI examination and lumbar puncture will be performed for the research subjects participating in the sub-trial(s). In the Radiology Department, the absence of contraindications for fMRI (no magnetic implants, normal renal value) is confirmed. The research subject spends approximately one hour in the scanner and receives compensation for participation after the examination (see section 21.5). Lumbar puncture is performed by delegated trial staff and reimbursement is also provided for this sub-trial (see section 13.9, 21.5).

If the research subject takes part in the substudy involving neuropsychological testing, cognitive self-assessment and Probabilistic Reward Task a separate visit will be scheduled for this purpose. The assessment consists of a test battery, including WAIS-IV, RBANS, D-KEFS, CPT-3,PDQ-5 and

Probabilistic Reward Test (PRT) which measures various cognitive functions such as intelligence quotient, psychomotor speed, attentional functions, verbal functions, visuospatial functions, immediate/delayed memory, as well as executive functions such as inhibition, verbal fluency and reward related response by *reward related response bias*. The total testing time is estimated at100–150 minutes (neuropsychological testing at 80-120 min, PRT at 20-30 min) and is conducted by delegated study personnel before treatment initiation and after nine weeks of treatment. Participants will receive compensation of 500 SEK for taking part in this substudy and up to 400 SEK in reward for PRT i.e totally the participant could receive 900 SEK in compensation for the two assessments. If desired, the research participant may receive feedback on the results of the neuropsychological assessment.

13.4. Treatment allocation

After the screening visit and randomisation, an unblinded staff member places the correct packs of either placebo or active treatment in different concentrations to be distributed during the study in a box unique to each research subject. A logbook of the packages is placed in each box. Study participants are given oral and written information on how to take the capsules.

13.5. Visits during treatment

Every third week during the study, the research subject will be called for a physical visit to discuss symptom assessment, titration plan and adverse reactions (using rating scales):

- New estimates including SHAPS and HDRS-6.
- Monitoring of adverse reactions, such as the onset of mania and impulse control disorders such as gambling addiction, is carried out using a form based on the Young Ziegler Mania Rating Scale (YMRS)[40], Problem Gambling Severity Index (PGSI)[41] and Questionnaire for Impulsive-Compulsive Disorders in Parkinson's Disease (QUIP[42]).
- Compliance is assessed as sufficient for continued participation in the study (see section 13.8).

- If intolerable adverse reactions develop, the research subject will be asked to contact the investigator and the strategy will be to return to the last tolerable dose and wait seven days before attempting a new dose increase (see section 14).
- CGI-S rating is performed by the study physician with regard to the primary diagnosis (depression,bipolar disorder or dysthymia).If the CGI-S score is 1 or 2 further dose escalation of the study drug is discontinued.
- Checking activity meters
- At the time of the visit, the research subject receives investigational medicinal products until the next scheduled visit (with a good margin) and information on how to take them. Unused medicines are collected for compliance checks. Dispensed and returned trial medicines are logged.
- Between visits, research subjects are contacted by telephone to check on their titration status and possible adverse reactions.

13.6. Final visit

After nine weeks, a final visit is made. New blood samples for the biobank are taken at the time of the visit. New fMRI examination and lumbar puncture will be performed on the research subjects participating in the sub-trial. The maximum tolerable dose of pramipexole is noted and new psychometric estimates are performed. Activity meters are read and submitted. At week 13 any adverse events are followed up by phone, and the participant is thereafter withdrawn from the study.

After completing week 9 in treatment , the study participant is given the opportunity to participate in a follow- up study (EU-nummer: 2022-502270-17-00) and continue the treatment for another 6 months, or start treatment whit pramipexol if the participant has recieved placebo and still fullfill all the inclusion criteria and none exclusion criteria. If the research subject is randomised to active treatment and want`s to taper the medication it will be done by the unblinded physician between week 9 and week 13.

13.7. Visits in the event of premature study withdrawal

If the research subject wishes to withdraw from the trial voluntarily or if it is deemed inappropriate by the investigator to continue (e.g., due to non-compliance, unacceptable adverse reactions, deterioration of mental health), the subject will be offered a new visit or telephone contact with the investigator to establish a tapering schedule. Premature withdrawal is recorded and significant and/or serious incidents are followed up by the investigator after four weeks until the investigator considers it closed, or after two attempts to contact without success.

If a patient unexpectedly and suddenly becomes unreachable, informed consent allows research personnel to contact relatives (if their information is available in the medical records) to ensure that no accident has occurred or any other event that might have led to the patient abruptly and undesirably discontinuing their treatment, risking withdrawal symptoms.

13.8. Trial Compliance

The investigator may choose to terminate a research participant's involvement in the study due to inadequate compliance, such as the participant not attending physical visits or taking study product for too few days or at an incorrect dosage. This is checked by keeping a dosage diary at the doctor's visit. Investigators may also terminate the subject's participation if the intake of pramipexole has exceeded the planned amount.

13.9. Biological sampling procedures

13.9.1. Handling, storage and destruction of biological samples

Blood and CSF samples are collected, aliquoted, frozen at -80 degrees Celsius and stored in biobank. Examples of subsequent analyses are markers of inflammation, cellular health (including neurodegeneration), concentration of pramipexole, cellular stress and metabolism, growth factors and monoamine metabolism (and receptors), as well as various analyses of genetic variants relevant to these biological systems.. We will also analyse biomarkers related to the blood-brain barrier and its drug transporters, as well as the concentration of the investigational drug.

Lumbar puncture are performed at baseline and at the final visit for the research subjects included in the CSF sub-trial. Lumbar puncture is performed in a sitting position and the samples are aliquoted, frozen at -80 degrees Celsius and stored in a biobank.

13.9.2. Total blood volume and volume of spinal fluid per research subject

At baseline and final visit, blood samples with a total blood volume of 36 ml (12 ml whole blood, 12 ml plasma, 12 ml serum) are provided. Thus, a total of 72 ml is provided during the trial.

The research subjects undergoing lumbar puncture will have 12 ml of CSF taken at a time. Thus, a total of 24 ml is provided during the trial.

13.9.3. Biobank

All samples taken in this trial are registered in a biobank at Region Skåne Biobank and are handled according to current biobank legislation and regulationsSamples are coded/pseudonymised to protect the identity of the research subject. All samples and the identification list/code list are stored securely and separately to prevent access by unauthorised persons. The samples will be stored maximum for 15 years.

14. Trial drugs

14.1. Investigational medicinal products

In the trial, the investigational medicinal product contains a placebo or the active substance pramipexole[1]. In Sweden, this drug was previously marketed under the brand-name Sifrol™ but is now available in several generic versions. The dosage of pramipexole can be given either as a base or as a salt. In the FASS , the dosage as a base is primarily indicated and the corresponding concentration in salt is given in brackets. In this protocol, dosage is given in base form unless otherwise stated.

Pramipexole is approved in the EU and is indicated for the symptomatic treatment of idiopathic Parkinson's disease, alone (without levodopa) or in combination with levodopa, in the maximum dose of 3.15 mg base. Pramipexole is also indicated as symptomatic treatment of moderate to severe idiopathic restless legs syndrome in doses up to 0.52 mg base. Both apply to adults and not to children. However, pramipexole has no approved indication for the treatment of affective disorders. In this study, the research subjects will reach at most the maximum dose of 3.15 mg base/day, which is already approved for treatment in Parkinson's disease.

Placebo manufacturing is done with GMP contract manufacturing to replicate the strengths 0.52, 1.05 and 2.1 mg base pramipexole extended-release tablets from STADA Nordic. The placebo tablets do not contain any active substance.

Common Side-Effects according to FASS:

Very common (≥ 1/10): Somnolence, Dizziness, Dyskinesia and Nausea. **Common (≥ 1/100, < 1/10):** Insomnia, Hallucinations, Abnormal dreams, Confusion, Behavioural symptoms of impaired impulse control and compulsive behaviour, Headache, Visual impairment

1

including diplopia, blurred vision and reduced visual acuity, Hypotension, Constipation, Vomiting, Fatigue, Peripheral oedema, Weight loss including decreased appetite.

14.2. Handling and labelling of Investigational Medicinal Products

Purchasing of active tablets from (STADA Nordic), labelling and repackaging of-active tablets in bottles with seven tablets per bottle is handled by another contract manufacturing company. The same contract manufacturing company is manufacturing and packaging the placebo tablets. The drug is then distributed by the investigators during the visits and taken from a box unique to each research subject where a staff member delegated to the study has placed the correct packs depending on the randomisation outcome.

14.2.1. Labelling of Investigational Medicinal Products

Labelling and distribution of the investigational medicinal product is carried out according to EU guidelines and GMP of the companies overseeing management and labelling (see section 14.2).

14.2.2. Traceability of Investigational Medicinal Products

Traceabilitiy is documented in a medication-log i.e which number each bottle has and which subject the bottles are distributed to. Unblinded staff are aware of which treatment each subject has got and handle the traceability documentation of which bottle contains placebo or pramipexole. Investigators and research subjects are responsible for returning unused tablets to the pharmacy for destruction.

14.2.3. Storage of Investigational Medicinal Products

Storage is in locked cabinets, room temperature 15-30 degrees Celsius, temperature logging carried out), protected from direct sunlight.

14.3. Dosage and Drug Administration

The trial is based on the hypothesis that it is necessary to reach as high a daily dose as possible of pramipexole to obtain the expected effects (reduced anhedonia) reported in other studies[17,20]. Thus, in case of intolerable adverse reactions, the dosage will be lowered and a new attempt to increase the dosage will be made at a later date (adverse reactions such as nausea, headache, etc. usually disappear

after one week). The trial uses extended-release tablets taken once per day. In a Phase I trial, the lowest and highest plasma concentration and exposure were equivalent for the same daily dose of pramipexole extended-release tablets administered once daily and pramipexole tablets administered three times daily. Once-daily administration of pramipexole extended-release tablets causes fewer fluctuations in plasma concentration of pramipexole over 24 hours compared to thrice-daily administration of immediate-release pramipexole tablets.

During the baseline visit, packs containing one week's treatment are distributed. Up-titration is made weekly according to the following schedule based on FASS (alternative schedules available for those research subjects who do not reach the maximum dose of 3.15 mg base).

	**Treatment**	**Placebo**		**Pramipexole**	
**Week**	**Step**	**Capsule 1**	**Capsule 2**	**Capsule 1**	**Capsule 2**
**1**	1 (0.26 mg base/day)	Placebo	None	0.26 mg	None
**2**	2 (0.52 mg base/day)	Placebo	None	0.52 mg	None
**3**	3 (1.05 mg base/day)	Placebo	None	1.05 mg	None
**4**	4 (1.67 mg base/day)	Placebo	Placebo	1.05 mg	0.52 mg
**5**	5 (2.1 mg base/day)	Placebo	None	2.1 mg	None
**6**	6 (2.62 mg base/day)	Placebo	Placebo	2.1 mg	0.52 mg
**7**	7 (3.15 mg base/day)	Placebo	Placebo	2.1 mg	1.05 mg
**8**	7 (3.15 mg base/day)	Placebo	Placebo	2.1 mg	1.05 mg
**9**	7 (3.15 mg base/day)	Placebo	Placebo	2.1 mg	1.05 mg

The tablets are usually taken at night if the research subject does not have sleep problems. If intolerable adverse reactions occur with dose escalation, the subject is advised to contact the investigator and return to the treatment step that did not cause adverse reactions. After that, subjects wait another seven days before trying to go up a step again. This is done on a maximum of two occasions before giving up trying to reach the higher dose (treatment continues at the lower dose if possible). The total treatment time is nine weeks before tapering. If the research subject is considered to be in remission (

CGI-S 1or2) in an overall clinical assessment, further dose escalation is stopped. Compliance is monitored by asking the research subject to complete and bring a dosing diary to the evaluation visits every third week. At the conclusion of the study, investigators are responsible for the return and destruction of leftover tablets except for those needed for tapering.

Pramipexole treatment must not be terminated abruptly because of the risk of *dopamine agonist-withdrawal syndrome* (DAWS) with symptoms such as depression, anhedonia, apathy, anxiety, sleep disturbances, etc. The study participant has the right to withdraw from the study at any time without giving a reason to the research physician. However, research physicians have a responsibility to try to check with the research subject regarding the possibility of safely tapering. According to FASS, tapering is recommended at one dose-step/day. In the trial, we assume a dose-step reduction every other day, but this can be individualised based on any symptoms during tapering.

14.4. Non-trial medicines

The research subject must be on a stable dose of at least one antidepressant or mood stabilising drug for at least four weeks prior to the start of treatment (no ongoing dose changes)

In order to ensure that any clinical effect is dependent on pramipexole, it is recommended that study participants do not make any dose adjustments to their ongoing antidepressant or mood stabilising medication during the course of the trial. If the investigator, the PAL and the patient determine that the patient’s clinical condition nevertheless requires an adjustment of current medication, this shall be done. The study participant may still continue pramipexole as planned, the change is noted in the patient's study log and this is taken into account in the data analysis.

14.5. Concomitant medication

If possible, concomitant medication should not change during trial participation. In the event of worsened condition, this may be done without leaving the trial. Other medicines prescribed to the research subjects will be managed as usual by the respective clinics. All concomitant medications are

allowed while the research subjects are included in the study, except for those medications that fall under exclusion criterion #12, that is:

i) Ongoing treatment with drugs that affect plasma levels of pramipexole. E.g., cimetidine, amantadine, mexiletine, zidovudine, cisplatin, quinine and procainamide.

ii) Ongoing treatment with medicinal products having a similar or antagonistic mechanism of action to pramipexole. E.g., MAO inhibitors, levodopa, dopamine agonists, metoclopramide (Primperan), also applies to central nervous system stimulants such as methylphenidate and amphetamines (not caffeine). Does not apply to bupropion.

iii) Ongoing treatment with neuroleptic drugs. E.g., olanzapine, haloperidol, risperidone, clozapine, aripiprazole, quetiapine (Seroquel). Low-dose (=<150 mg/day) quetiapine is permitted as it has very low binding to D2 receptors at such doses[43].

After washout period of > 14 days of all drugs, patient can be included in the study.

14.6. Randomisation

The randomisation process is done by a randomisation list developed by statistician at Clinical Studies Sweden, Forum South. The blinded personel in the study has no knowledge of the outcome of the randomization.

Research subjects are included/randomised consecutively as they are found suitable for inclusion in the trial. If a research subject withdraws from the trial, the research subject code will not be reused, and the research subject will not be allowed to be included in the trial again.

14.7 Blinding

All investigators who meet study participants are blinded to the study. There will also be unblinded staff in the trial to carry out randomisation and arrange drug distribution.

14.8. *Code breaking*

In the box with study medication that are distributed to each subject (section 13,4) there will be an code envelope (for code-breaking in emergency or if the electronic system fails) by the unblinded staff who has access to the outcome of randomisation and the medication-log with the information which treatment (placebo or pramipexol) the subject recieved. Code breaking normally taks place when the subject meets the unblinded physician who decides the need of taper och continue the treatment after endning the study (see section 13.6)

15. Methods for collecting study variables

Study variables are collected using clinical assessment, assessment questionnaires, activity meters, blood/cerebrospinal fluid sample analysis and fMRI examination.

15.1. Effect variables

Outcome measures related to symptom severity are assessed using rating scales (SHAPS,HDRS6,DARS-SV-MOD, MADRAS-S, *Insomnia Severity Scale, AES,GAD-7 and BBQ)* at screening and baseline visits and every third week for 9 weeks of investigator visits. For some research participants, data collection also takes place 6 months after the end of the study participation.

Biobank samples are collected at baseline and at the final visit. Other secondary variables are collected by activity monitors, during the fMRI examination, lumbar puncture, neuropsychological testing and Probabilistic Reward Task. Activity meters are distributed at the screening visit and returned at the final visit. During the physical visits, the use of the meter is checked, i.e., that it is functional and the battery is charged, the data of activity is downloaded. Research subjects should wear the meter throughout the trial (including during sleep) except when it must be removed for hygienic reasons.

15.2. Safety and tolerability variables

15.2.1. Laboratory variables

Routine laboratory tests (blood tests) consist of eGFR, liver status (ASAT, ALAT, GT, ALP), Hb value and β-hCG and are done between the screening and baseline visits as well as at the final visit. Abnormal/unexpected measurements according to Labmedicin Skåne (Region Skåne) and clinical significant values according to the study physician that occurs after the screening, are recorded in the medical record with comment on how these may be followed -up. In the event of abnormal and clinically significant values observed during the study i.e identified at the final visit but not present on baseline, are recorded and additionally reported as adverse eventsin the trial (see section 16.5 below). In case of mild to moderat abnormal test results , new tests can be taken at the follow-up visit (week 3)

15.2.2. Vital signs

Cardiac and pulmonary status and blood pressure monitoring are performed according to the clinic's standard procedure at screening visits. Abnormal/unexpected vital signs at screening (not previously known cardiac murmur. ystolic blodpressure > 150 mmHg or < 100 mmHg is recorded and reported as incidents in the study together with a plan for possible follow up.

15.2.3. Tolerability variables

Adverse reactions to pramipexole treatment are collected during visits with investigators (every two weeks). In the event that intolerable adverse reactions develop (subjective assessment by participants) between two visits, the subject is advised to contact the investigator and the strategy will be to return to the last tolerable dose and wait 7 days before attempting a new increase (see section 14).

16. Safety monitoring and reporting

16.1. Definition of an adverse event

Term	Definition
Adverse Event/Incident (AE)	An adverse medical event or worsening of an existing condition in a research subject who has received a medicinal product and which is not necessarily causally related to the treatment given. Examples of incidents: symptoms nausea, somnolence, development of hypertension, development of mania.
Side effect/Adverse drug reaction (ADR)	An adverse medical event that is judged by a medically competent person to have a reasonable probability of being causally related to the trial drug at any dose.
Serious Incident /<br><br>Serious Adverse Event (SAE)	An adverse medical event that meets one or more of the following criteria:<br>1. Death.<br>2. Life-threatening condition. The term "life-threatening" refers to an event where the participant was at risk of death at the time of the event. It does not refer to an event that hypothetically would have caused death if it was more serious.<br>3. Requires hospitalisation or extends a hospital stay already started.<br>4. Causes a permanent or significant disability or impairment.<br>5. Constitutes a congenital malformation or congenital defect.<br><br>Other medically significant event for the research subject that requires intervention to prevent one of the above criteria.
Side Effect/	An adverse medical event that is both serious and judged by a medically competent person to have a reasonable probability of being causally related to the study drug.

Serious Adverse Reaction (SAR)	
SUSAR<br>Suspected Unexpected Serious Adverse Reaction (SUSAR)	A suspected serious adverse reaction that is unexpected, i.e., the nature and severity of which is not consistent with known data on the medicinal product as provided in the reference safety information (Investigator's Brochure [IB] or Summary of Product Characteristics).

16.2. Reference safety information

The summary of product characteristics (SmPC) for the pramipexole extended-release tablet (STADA Nordic) is used for the assessment of the relationship between incidents and the medicinal product and the assessment of a suspected unexpected serious adverse reaction (SUSAR).

16.3. Assessment of incident severity

The investigator is responsible for assessing the severity (serious or non-serious). If the incident is deemed serious, it should be reported as a serious adverse event (SAE) by the investigator to the sponsor. See also 16.7.2 Serious incident reporting (SAE). In the log, the severity of the incident is assessed as:

Mild: short-term/transient symptoms that do not affect the research subject's daily activities.

Moderate: noticeable/perceptible symptoms that partially affect the research subject's daily activities.

Severe: symptoms that significantly affect the research subject's daily activities.

16.4. Assessment of incidentcausality

To determine whether an incident is side effect, it should be considered whether there is a reasonable possibility to establish a causal relationship between the incident and the investigational medicinal product based on the analysis of the available evidence.

All incidents can be categorized, e.g. as probably related, possibly related, unlikely related, or not related according to the definition below:

Probably related:

A clinical event, including abnormal laboratory findings, that occurs within a reasonable time after administration of the intervention/investigational medicinal product. It is unlikely that the event can be attributed to the underlying disease or other medications, but it is most likely caused by the investigational medicinal product, and the onset is reasonable in relation to the use of the investigational medicinal product.

Possibly related:

A clinical event, including abnormal laboratory findings, that occurs within a reasonable time after administration of the intervention/investigational medicinal product. The event may be explained by the investigational medicinal product, and the onset is reasonable in relation to the use of the investigational medicinal product, but there is not enough information to establish the connection. The event may also be explained by the underlying disease or other medications.

Unlikely related:

A clinical event, including abnormal laboratory findings, that is unlikely to be related to the intervention/investigational medicinal product and can reasonably be explained by other medications or the underlying disease.

Not related:

A clinical event, including abnormal laboratory findings, that is not reasonable in relation to the use of the intervention/investigational medicinal product.

Adverse events (AEs) suspected to be associated with the investigational medicinal product will be followed up until the subject has recovered or is adequately cared for and well on the way to recovery (see also section 16.5, Follow-up of incidents (AE/SAE)).

If the reporting investigator does not provide information on causality, the sponsor shall consult with the reporting investigator and encourage them to express a position on this matter. The causality assessment provided by the investigator must be considered by the sponsor. If the sponsor does not agree with the investigator's causality assessment, both the investigator's and the sponsor's opinions shall be included in the report.

16.5 Incident registration

At each trial visit, incidents (AEs) are recorded, starting from the initiation of treatment with the investigational medicinal product and continuing up to 4 weeks after the subject has completed treatment with the investigational medicinal product. All AEs that occur during the trial and are observed by the investigator/study nurse or reported by the subject will be recorded in the CRF, regardless of whether or not they are related to the investigational medicinal product. Assessment of causality, intensity, and whether the AE is considered an SAE will be performed by the investigator directly in the CRF/on a trial-specific worksheet. As a minimum, the following must be recorded for each AE/SAE:a description of the event (diagnosis/symptom if no diagnosis is available), start and stop dates, causality, intensity, whether the AE is considered an SAE, actions taken, outcome.

Incidents are followed from the start of treatment until four weeks after the end of the visit. In such cases, there is no tapering, but incidents are nevertheless followed up four weeks after the final visit by telephone contact until the investigator considers it completed or after two attempts at contact without result.

All serious incidents will be monitored until they have been resolved or are no longer necessary to be monitored at the discretion of a medically licensed investigator. This may also apply if the incident needs to be followed up after completion of the study if it is not referred to another appropriate party.

16.6. Pregnancy

Pregnant women will not be included in the trial. Women of childbearing age must have a negative serum β-hCG pregnancy test to rule out pregnancy before starting treatment. Blood samples are taken between the screening and baseline visits and at the final visit. Pregnancy tests are given to female research subjects of childbearing age (hCG in urine) and should be taken three weeks after starting treatment. Women who become pregnant during the trial will be withdrawn but will be followed up to report any drug-induced infant injuries/malformations. The effect of pramipexole on the unborn child is unknown and treatment should be discontinued as early as possible.

16.7. Incident reporting

16.7.1. Reporting of adverse events (AEs) and adverse reactions (ARs)

All adverse events and reactions are recorded by the trial site in the CRF during the course of the trial. The principal investigator reports collected adverse events and reactions at the end of the trial with the final report to the public authorities (see below). Common and known adverse reactions such as nausea, headache and sleep disturbances are excluded from this requirement.

16.7.2. Reporting of serious incidents and serious adverse reactions (SAEs and SARs)

All serious incidents should be reported immediately to the sponsor and reported in the CRF within 24 hours of the time the investigator becomes aware they have occurred. Reporting of serious incidents and/or serious adverse reactions to the relevant public authorities is done annually through the annual safety reporting process (see below)

16.7.3. Reporting of SUSARs - suspected unexpected serious adverse reactions

The principal sponsor is responsible for reporting SUSAR. A suspected serious unexpected adverse reaction (SUSAR) to the study drug which is fatal or life-threatening is reported to the LV and EPM within 7 days of the sponsor becoming aware of the event. A report supplement is sent to the public authorities within 8 days.

A study drug SUSAR that is not fatal or life-threatening is reported to the LV and EPM within 15 days of the sponsor becoming aware of the event. A report supplement will be sent to the public authorities as soon as possible.

16.8. Annual safety report

The annual safety report is compiled by the principal investigator and sent to the LV and, upon request, to the EPM. The report summarises any serious adverse events that have occurred, as well as a summary assessment of the safety of the research subjects still enrolled in the trial, and whether the trial's benefit-risk assessment has changed since approval.

16.9. Procedure in case of overdose or other emergency

In the event of an overdose or other emergency, investigators will take safety measures as necessary to protect research subjects from immediate danger. Examples of such measures include temporarily suspending the clinical drug trial or introducing additional monitoring measures or helping the research subject to get to an emergency department (somatic or psychiatric). The Swedish Medical Products Agency and the Swedish Ethical Review Authority are informed of the urgent safety measures taken.

17. Statistical considerations

17.1. Dimensional calculation and analysis population (analyspopulation)

For the primary research question (change in SHAPS-C), a linear mixed model with repeated measures (weeks 3, 6, and 9) will be used, where the primary research question will be addressed through the overall comparison between the randomization groups. Any group differences at baseline will be adjusted for by including the baseline values as a covariate in the model. The estimated effect size of pramipexole treatment is estimated to be 0.27 as this has been reported in other studies of antidepressants[44]. This corresponds to a MADRS score difference of about 4 p which is considered a clear clinical improvement[45] and is equivalent to => 4 p on the SHAPS-C. Estimation of the correlation coefficient between repeated measures of MADRS scores is based on material from our previous pilot study ($r = 0.5$). For a power calculation with strength 80% and $\alpha = .05$, it is estimated that 74 research subjects are needed. In the initial power calculation the drop-out rate was estimated to be less than 5% (based on the pilot-study) whereby we had to include- 80 research subjects in the study. After planned interim-analysis (see section 17.3) made by external statisticians, we will need to include 80 research subjects who completes the study. The primary research question and all other secondary questions related to effect will be analysed on intention -to -treat population, that is all patients who have been randomized.

To ensure that we are able to recruit at least 80 research participants who complete the study we wlll need to randomize up to 90 participants, assuming that some will drop out during the course of the study.

Safety-population will be defined as all patients who has received medication.

17.2. Statistical analysis of primary, secondary and exploratory variables

To answer the primary research question, we use a a linear mixed model with repeated measures with severity of anhedonia symptoms (SHAPS-C total score) as the dependent variable, and time (in weeks) and treatment (pramipexole or placebo) as the independent variable. SHAPS-C total score at baseline will be used as a covariate in the model. In case of loss of measurements at different times,

these will be compensated by last observation carried forward (LOCF). Normal distribution is expected based on previous studies. Descriptive analysis performed of response (reduction 50% a MADRS-S score), remission (MADRS-S ≤ 10) of depression symptoms (exploratory variables) and number of drop outs. Descriptive analysis, mean and range, is done on the dosage of pramipexole at the final visit. Of the other secondary outcome measures, the same statistical method is used as above, i.e. a linear mixed model with repeated measures. Paired sample T-test (or appropriate non-parametric method) is used for analysis of difference of inflammatory markers and other biological measures between *baseline* and *endpoint.*-A linear mixed model with repeated measures with time x group interaction effects will be used to examine whether change in biomarkers differs between those responding to treatment and those not responding to treatment.

17.3. Interim analysis

When approximately half of the participants have completed the trial, an interim analysis of the SD on the primary outcome measure (total SHAPS-C score) and calculation of the number of drop outs is performed. A new power calculation is made based on the new SD in order to have the possibility to adjust the number of research subjects (with a new application for modification to the Swedish Medical Products Agency and the Swedish Ethical Review Authority) if necessary.

18 Data management

18.1. Data flow and processing

Data are collected by investigators in the eCRF. Data from the CRF will then be transferred to software for spreadsheet and simple data management tasks.

18.2. CRF (case report form)

The data collected in the trial will be recorded in an electronic Case Record Form (CRF) specifically designed for recording data for the current trial.

18.3. Source data documents

1. Patient record**:** Entered into a computerized medical record according to clinical procedures and clinical trial requirements. Minimum level of information to be recorded:

   i) Name of the trial

   ii) Statement that all inclusion criteria and none of the exclusion criteria have been met

   iii) Date when the patient signed informed consent

   iv) Date when the patient withdraws from/discontinues the study

   v) Details of diagnosis

   vi) Details of intake of investigational medicinal product

   vii) Details of complications

2. Consent form

3. Patient identification list**:** Included patients are assigned research numbers in the order of their inclusion. The names and personal identity numbers of the patients are recorded on the list in connection with the respective research number.

4. Case Report Form and rating scales

5. Database of data collected from activity meters.

18.4. Storage and archiving of study data and source documents / Access to data

The investigator must maintain source documents for each research subject in the trial. A document regarding what has been classified as source data in the trial should be available in the investigator site file (ISF). The investigator must ensure that all source documents are available for monitoring and other quality control purposes.

Source data are defined before the start of the trial. CRFs can be defined as source data in cases where data are not recorded elsewhere, e.g., inclusion and exclusion criteria. In these cases, data recorded directly in the CRF data are transmitted/entered according to section 18.1 above. All information is encoded so that no personal identification numbers, names, initials, addresses, etc., are included in the file. During data collection, each study participant will be assigned a specific number and a code list will be drawn up. This is kept in a safe place and is saved for the purpose of checking the quality of the trial. The code list and source data are kept locked. Monitors from Clinical Studies Sweden - Forum South will also have access to the source data after signing a confidentiality agreement.

The results of cognitive testing included Probabilistic Reward Task(PRT)are stored in paper form encoded with research number. A separate code list connecting reseach number with social security number is kept locked, The computer that will be used in connection with the PRT test will be password-protected and stored in a locked location so that only authorized personnel in the research group will have access to the research participants data. Data collected from the PRT will be regularly transferred from the test computer to an encrypted hard drive that is stored in a fireproof archive cabinet.

19. Quality Control and Monitoring

Work manuals are available on the performance of the physical visits and telephone contacts as well as the handling of CSF and blood samples. The investigators performing the SHAPS-C and HDRS6 assessments will co-score subjects prior to trial commencement and as needed to check for reliability. As change in total score is the main outcome measure, we will aim for that the same investigators will meet with the same research subject during participation if possible.

To ensure that the trial is conducted according to the protocol and that data are collected, documented and reported in accordance with ICH-GCP and applicable ethical and regulatory requirements, the trial will be monitored by an independent monitor before the trial starts, during the trial and after the trial is completed. Monitoring is carried out according to the trial's monitoring plan and aims to ensure the rights, safety and well-being of the research subjects as well as that CRF data are complete, accurate and consistent with the source data.

Patients are informed via the patient information leaflet that monitoring is carried out as well as that the monitor and any supervisory authority have access to medical records. A confidentiality agreement must be drawn up between the head of operations, who is formally responsible for patients' records, and the monitor. Monitoring of the trial is done by Clinical Studies Sweden - Forum South according to the GCP principles.

The principal investigator should ensure that the monitor has access to the CRF, patient records and original laboratory data, etc., to ensure source data relevant to the trial without compromising patient confidentiality. The principal investigator is also aware that inspections by public authorities may be carried out. Deviations from protocol are documented in a log.(protocol deviation).

20. Storage and archiving

The Principal Investigator has a trial file and the sponsor a sponsor file with relevant content according to ICH GCP Chapter 8 Essential Documents. The Principal Investigator will keep the trial data, the research subject identification list, original research subject information and the consent obtained for the trial inaccessible to unauthorised persons, but such that the research subjects in the trial can be identified by those responsible for the trial.

A complete trial file as well as source documents will be archived for at least 25 years after the trial report is written and submitted to LV. Source data in patient record systems are stored and archived according to Region Skåne's regulations.

The Archives Act (1990:782) and the Guidance (2011:19) to the Medical Products Agency's regulations on clinical trials of medicinal products in humans apply to the archiving of research material. The regulations and general advice of the National Archives of Sweden on the deletion of documents in national authorities' research activities, RAFS 1999:1, and the Region's instructions will be followed.

21. Ethical and regulatory concerns

The trial is conducted in accordance with the current version of the Helsinki Declaration, ICH-GCP-E6 rev2 and applicable international and national regulatory frameworks. The trial can begin when written approval from EPM, permission from LV and other approvals required by the current regulations are available. The Principal Investigator is responsible for ensuring that the content of the individual documents submitted to the LV in an application is identical to the content of the corresponding documents in the application for authorisation at the EPM. Approval of notification of personal data processing; approval from KVB for withdrawal from the medical record for research; registration in DSF for personal data processing; and approval of notification to Bild och funktion Region Skåne must be obtained.

21.1. Major changes to the trial

If major changes to the trial implementation or protocol (including research subject information) are made after approval, an amendment addendum must be written and sent by the Principal Investigator to the EPM and/or LV for approval before this change may be implemented (unless performed to prevent a safety risk). The respective public authorities' instructions regarding the changes to be applied for and approved by them will be followed. Major changes may include, e.g., a change in inclusion/exclusion criteria, a change in the investigational medicinal product or a change in the main investigator (the person responsible for the applications).

21.2. Serious breaches

Serious breaches in trial protocol, GCP or other regulations which significantly and directly affect, or would most likely affect, the research subjects in Sweden or the scientific value of the trial must be reported immediately within 7 days to the LV. It is the responsibility of the Principal Investigator to assess the consequences of any breaches that occur, and thus also to decide whether the LV should be informed.

Minor breaches that do not affect the integrity or safety of the research subjects, or significantly affect the scientific value of the trial, are documented with the Principal Investigator.

2.3. Reporting the end of the trial

Within 90 days of the trial's completion (see end of trial definition below), the LV and EPM will be notified of the end of the trial. Sponsor may terminate a trial at any time with cause. This may be when the benefits of the trial no longer outweigh its risks. In such cases, the LV and EPM must be informed in writing within 7 days.

21.4. Informing research subjects and obtaining consent

All patients are carefully informed about trial design and how the trial will be conducted and evaluated. The investigator is responsible for ensuring that each research subject has understood the explanation of the purpose and the design of the trial, and has received written and verbal information about its expected benefits and risks.

Research subjects have the opportunity to choose between the trial treatment and therapy provided according to clinical practice. They are given the opportunity to ask questions about the trial before consent is given. It is stated that the participation of the research subject is voluntary and that participation may be terminated at any time without impairment of medical care. The signature of the research subjects grants consent to participate in the trial and grants the monitor and pharmaceutical authorities (including foreign authorities) access to the trial source data under confidentiality.

The investigator (doctor) who informed the patient will certify with his signature that the patient has received (and understood) the information and has given his consent to the trial. A copy of the patient information as well as the signed and informed concent is provided to the research subject.The research subject's signed and dated informed consent must be obtained before any trial-specific activity is carried out. Each research subject participating in the trial will be identified with a trial-specific ID on a subject identification list.

21.5 Compensation for sub-trial participation

The research subjects who participate in the sub-study (fMRI study) receive compensation of SEK 750 for participation plus a maximum of SEK 500 (typically approx. SEK 250) as "rewards" in the MID

task. SEK 750 is an incentive to participate in the fMRI study and is common compensation in fMRI studies[46]. The level is balanced to be sufficient as an incentive to participate in the fMRI trial but not as an incentive to participate in the main trial as a whole. SEK 0 to 500 is an accepted compensation in the form of "rewards" in the MID task itself and is a necessity for performance as the task examines the activation of the reward system when monetary benefits are received (see appendix 27.1).

Research subjects who also undergo lumbar puncture will be reimbursed SEK 1,250 per test session

Research subjects undergoing in neuropsychological testing and cognitive self-assessments will be reimbursed SEK 500 after the two sessions.

21.6 Dataprotection

The collection of research data will be carried out in accordance with the General Data Protection Regulation (GDPR). In the information provided to research participants, they will receive full details on how the collection, use, and publication of their trial data will be handled. The content of the informed consent form complies with relevant privacy and data protection legislation. The information provided to research participants, as well as the informed consent form, explains how the trial data will be stored in order to maintain confidentiality in accordance with national data legislation All data processed by the sponsor will be pseudonymized and identified with a subject-specific ID.

The informed consent form will also explain that, for data verification purposes, representatives appointed by the sponsor, as well as the relevant authority, may request access to parts of hospital or trial records that are relevant to the trial, including the research subject’s medical history.

22. Timetable and definition of end of the trial

The trial is expected to start on 1 October 2022 and to be completed by 1 December 2026. The end of the trial occurs when the last research subject (at least 80 patients) has completed the last study visit and the follow-up telephone contact has been made.

23. Insurance

Study participants are covered by patient insurance (Swedish Patient Injury Act) when the study is conducted within the framework of the County Council's health care. Research subjects are insured by Swedish Pharmaceutical Insurance (LFF) of LFF Service AB.

24. Funding

The study has received funding from the Swedish Research Council (grant number 2020-01428), Swedish governmental funding of clinical research (ALF) (Award/Grant number is not applicable), grants from the province of Scania (Award/Grant number is not applicable), the Crafoord Foundation (grant number 20220522), the Brain Foundation (FO2022-0050 & FO2025-0058-HK-168), Ellen and Henrik Sjöbring Foundation (Award/Grant number is not applicable), Söderström – Königska Foundation (grant number SLS-969692), Region Kronoberg (Award/Grant number is not applicable), Bror Gadelius Foundation (Award/Grant number is not applicable), the Olle Engkvist Foundation (grant number 214-0363), John Hains Foundation, and the Sten K. Johnson Foundation.

25. Registration, reporting and publication

25.1. Registration

The trial protocol is published in the public register EU Clinical Trial Register (EU CTR), via EudraCT application to LV before inclusion of the first research subject. We have also registered the study in clinical trials.gov. We plan to publish the trial protocol in a peer-reviewed journal.

25.2. Reporting and publication

In accordance with the Helsinki Declaration, trial results will be made publicly available as soon as possible via publication and/or public database after the end of the study, and no later than one year after trial end, regardless of whether the results are positive, negative or neutral.

25.3. Results, preliminary and final, may be presented at national and international meetings.

The trial is planned to lead to scientific article(s) intended for an international peer-reviewed journal. Publications will also constitute the final report to LV and EPM. Authorship is determined in accordance with the guidelines of the International Committee for Medical Journal Editors (ICMJE). All authors must contribute to, review, and accept the final publication.

26. Referenslista

1. Penn, E. & Tracy, D. K. The drugs don’t work? antidepressants and the current and future pharmacological management of depression. Therapeutic advances in psychopharmacology 2, 179–88 (2012).

2. Nierenberg, A. A. Residual Symptoms in Depression. The Journal of Clinical Psychiatry 76, e1480–e1480 (2015).

3. Howland, R. H. Sequenced Treatment Alternatives to Relieve Depression (STAR*D). Part 2: Study outcomes. Journal of psychosocial nursing and mental health services 46, 21–4 (2008).

4. Hyman, S. E. The diagnosis of mental disorders: the problem of reification. Annual review of clinical psychology 6, 155–79 (2010).

5. Pizzagalli, D. A. Depression, Stress, and Anhedonia: Toward a Synthesis and Integrated Model. Annual Review of Clinical Psychology 10, 393–423 (2014).

6. Gorwood, P. Neurobiological mechanisms of anhedonia. Dialogues in clinical neuroscience 10, 291–9 (2008).

7. Pelizza, L. & Ferrari, A. Anhedonia in schizophrenia and major depression: state or trait? Annals of general psychiatry 8, 22 (2009).

8. Uher, R. et al. Depression symptom dimensions as predictors of antidepressant treatment outcome: replicable evidence for interest-activity symptoms. Psychological medicine 42, 967–80 (2012).

9. Treadway, M. T. & Zald, D. H. Reconsidering anhedonia in depression: lessons from translational neuroscience. Neuroscience and biobehavioral reviews 35, 537–55 (2011).

10. Satterthwaite, T. D. et al. Common and Dissociable Dysfunction of the Reward System in Bipolar and Unipolar Depression. Neuropsychopharmacology : official publication of the American College of Neuropsychopharmacology 40, 2258–68 (2015).

11. Hall, H. et al. Autoradiographic localisation of D3-dopamine receptors in the human brain using the selective D3-dopamine receptor agonist (+)-[3H]PD 128907. Psychopharmacology 128, 240–7 (1996).

12. Willner, P., Lappas, S., Cheeta, S. & Muscat, R. Reversal of stress-induced anhedonia by the dopamine receptor agonist, pramipexole. Psychopharmacology 115, 454–62 (1994).

13. Jokela, M., Virtanen, M., Batty, G. D. & Kivimäki, M. Inflammation and Specific Symptoms of Depression. JAMA Psychiatry 73, 87 (2016).

14. Felger, J. C. et al. Inflammation is associated with decreased functional connectivity within corticostriatal reward circuitry in depression. Molecular Psychiatry 21, 1358–1365 (2016).

15. Jha, M. K. et al. Can C-reactive protein inform antidepressant medication selection in depressed outpatients? Findings from the CO-MED trial. Psychoneuroendocrinology 78, 105–113 (2017).

16. Barone, P. et al. Pramipexole for the treatment of depressive symptoms in patients with Parkinson's disease: a randomised, double-blind, placebo-controlled trial. The Lancet Neurology 9, 573–580 (2010).

17. Corrigan, M. H., Denahan, A. Q., Wright, C. E., Ragual, R. J. & Evans, D. L. Comparison of pramipexole, fluoxetine, and placebo in patients with major depression. Depression and anxiety 11, 58–65 (2000).

18. Franco-Chaves, J. A. et al. Combining a dopamine agonist and selective serotonin reuptake inhibitor for the treatment of depression: A double-blind, randomized pilot study. Journal of Affective Disorders 149, 319–325 (2013).

19. Cusin, C. et al. A Randomized, Double-Blind, Placebo-Controlled Trial of Pramipexole Augmentation in Treatment-Resistant Major Depressive Disorder. The Journal of Clinical Psychiatry 74, e636–e641 (2013).

20. Fawcett, J. et al. Clinical Experience With High-Dosage Pramipexole in Patients With Treatment-Resistant Depressive Episodes in Unipolar and Bipolar Depression. The American journal of psychiatry 173, 107–11 (2016).

21. Glassman, A. H. Depression and cardiovascular comorbidity. Dialogues in Clinical Neuroscience 9, 9–17 (2007).

22. Otte, C. et al. Major depressive disorder. Nature reviews. Disease primers 2, 16065 (2016).

23. Winer, E. S. et al. Anhedonia predicts suicidal ideation in a large psychiatric inpatient sample. Psychiatry Research 218, 124–128 (2014).

24. Constantinescu, R. Update on the use of pramipexole in the treatment of Parkinson's disease. Neuropsychiatric disease and treatment 4, 337–52 (2008).

25. Aiken, C. B. Pramipexole in psychiatry: a systematic review of the literature. The Journal of clinical psychiatry 68, 1230–6 (2007).

26. Zarate, C. A. et al. Pramipexole for bipolar II depression: a placebo-controlled proof of concept study. Biological Psychiatry 56, 54–60 (2004).

27. Snaith, R. P. et al. A scale for the assessment of hedonic tone the Snaith-Hamilton Pleasure Scale. The British journal of psychiatry : the journal of mental science 167, 99–103 (1995).

28. Thase, M. E. & Rush, A. J. The Journal of clinical psychiatry. The Journal of Clinical Psychiatry vol. 58 ([Physicians Postgraduate Press], 1997).

29. Nakonezny, P. A., Carmody, T. J., Morris, D. W., Kurian, B. T. & Trivedi, M. H. Psychometric evaluation of the Snaith-Hamilton pleasure scale in adult outpatients with major depressive disorder. International clinical psychopharmacology 25, 328–33 (2010).

30. HAMILTON, M. A rating scale for depression. Journal of neurology, neurosurgery, and psychiatry 23, 56–62 (1960).

31. Do not blame the SSRIs: blame the Hamilton Depression Rating Scale. (2021) doi:10.1017/neu.2017.6.

32. Niméus, A., Alsén, M. & Träskman-Bendz, L. The Suicide Assessment Scale: An instrument assessing suicide risk of suicide attempters. European Psychiatry 15, 416–423 (2000).

33. Sheehan, D. V et al. The Mini-International Neuropsychiatric Interview (M.I.N.I.): the development and validation of a structured diagnostic psychiatric interview for DSM-IV and ICD-10. The Journal of clinical psychiatry 59 Suppl 2, 22-33;quiz 34-57 (1998).

34. Rizvi, S. J. et al. Development and validation of the Dimensional Anhedonia Rating Scale (DARS) in a community sample and individuals with major depression. Psychiatry Research 229, 109–119 (2015).

35. Montgomery, S. A. & Asberg, M. A new depression scale designed to be sensitive to change. British Journal of Psychiatry 134, 382–389 (1979).

36. Marin, R. S., Biedrzycki, R. C. & Firinciogullari, S. Reliability and validity of the Apathy Evaluation Scale. Psychiatry research 38, 143–62 (1991).

37. Bastien, C. H., Vallières, A. & Morin, C. M. Validation of the Insomnia Severity Index as an outcome measure for insomnia research. Sleep medicine 2, 297–307 (2001).

38. Spitzer, R. L., Kroenke, K., Williams, J. B. W. & Löwe, B. A Brief Measure for Assessing Generalized Anxiety Disorder. Archives of Internal Medicine 166, 1092 (2006).

39. Lindner, P. et al. The Brunnsviken Brief Quality of Life Scale (BBQ): Development and Psychometric Evaluation. Cognitive behaviour therapy 45, 182–195 (2016).

40. Young, R. C., Biggs, J. T., Ziegler, V. E. & Meyer, D. A. A rating scale for mania: Reliability, validity and sensitivity. British Journal of Psychiatry 133, 429–435 (1978).

41. JA Ferris, H. W. The Canadian problem gambling index. (2001).

42. Weintraub, D. et al. Questionnaire for Impulsive-Compulsive Disorders in Parkinson's Disease–Rating Scale. Movement disorders : official journal of the Movement Disorder Society 27, 242 (2012).

43. Nord, M. et al. Comparison of D 2 dopamine receptor occupancy after oral administration of quetiapine fumarate immediate-release and extended-release formulations in healthy subjects. International Journal of Neuropsychopharmacology 14, 1357–1366 (2011).

44. Cipriani, A. et al. Comparative efficacy and acceptability of 21 antidepressant drugs for the acute treatment of adults with major depressive disorder: a systematic review and network meta-analysis. Lancet (London, England) 391, 1357–1366 (2018).

45. Masson, S. C. & Tejani, A. M. Minimum clinically important differences identified for commonly used depression rating scales. Journal of Clinical Epidemiology vol. 66 805–807 (2013).

46. Maresh, E. L., Allen, J. P. & Coan, J. A. Increased default mode network activity in socially anxious individuals during reward processing. Biology of mood & anxiety disorders 4, 7 (2014).

47. Leucht, S et al. What does **HAMD** mean*? J.Affect. Disord.* **148,** 243-248 (2013).

Rating scales and information about fMRI can be found in a separate appendix attached to the protocol.

27. Changes of the protocol after initial approval

No.	Date	Amendment and Reason	Author and, if applicable, New Version
1	220622	Addition to exclusion criterion no. 12 (12.1.2): “…or other intervention…”	V2.0
2	220622	Addition to screening (13.1) and baseline visits (13.3): Routine laboratory tests are mandatory, but absence of other blood tests does not prevent continued participation.	V2.0
3	220622	Addition to screening visit (13.1): Rescreening is allowed after one month.	V2.0
4	220622	Power calculation and number of participants changed from 60 to 80 (17.1).	V2.0
5	220622	Wording updated regarding interim analysis timing: from “after half of participants have completed the study” to “approximately half of participants” (17.3).	V2.0
6	220622	Clarified definition of remission when further dose escalation is stopped (14.3): CGI-S score of 1 or 2.	V2.0
7	220622	Clarification regarding biobank sample storage (13.9.2): Samples will be stored for a maximum of 15 years.	V2.0
8	220622	Randomization added to Trial implementation table (13).	V2.0
9	220622	Clarification that randomization is performed via RedCap software (14.6).	V2.0
10	220622	Measurement of pramipexole concentration in blood samples added (13.9.1).	V2.0
11	220622	Added information about follow-up study at final visit (13.6).	V2.0
12	220622	Clarified that the activity tracker is CE-marked (13.1).	V2.0
13	220622	Added new research question in synopsis and text regarding follow-up, including description of follow-up study in synopsis.	V2.0
14	220622	Study start updated from September to October.	V2.0
15	220622	Funding adjusted.	V2.0
16	220622	Text on fatigue syndrome added to background; removed as inclusion criterion.	V2.0

17	220622	Clarified that biomarkers to be measured are not limited to dopamine and inflammatory markers, also applies to research question 5.	V2.0
18	220622	Clarified that the activity tracker is CE-marked (10.2).	V2.0
20	220622	Change regarding discontinuation of study participation due to insufficient compliance (13.8).	V2.0
21	220622	Clarification that pramipexole may continue either within follow-up study or with participant's regular physician (outside follow-up study).	V2.0
22	220622	Clarified that screening numbers are assigned during screening visit (13.1).	V2.0
23	220622	Clarification regarding traceability of study medication (14.2.2).	V2.0
24	220622	New procedure for code-breaking (14.8).	V2.0
25	220622	Clarification of definition of outlier measurements (15.2.1 and 15.2.2).	V2.0
26	220622	Clarification that data is collected via eCRF and not on paper (18.1).	V2.0
27	220622	Clarification regarding patient information and obtaining informed consent (21.4).	V2.0
28	220622	Added section on data protection.	V2.0
29	220622	Expanded text regarding assessment of causality for incidents.	V2.0
30	220622	Clarification regarding incident registration (16.5).	V2.0
31	220912	Clarification regarding tapering/continuation of medication after study completion (13.6).	V2.1
32	220912	Clarification regarding statistical analysis and analysis population (17.1 and 17.2).	V2.1
33	230301	Clarification of exclusion criterion 9: "…or severe cardiovascular disease (specifically symptomatic heart failure NYHA class 2 or higher)."	V3.0
34	230301	Clarification of exclusion criterion 12: "…or previous gastric bypass surgery affecting absorption of extended-release tablets in the gastrointestinal tract."	V3.0
35	230301	Changed timing of screening labs from 7 days to 14 days: "referral for lab tests to be performed no later than 14 days prior to baseline…"	V3.0
36	230301	Clarified that baseline must be performed 7–14 days after screening in trial implementation table and section 13.3.	V3.0
37	230301	In section 13.7, added permission to contact relatives if participant cannot be reached unexpectedly: "In case the patient unexpectedly or suddenly cannot be reached,there is an inform consent to contact	V3.0

		the relatives by the study staff (if recorded in the medical record) to ensure no accident or other incident has occurred that could lead to the patient suddenly,undersired finish the treatment and are atrisk of withdrawal symptoms.”	
38	230301	Clarified exclusion criterion 7: “History or strong clinical suspicion of impulse control disorder…”	V3.0
39	230301	Clarified that SUAS and MINI are used to support clinical assessment of diagnosis, suicide risk, and comorbidity in trial implementation table and section 13.1.	V3.0
40	230301	Removed HDRS-6 screening at screening visit (13.1)	V3.0
41	230302	Clarified handling of outlier routine lab values (15.2.1): “Values outside Labmedicin Skåne reference range noted in medical record with comment onhow these may be followed-up. In the event of abnormal ,clinical significant values during the study i.e identified at final visit but not present at baseline are recorded and reported as study adverse events (see 16.5).”	V3.1
42	230302	Sections 10 and 11 updated: recruitment of 40 healthy controls for blood, MRI, and LP.	V3.1
43	230302	15.2.2 clarified: abnormal/unexpected vital signs at screening not considered AE but recorded in journal with follow-up plan (systolic BP >150 or <100).	V3.1
44	230509	15.2.1 clarified: non-significant routine lab results not reported as AE at week 9; clinically significant deviations at study end reported as incidents (16.5).	V3.2
45	230509	Adjusted storage temperature of study product per IMPD instructions: 15–30°C.	V3.2
46	231030	Title page: updated version and date.	V4.0
47	231101	Added background for introduction of cognitive testing (p.23, section 8).	V4.0
48	231101	Added secondary research question 7 and secondary variables 7.	V4.0
49	231102	Updated synopsis with addition of research question 7 and secondary variables 7.	V4.0
50	231102	Added cognitive test forms in section 4 (Abbreviations).	V4.0
51	231102	11. Study design: Added third substudy with neuropsychological and cognitive testing.	V4.0
52	231102	10. Objectives and research questions: Added paragraph on neuropsychological testing and cognitive function.	V4.0

53	231102	Added neuropsychological testing (substudy) under 12. Study population and 13. Study conduct during main study (RCT), including 13.1 Recruitment and screening visits, 13.2 Informed Consent process.	V4.0
54	231107	Added description of neuropsychological testing content under 13.3 Pre-treatment Baseline (7–14 days post-screening).	V4.0
55	231115	Added financial compensation for participants in neuropsychological substudy (21.5).	V4.0
56	231115	Added information regarding storage and archiving of data collected during neuropsychological testing.	V4.0
57	240416	Reordered table 13.0 chronologically; added PRT; clarified AE assessment during telephone follow-ups.	V5.0
58	240416	15.2.1 clarified: only clinically significant values noted in participant journal (per study physician).	V5.0
59	240416	Updated synopsis with addition of PRT.	V5.0
60	240416	Section 10 clarified: healthy controls matched to depressed participants; financial compensation for participation, added PRT test in cognitive assessments for healthy controls. Section 10 and 11	V5.0
61	240416	Sections 10.2, 13.2, 13.3: added PRT as part of neuropsychological/cognitive testing.	V5.0
62	240416	Added PRT appendix under section 27.	V5.0
63	240416	Added new EU trial number; updated data archived to 25 years per CTR regulations.	V5.0
64	240416	Updated number of healthy controls from 40 to 60.	V5.0
65	241010	Updated number of participants after interim analysis from 80 including intention-to-treat to 80 completing study.	V6.0
66	250218	Updated number of randomized participants to 90 to ensure at least 80 complete the study.	V7.0
67	250619	Added analysis of biomarkers related to blood-brain barrier, drug transporters, and study drug concentration.	V8.0

**Protocol versions**		
V1.0	Submitted to EPM	Approved by EPM
V2.0	Submitted to LVM (V1.0 + administrative changes)	Approved by LMV
V2.1	Administrative changes, Sept 2022	
V3.1	Supplementary application EPM	Approved by EPM
V.3.2	Amendment application LVM	Approved by LVM
V.4.0	Ammendement application LVM and EPM	Approved by EPM and LVM
V. 5.0	Amendment application CTIS	Approved
V 6.0	Amendment application CTIS, part 1 and 2	Aprrroved
V.7,0	Amendment application CTIS, part 1	Approved
V. 8.0	Amendment application CTIS, part 1	Approved

New references (page 23) :

Hammar, A. and G. Ardal (2009). "Cognitive functioning in major depression--a summary." Front Hum Neurosci 3: 26.

Hammar, Å., E. H. Ronold and G. Rekkedal (2022). "Cognitive Impairment and Neurocognitive Profiles in Major Depression-A Clinical Perspective." Front Psychiatry 13: 764374.

Adjunctive treatment with Pramipexole for Anhedonia Symptoms of Depression

PRIME-PRAXOL

Statistical Analysis Plan (SAP)

Version: 1.0, dated: 20250603

EudraCT number: 2024-512495-00

Protocol ID: NCT05355337

Protocol version: 6.0, daterad: 20241010

Authors: Daniel Lindqvist, Filip Ventorp, Helene Jacobsson

1 Table of contents

1 Table of contents 2
2 Roles and responsibilities 3
3 Signature 4
4 Introduction 5
5 Objectives and issues 5
6 Study design 5
6.1 Study design 5
6.2 Sample size calculation 5
6.3 Randomization 6
6.4 Interim analysis 6
7 Analysis sets 6
8 Outcome measures 7
8.1 Primary efficacy outcome measures 7
8.2 Secondary efficacy outcome measures 7
8.3 Additional outcomes measures 7
8.4 Safety outcome measure 7
9 Data processing 8
9.1 Missing values 8
9.2 Calculated variables 8
9.3 Transformations 8
10 Statistical analysis 8
10.1 Statistical basis 8
10.2 Effect 8
10.2.1 Primary 8
10.2.2 Secondary 9
10.3 Additional 9
10.4 Safety 9
11 Statistical program 9
12 References 11

2 Roles and responsibilities

Name: Daniel Lindqvist

Role: PI/Sponsor

Organization: Lund University, Region Skåne

Name: Filip Ventorp

Role: co-PI

Organization: Lund University, Region Skåne

Name: Helene Jacobsson

Role: Statistician

Organization: Clinical Studies Sweden, Forum South

3 Signature

We have read this Statistical Analysis Plan carefully and believe that it contains all the necessary information to carry out the analyses of the PRIME-PRAXOL study.

Name: Daniel Lindqvist

Role: PI/Sponsor

Organization: Lund University, Region Skåne

Name: Filip Ventorp

Role: co-PI

Organization: Lund University, Region Skåne

Name: Helene Jacobsson

Role: Statistician

Organization: Clinical Studies Sweden, Forum South

4 Introduction

The majority of depressed patients do not achieve remission with current methods. A common and disabling symptom after treatment is anhedonia – the inability to fell pleasure and reduced "drive". Today, there is no specific and effective treatment method for anhedonia. Pramipexole is a dopamine agonist used in Parkinson's disease and has been shown in several clinical studies to be effective against anhedonia symptoms in this disease. Clinical experience has shown a treatment effect with high-dose pramipexole in treatment-refractory depression, but randomized clinical studies have only been conducted with low-dose pramipexole and have not investigated the specific effects against anhedonia. In a recently completed pilot study from our research group, approximately one third of patients showed a treatment effect on high dose pramipexole.

The purpose of this study is to investigate the effect of add-on pramipexole on anhedonia depression and other symptoms in patients with depressive disorders.

The SAP covers the primary and some of the secondary efficacy variables.

For detailed information, see the Protocol which has been pre-published (*1*)

5 Objectives and issues

The overall objective is to investigate the efficacy and tolerability of add-on pramipexole in anhedonia depression.

Biomarkers have been collected for a subset of the patients to demonstrate target engagement and find treatment predictors.

6 Study design

6.1 Study design

This is a double-blind trial in which a total of 80 patients are randomized to receive either add-on pramipexole or placebo for nine weeks. Study participants undergo optional fMRI examination and lumbar puncture at baseline and at the end of the RCT.

For detailed information, see the pre-published Protocol (*1*).

6.2 Sample size calculation

For the primary question (SHAPS), a linear mixed model with repeated measures (w 3, w 6 and w 9) will be used, where the primary question will be answered with the overall comparison between the

randomized groups. Any group differences at baseline are corrected by including the baseline values as a covariate in the model. The estimated effect size of pramipexole treatment is estimated at 0.27 as this has been reported in other studies of antidepressant drugs. This corresponds to a score difference in MADRS of approximately 4 points which is considered a clear clinical improvement and corresponds to ≥ 4 points on SHAPS. Estimate of the correlation coefficient between repeated measures of MADRS scores is based on material from our previous pilot study (r=0.5). In a power calculation with strength of 80% and α=0.05, 74 research subjects were estimated to be needed. In the original power calculation, the number of dropouts was estimated to be less than 5% (based on the pilot study), whereby we needed to include 80 research subjects in the study (ITT)

After a planned interim analysis, conducted by an external statistician, we estimate that we need to include 80 patients who reach the week 9 visit (end of study).

6.3 Randomization

The randomization process uses a randomization list generated by an independent statistician at Clinical Studies Sweden – Forum South. Blinded study personnel are unaware of the randomization outcome. Research participants are included/randomized consecutively as they are deemed eligible for the study.

6.4 Interim analysis

An interim analysis was planned according to the original protocol. In June of 2024 an independent statistician conducted an interim analysis on the primary outcome and number of dropouts. The result of this interim analysis was that we decided to increase the number of recruited patients to 80 with complete efficacy data through the week 9 visit.

7 Analysis sets

Primary and secondary outcome measures related to the efficacy of pramipexole will be analyzed in the intention-to-treat (ITT)-population, which is all randomized patients with at least one follow-up assessment, i.e. all patients who reached the week 3 assessment.

We will conduct two separate ITT-analyses:

1. In the event of missing measurements at different time points, we will compensate by using the last observation carried forward (LOCF) method.
2. One analyses with no imputed values

One subject (PP02) was randomized, but it was later noted by the external monitor that he had not fulfilled the SHAPS inclusion criteria. Data from this patient will not be included in the statistical

analyses for the primary and efficacy-based secondary outcomes. This decision was made by the external monitor, lead statistician, and the data manager – none of them involved in assessments of study subject.

The safety population is defined as all patients who received the medication, i.e. adverse events will be reported for all patients who received at least one dose of pramipexole or placebo

8 Outcome measures

8.1 Primary efficacy outcome measures

The primary efficacy outcome measure is the absolute change in the SHAPS (*2*) between baseline and week 9.

8.2 Secondary efficacy outcome measures

The secondary efficacy outcome measure are

- Core depression symptoms (HDRS-6 (*3*))
- Anhedonia (DARS (*4*), covering additional anhedonia domains compared to the SHAPS)
- General depressive symptoms (MADRS-S (*5*))
- Sleep disturbances (ISI (*6*))
- Apathy symptoms (AES (*7*))
- Anxiety symptoms (GAD-7 (*8*))
- Quality of life (BBQ (*9*))

8.3 Additional outcomes measures

Additional outcomes are

- accelerometry
- fMRI

8.4 Safety outcome measure

Adverse events

9 Data processing

9.1 Missing values

If clinical efficacy measurement values are missing at different times, this will be compensated for with last-observation-carried-forward (LOCF).

For the mechanistic secondary outcomes (e.g. fMRI), analyses will be performed only on those subjects who have the necessary data available for these specific analyses, meaning that no imputation will be applied.

9.2 Calculated variables

Total score of the SHAPS will be calculated by summarizing each item with a value from 1 to 4 (max score 56). Total score on the other rating scales will be calculated according to the instructions for each scale.

9.3 Transformations

If assumptions regarding normal distribution are not met, variables may be transformed.

10 Statistical analysis

10.1 Statistical basis

Descriptive statistics will be given as mean (SD), median (Q1–Q3) and (Min–Max) for continuous variables. The number of observations will also be given.
The randomization groups will be compared. Two-sided test of statistical significance is to be used, and the chosen significance level is 0.05. No adjustment for multiplicity will be made.

10.2 Effect

10.2.1 Primary

To answer the primary question, a linear mixed model with repeated measures is used with severity of anhedonia symptoms (SHAPS total score) as the dependent variable and time (week), treatment (pramipexole or placebo), and the interaction of time by treatment as independent variables. The

SHAPS total score at baseline will be used as a covariate in the model. In the event of missing measurements at different time points, this will be compensated for with last observation carried forward (LOCF). Normal distribution is expected based on previous studies.

10.2.2 Secondary

The same statistical method and model as above is used for the other secondary outcome measures where there are more than two measurement points

In addition to analyzing total scores as continuous variables, we will also do descriptive analyses of treatment response and remission status. For this purpose, we use established cut-offs. Response according to the SHAPS is defined as an improvement of ≥50% on total score (each item rated from 1-4), and remission as a SHAPS score of ≤2, based on an alternative scoring approach in which each item is rated either 0 or 1 (*10, 11*). Initially, the published protocol defined SHAPS remission as a score of ≤3. However, to maintain consistency with the original Snaith et al. publication *(12)* — the basis of our SHAPS inclusion criteria — we've updated the definition to a score of ≤2. This was done before all data had been collected and before the randomization code was broken.

Response on the MADRS is defined as ≥50% improvement and remission as a score ≤10. Fisher's Exact test will be used to compare dichotomous outcomes.

10.3 Additional

fMRI experimental designs and planned analysis as well as accelerometry analysis are described in the Protocol (*1*).

10.4 Safety

Adverse events will be presented within randomization groups.

11 Statistical program

IBM SPSS Statistics 28 or higher Windows (IBM Corporation, Armonk, NY, USA), SAS Enterprise Guide 8.3 for Windows (SAS Institute Inc., Cary, NC, USA) or R will be used for the statistical analyses. A p-value below 0.05 was considered significant.

12 References

1. J. Lindahl *et al.*, *BMJ Open* **13**, e076900 (2023).
2. P. A. Nakonezny *et al.*, *Int Clin Psychopharmacol* **25**, 328-333 (2010).
3. P. Bech *et al.*, *Acta Psychiatr Scand* **51**, 161-170 (1975).
4. S. J. Rizvi *et al.*, *Psychiatry Res* **229**, 109-119 (2015).
5. S. A. Montgomery *et al.*, *Br J Psychiatry* **134**, 382-389 (1979).
6. C. H. Bastien *et al.*, *Sleep Med* **2**, 297-307 (2001).
7. R. S. Marin *et al.*, *Psychiatry Res* **38**, 143-162 (1991).
8. R. L. Spitzer *et al.*, *Arch Intern Med* **166**, 1092-1097 (2006).
9. P. Lindner *et al.*, *Cogn Behav Ther* **45**, 182-195 (2016).
10. F. Vinckier *et al.*, *Eur Psychiatry* **44**, 1-8 (2017).
11. B. Cao *et al.*, *Front Psychiatry* **10**, 17 (2019).

12. R. P. Snaith *et al.*, *Br J Psychiatry* **167**, 99-103 (1995).

Section/topic	No	CONSORT 2025 checklist item description	Reported on page no.
**Title and abstract**			
Title and structured abstract	1a	Identification as a randomised trial	1, 2
	1b	Structured summary of the trial design, methods, results, and conclusions	2
**Open science**			
Trial registration	2	Name of trial registry, identifying number (with URL) and date of registration	2, 29
Protocol and statistical analysis plan	3	Where the trial protocol and statistical analysis plan can be accessed	29
Data sharing	4	Where and how the individual de-identified participant data (including data dictionary), statistical code and any other materials can be accessed	31
Funding and conflicts of interest	5a	Sources of funding and other support (eg, supply of drugs), and role of funders in the design, conduct, analysis and reporting of the trial	15
	5b	Financial and other conflicts of interest of the manuscript authors	17
**Introduction**			
Background and rationale	6	Scientific background and rationale	3-4
Objectives	7	Specific objectives related to benefits and harms	4
**Methods**			
Patient and public involvement	8	Details of patient or public involvement in the design, conduct and reporting of the trial	N/A
Trial design	9	Description of trial design including type of trial (eg, parallel group, crossover), allocation ratio, and framework (eg, superiority, equivalence, non-inferiority, exploratory)	5, 24
Changes to trial protocol	10	Important changes to the trial after it commenced including any outcomes or analyses that were not prespecified, with reason	See study protocol attached, including summary of amendements
Trial setting	11	Settings (eg, community, hospital) and locations (eg, countries, sites) where the trial was conducted	24
Eligibility criteria	12a	Eligibility criteria for participants	25
	12b	If applicable, eligibility criteria for sites and for individuals delivering the interventions (eg, surgeons, physiotherapists)	25
Intervention and comparator	13	Intervention and comparator with sufficient details to allow replication. If relevant, where additional materials describing the intervention and comparator (eg, intervention manual) can be accessed	24
Outcomes	14	Prespecified primary and secondary outcomes, including the specific measurement variable (eg, systolic blood pressure), analysis metric (eg, change from baseline, final value, time to event), method of aggregation (eg, median, proportion), and time point for each outcome	6-9
Harms	15	How harms were defined and assessed (eg, systematically, non-systematically)	28
Sample size	16a	How sample size was determined, including all assumptions supporting the sample size calculation	29

	16b	Explanation of any interim analyses and stopping guidelines	29
Randomisation:			
Sequence generation	17a	Who generated the random allocation sequence and the method used	24
	17b	Type of randomisation and details of any restriction (eg, stratification, blocking and block size)	24
			**Reported on page no.**
Allocation concealment mechanism	18	Mechanism used to implement the random allocation sequence (eg, central computer/telephone; sequentially numbered, opaque, sealed containers), describing any steps to conceal the sequence until interventions were assigned	24+attached protocol
Implementation	19	Whether the personnel who enrolled and those who assigned participants to the interventions had access to the random allocation sequence	24
Blinding	20a	Who was blinded after assignment to interventions (eg, participants, care providers, outcome assessors, data analysts)	24
	20b	If blinded, how blinding was achieved and description of the similarity of interventions	24+attached protocol
Statistical methods	21a	Statistical methods used to compare groups for primary and secondary outcomes, including harms	29
	21b	Definition of who is included in each analysis (eg, all randomised participants), and in which group	5,7+figure 1
	21c	How missing data were handled in the analysis	10
	21d	Methods for any additional analyses (eg, subgroup and sensitivity analyses), distinguishing prespecified from post hoc	10
**Results**			
Participant flow, including flow diagram	22a	For each group, the numbers of participants who were randomly assigned, received intended intervention, and were analysed for the primary outcome	5-6 + table 1
	22b	For each group, losses and exclusions after randomisation, together with reasons	5 + figure 1
Recruitment	23a	Dates defining the periods of recruitment and follow-up for outcomes of benefits and harms	5+attached protocol
	23b	If relevant, why the trial ended or was stopped	N/A
Intervention and comparator delivery	24a	Intervention and comparator as they were actually administered (eg, where appropriate, who delivered the intervention/comparator, how participants adhered, whether they were delivered as intended (fidelity))	6
	24b	Concomitant care received during the trial for each group	Table 1
Baseline data	25	A table showing baseline demographic and clinical characteristics for each group	Table 1
Numbers analysed, outcomes and estimation	26	For each primary and secondary outcome, by group:<br>● the number of participants included in the analysis<br>● the number of participants with available data at the outcome time point<br>● result for each group, and the estimated effect size and its precision (such as 95% confidence interval)<br>● for binary outcomes, presentation of both absolute and relative effect size	
Harms	27	All harms or unintended events in each group	7
Ancillary analyses	28	Any other analyses performed, including subgroup and sensitivity analyses, distinguishing pre-specified from post hoc	10
**Discussion**			
Interpretation	29	Interpretation consistent with results, balancing benefits and harms, and considering other relevant evidence	12-14
Limitations	30	Trial limitations, addressing sources of potential bias, imprecision, generalisability, and, if relevant, multiplicity of analyses	12-14

Citation: Hopewell S, Chan AW, Collins GS, Hróbjartsson A, Moher D, Schulz KF, et al. CONSORT 2025 Statement: updated guideline for reporting randomised trials. BMJ. 2025; 388:e081123.
https://dx.doi.org/10.1136/bmj-2024-081123
© 2025 Hopewell et al. This is an Open Access article distributed under the terms of the Creative Commons Attribution License (https://creativecommons.org/licenses/by/4.0/), which permits unrestricted use, distribution, and reproduction in any medium, provided the original work is properly cited.

*We strongly recommend reading this statement in conjunction with the CONSORT 2025 Explanation and Elaboration and/or the CONSORT 2025 Expanded Checklist for important clarifications on all the items. We also recommend reading relevant CONSORT extensions. See www.consort-spirit.org.
