# Supplementary material for: Efficacy and target engagement of dopamine agonist pramipexole for anhedonic depression: a randomized placebo-controlled trial
Source: Nat Med. 2026 Jun 12;32(7):2570–8. doi: 10.1038/s41591-026-04465-9 (PMC13375643; doi:10.1038/s41591-026-04465-9)
Supplement: Supplementary file 2 — Reporting Summary [file 41591_2026_4465_MOESM2_ESM.pdf]

nature portfolio

Corresponding author(s): Daniel Lindqvist

Last updated by author(s): May, 7th, 2026

Reporting Summary

Nature Portfolio wishes to improve the reproducibility of the work that we publish. This form provides structure for consistency and transparency in reporting. For further information on Nature Portfolio policies, see our Editorial Policies and the Editorial Policy Checklist.

Statistics

For all statistical analyses, confirm that the following items are present in the figure legend, table legend, main text, or Methods section.

n/a	Confirmed	
☐	☒	The exact sample size (*n*) for each experimental group/condition, given as a discrete number and unit of measurement
☐	☒	A statement on whether measurements were taken from distinct samples or whether the same sample was measured repeatedly
☐	☒	The statistical test(s) used AND whether they are one- or two-sided<br>*Only common tests should be described solely by name; describe more complex techniques in the Methods section.*
☐	☒	A description of all covariates tested
☐	☒	A description of any assumptions or corrections, such as tests of normality and adjustment for multiple comparisons
☐	☒	A full description of the statistical parameters including central tendency (e.g. means) or other basic estimates (e.g. regression coefficient) AND variation (e.g. standard deviation) or associated estimates of uncertainty (e.g. confidence intervals)
☐	☒	For null hypothesis testing, the test statistic (e.g. *F*, *t*, *r*) with confidence intervals, effect sizes, degrees of freedom and *P* value noted<br>*Give P values as exact values whenever suitable.*
☒	☐	For Bayesian analysis, information on the choice of priors and Markov chain Monte Carlo settings
☒	☐	For hierarchical and complex designs, identification of the appropriate level for tests and full reporting of outcomes
☐	☒	Estimates of effect sizes (e.g. Cohen's *d*, Pearson's *r*), indicating how they were calculated

Our web collection on statistics for biologists contains articles on many of the points above.

Software and code

Policy information about availability of computer code

Data collection	Clinical data were collected using REDCap (Research Electronic Data Capture) v15.0.28, which served as the electronic Case Report Form (eCRF) system<br><br>Accelerometry data were collected using GT3X-BT, ActiGraph, Pensacola, USA<br><br>GT3X-BT, ActiGraph, Pensacola, USA. Actilife software version 6.13.4 (ActiGraph LLC), was used for programming of the accelerometers and retrieving raw data from the accelerometers.
Data analysis	IBM SPSS Statistics version 28 and 30 (IBM Corporation, Armonk, NY, USA), SAS Enterprise Guide 8.3 with SAS Proc mixed and R (version 4.5.0) with the nlme (v3.1-168) and emmeans (v 1.11.2-8) packages were used for statistical analysis. Data visualization was performed in R using ggplot2 (v 4.0.3). Physical activity data were collected using ActiGraph GT3X-BT accelerometers and processed using:ActiLife software version 6.13.4R version 4.5.0 with the GGIR package (v3.2.6). G*Power version 3.1.9.7 was used for power analysis (see sample size below).

For manuscripts utilizing custom algorithms or software that are central to the research but not yet described in published literature, software must be made available to editors and reviewers. We strongly encourage code deposition in a community repository (e.g. GitHub). See the Nature Portfolio guidelines for submitting code & software for further information.

Data

Policy information about availability of data

All manuscripts must include a data availability statement. This statement should provide the following information, where applicable:

- Accession codes, unique identifiers, or web links for publicly available datasets
- A description of any restrictions on data availability
- For clinical datasets or third party data, please ensure that the statement adheres to our policy

The datasets generated and analyzed during this study contain sensitive personal information and are protected under the European Union Data Protection Regulation (GDPR). De-identified participant data underlying the the findings of this study will be made available from the corresponding author, subject to approval by the relevant institutional ethics committee and in accordance with GDPR and local data protection regulations. requests will be acknowledged within 2 weeks. Following approval and completion of any required data- sharing agreement, data will be typically made available within 4-8 weeks. The SAP was uploaded at clinicaltrials.gov (NCT05355337) and the study protocol is published alongside the main paper as suppl material. The SAS syntax and R scripts used for the analyses in this study are publicly available under the MIT license at: https://github.com/UnitforBAPP/PRIME-PRAXOL_RCT/

Research involving human participants, their data, or biological material

Policy information about studies with human participants or human data. See also policy information about sex, gender (identity/presentation), and sexual orientation and race, ethnicity and racism.

Reporting on sex and gender	We collected information regarding sex (self-report) and this is reported in demographic tables. Intervention and placebo groups were balanced with regards to sex.<br>Sex was recorded for all participants and is reported descriptively. The study was not designed or powered to detect sex-specific effects, and therefore no formal sex-stratified analyses were performed. Participant sex is included in the source data, enabling disaggregated analyses.
Reporting on race, ethnicity, or other socially relevant groupings	We did not include information about race or ethnicity in the paper. Most of the study participants in our study were of Swedish origin, except three people (Turkish origin, Egyptian origin, and German origin)
Population characteristics	Intervention and placebo groups were balanced with regards to age and previous antidepressant treatments (except for SNRI use). Mean (SD) age was 47.0 (14.3) and 47.4 (13.1) in placebo and pramipexole groups respectively. % female was 43.9% vs46.3%
Recruitment	Participants were recruited via advertisements and clinical referrals, and self-referral was permitted. This recruitment strategy may have introduced selection bias, favoring individuals with sufficient functional capacity and motivation to engage with the study. Consequently, patients with more severe symptoms, particularly those experiencing very severe anhedonia or reduced initiative, may have been underrepresented.
Ethics oversight	The study was approved by the Swedish Ethical Review Authority (reference number 2023-01927-02) and the Swedish Medical Products Agency (EudraCT 2022-001563-26 and 2022-502270-17-00).

Note that full information on the approval of the study protocol must also be provided in the manuscript.

Field-specific reporting

Please select the one below that is the best fit for your research. If you are not sure, read the appropriate sections before making your selection.

☒ Life sciences ☐ Behavioural & social sciences ☐ Ecological, evolutionary & environmental sciences

For a reference copy of the document with all sections, see nature.com/documents/nr-reporting-summary-flat.pdf

Life sciences study design

All studies must disclose on these points even when the disclosure is negative.

Sample size	The sample size was determined based on a power calculation targeting the treatment main effect (three measurements: week 3 to endpoint) in a MMRM under a set of prespecified assumptions using G*Power version 3.1.9.7 (Franz Faul, Universität Kiel, Germany). We estimated an effect size of 0.27 for pramipexole, informed by reports from other antidepressant studies. The correlation coefficient between repeated measures was derived from data in our previous pilot study (r = 0.5). To achieve 80% power with an alpha of 0.05, we initially calculated that 74 participants in total were required. Accounting for an estimated 5% dropout rate based on pilot data, we aimed to recruit 80 participants to begin treatment. A prespecified interim analysis was conducted by an independent statistician after approximately half of the participants had been recruited. The number of dropouts was assessed, and the sample size was recalculated using the observed an updated standard deviation, which resulted in an updated effect size estimate. Based on these results, the recruitment target was increased by five participants. Ultimately, 80 patients with complete efficacy data through week 9 were required.
Data exclusions	Two participants who dropped out and had no evaluations after baseline were excluded from the analysis. Another two participants, who had at least one post-baseline evaluation but dropped out before the week 9 visit, were included in the final analysis ,in line with the modified intention-to-treat approach that was prespecified in the SAP. One participant met the eligibility criteria at screening but not at baseline. Despite this, this subject was randomized but later confirmed by the external monitor to be ineligible for the study and was therefore excluded from the analysis.

Replication	This study is a randomized controlled clinical trial and cannot be reproduced in the traditional sense. However, full transparency regarding the methods and tools used has been provided in the manuscript and in a published protocol.
Randomization	A randomization list was used, and the randomization algorithm was securely managed and known only to the external monitor, Clinical Studies Sweden Forum South.
Blinding	All personnel involved in symptom assessments were blinded. A few unblinded personnel were responsible for placing the right randomized treatment in boxes designated for each patient. The unblinded staff was not involved in symptom assessments.

Reporting for specific materials, systems and methods

We require information from authors about some types of materials, experimental systems and methods used in many studies. Here, indicate whether each material, system or method listed is relevant to your study. If you are not sure if a list item applies to your research, read the appropriate section before selecting a response.

Materials & experimental systems

n/a	Involved in the study
☒	☐ Antibodies
☒	☐ Eukaryotic cell lines
☒	☐ Palaeontology and archaeology
☒	☐ Animals and other organisms
☐	☒ Clinical data
☒	☐ Dual use research of concern
☒	☐ Plants

Methods

n/a	Involved in the study
☒	☐ ChIP-seq
☒	☐ Flow cytometry
☐	☒ MRI-based neuroimaging

Clinical data

Policy information about clinical studies

All manuscripts should comply with the ICMJE guidelines for publication of clinical research and a completed CONSORT checklist must be included with all submissions.

Clinical trial registration	The study was pre-registered at clinicaltrials.gov (ref# NCT05355337 and NCT05825235)
Study protocol	The study protocol has been included in the submission and was previously published.
Data collection	This was an academic single-center trial conducted in Lund, Sweden. Patient enrolment in the RCT began on February 16, 2023, and was completed on April 4, 2025
Outcomes	Primary Outcome: Will treatment with pramipexole at the highest tolerable dose (maximum 3.15 mg base, without intolerable adverse reactions) reduce anhedonia symptoms over a nine-week period compared to placebo? Assessed by total SHAPS scores at baseline, week 3, week 6, and week 9 (endpoint).<br><br>Clinical secondary outcomes included change in HDRS-6, MADRS-S, DARS, AES-S, GAD-7, ISi, BBQ<br><br>Safety and tolerability of pramipexole were secondary outcomes. This was assessed by adverse events and reactions in the pramipexole and placebo groups.<br><br>Long-term efficacy and tolerability were also secondary outcomes, we report data from open-label extension phase (up to 6 months of treatment after RCT).<br><br>Exploratory outcomes included:<br><br>Treatment-associated change in daily physical activity, assessed by accelerometry<br><br>Treatment-associated change in Reward-related BOLD activity response in the ventral striatum (fMRI)<br><br>Treatment-associated changes in Cognitive assessments and the Probabilistic Reward Task (PRT)<br><br>Treatment-associated changes in dopaminergic and inflammatory biomarkers<br><br>Additional outcome measures were prespecified in the protocol but are not reported in this manuscript.

Plants

Seed stocks

Report on the source of all seed stocks or other plant material used. If applicable, state the seed stock centre and catalogue number. If plant specimens were collected from the field, describe the collection location, date and sampling procedures.

Novel plant genotypes

Describe the methods by which all novel plant genotypes were produced. This includes those generated by transgenic approaches, gene editing, chemical/radiation-based mutagenesis and hybridization. For transgenic lines, describe the transformation method, the number of independent lines analyzed and the generation upon which experiments were performed. For gene-edited lines, describe the editor used, the endogenous sequence targeted for editing, the targeting guide RNA sequence (if applicable) and how the editor was applied.

Authentication

Describe any authentication procedures for each seed stock used or novel genotype generated. Describe any experiments used to assess the effect of a mutation and, where applicable, how potential secondary effects (e.g. second site T-DNA insertions, mosiacism, off-target gene editing) were examined.

Magnetic resonance imaging

Experimental design

Design type

Task-based design using the Monetary Incentive Delay (MID) task, event-related design

Design specifications

The task was a simplified version of the MID containing only two conditions, high reward and low reward (and no conditions evalutaion loss). Each trial began with a cue (red or blue circle) signifying whether the trial was associated with a high reward (0.5$) or a low reward (0.01$), subsequently a target appeared (a yellow star) and the participant was instructed to press a response button as quckly as possible. If the response was fast enough the participant recieved a the reward associated with that trial type. This outcome was presented on screen (e.g. "You won 0.5$"). If the response was not fast enough this outcome was presented on screen ("Too slow"). The cut-off for response-time was based on the subjects previous responses and continously adjusted to achieve success rate of 75-80%. The task was performed once before treatment and once after treatment and delivered in one single run on each session. Each session included a total of 60 trials (30 high reward and 30 low reward). Presentation times were as follows; inter-trial intervals varied between 4-7 sec, cues were presented for 2 sec, the gap between cue-offset and target onset varied between 0.4-0.6 sec, the target was presented for 1.5 sec irrespective of response-time, the gap between target offset and feedback onset varied between 1-3 sec and the outcome was shown for 2 sec. The entire task took approximately 13 minutes in total each session. Only data for the anticipation phase (i.e. cue signalling high or low reward is presented in the current publication).

Behavioral performance measures

Response times and success-rate was recorded, but not analyzed or presented in the current publication.

Acquisition

Imaging type(s)

Functional magnetic resonance imaging (fMRI)

Field strength

7 Tesla

Sequence & imaging parameters

Data were acquired using a 7T whole body MR scanner (Philips Achieva, Philips Medical Systems, Best, The Netherlands) with a 32-channel head coil. BOLD fMRI was collected using a simultaneous multi-slice echo-planar imaging (SMS-EPI) sequence with parameters (TR=1500 ms, TE=25 ms, flip angle 55°, voxel size=2.0×2.0×2.0 mm3, slice gap=0.2 mm, slices=50, multi-slice acceleration factor=2, SENSE acceleration factor=3).

Area of acquisition

Whole-brain

Diffusion MRI ☐ Used ☒ Not used

Preprocessing

Preprocessing software

Preprocessing was performed using fMRIprep 23.1.4 with fieldmaps for distortion correction and freesurfer surface reconstruction disabled. Smoothing was performed using SPM12 with a 6x6x6 mm smoothing kernel.

Normalization

The BOLD time-series were resampled into standard space, generating a preprocessed BOLD run in MNI152NLin2009cAsym space. First, a reference volume and its skull-stripped version were generated using a custom methodology of fMRIPrep. All resamplings can be performed with a single interpolation step by composing all the pertinent transformations (i.e. head-motion transform matrices, susceptibility distortion correction when available, and co-registrations to anatomical and output spaces). Gridded (volumetric) resamplings were performed using antsApplyTransforms (ANTs), configured with Lanczos interpolation to minimize the smoothing effects of other kernels (Lanczos 1964). Non-gridded (surface) resamplings were performed using mri_vol2surf (FreeSurfer).

Normalization template

MNI152

Noise and artifact removal

Movement parameters from fmriprep were entered as parameters in first level models.

Volume censoring | No volume censoring was performed.

Statistical modeling & inference

Model type and settings

First level analysis: For each participant, functional images from each session was entered into a single first level model, using a univariate approach. This model included onsets and durations of the high-reward and low-reward cues, the onsets and durations of target presentation (irrespective of condition), the onset and duration of the time period between target off-set and outcome onset (irrespective of condition), as well as onsets and durations for the high-reward, low-reward and no-reward outcomes respectively. Each of these regressors were convolved with the canonical haemodynamic response-function in SPM12. Also, six rotational parameters where added to the model as covariates of no interest too account for movement related artefacts. Group analysis was performed on activity statistics extracted from an specified ROI, thus our analysis approach did not include second-level modeling within the imaging analysis pipeline. Our main contrast of interest was high reward vs low reward during the anticipation phase (i.e. when the participant is viewing the cue signalling whether the current trial is high reward och low reward).

Effect(s) tested

For each participant and session, the contrast for high reward vs low reward during the anticipation phase was used and the average activation statistic was extracted from an region of interest (ROI) encompassing the ventral striatum bilaterally. This ROI was created using the Accumbens region from the Harvard-Oxford subcortical atlas (none-thresholded, i.e. using no cut-off with respect to probablistic classification). When extracting activation statistics no statistical threshold was used, i.e. the statistic for each participant represent the average value of all the voxels in ROI. Then we performed group analysis on these values for the entire sample, evaluating time by treatment effects using a mixed model appraoch.

Specify type of analysis: ☐ Whole brain ☒ ROI-based ☐ Both

Anatomical location(s)

The region of interest (ROI) encompasses the ventral striatum bilaterally. This ROI was created using the Accumbens region from the Harvard-Oxford subcortical atlas (none-thresholded, i.e. using no cut-off with respect to probablistic classification).

Statistic type for inference

(See Eklund et al. 2016)

Not applicable. Analysis was performed on a single value exctracted from an anatomically defined ROI, and voxel-level or cluster level statistics are not included in the analysis.

Correction

Since analysis was performed on a single extracted value, no correction for multiple comparisons was applied.

Models & analysis

n/a	Involved in the study
☒	☐ Functional and/or effective connectivity
☒	☐ Graph analysis
☒	☐ Multivariate modeling or predictive analysis
