# Supplementary material for: Efficacy and target engagement of dopamine agonist pramipexole for anhedonic depression: a randomized placebo-controlled trial
Source: Nat Med. 2026 Jun 12;32(7):2570–8. doi: 10.1038/s41591-026-04465-9 (PMC13375643; doi:10.1038/s41591-026-04465-9)
Supplement: Supplementary file 3 — Peer Review File [file 41591_2026_4465_MOESM3_ESM.pdf]

nature portfolio

Peer Review File

Efficacy and target engagement of dopamine agonist pramipexole for anhedonic depression: a randomized placebo controlled trial

Corresponding Author: Dr Daniel Lindqvist

This file contains all reviewer reports in order by version, followed by all author rebuttals in order by version.

Version 0:

Reviewer comments:

Reviewer #1

(Remarks to the Author)
Summary of the key results
This randomized controlled trial compared pramipexole with placebo over nine weeks in patients with major depressive disorder, bipolar disorder, or dysthymia who exhibited pronounced anhedonia. Pramipexole significantly improved anhedonia (SHAPS) relative to placebo, the pre-specified primary outcome. Broader depressive symptom scales (MADRS-S and HDRS-6) showed non-significant between-group differences at the week-9 endpoint. Side effects aligned with pramipexole's known profile and did not increase dropout rates. fMRI and accelerometry analyses supported target engagement in motivational and reward systems.

Originality and significance
The study's originality lies in its symptom-based, transdiagnostic design, recruiting patients with pronounced anhedonia rather than based on global depression severity. This distinguishes it from recent work such as Browning et al. (Lancet Psychiatry, 2025), which focused on treatment-resistant depression. The integration of neuroimaging and mobile-sensing data adds mechanistic depth and strengthens the link between dopaminergic modulation and motivational improvement.

Data & methodology
The trial is well conceived but weakened by analytic deviations and incomplete reporting :
- Two participants without post-baseline data and one ineligible but randomized subject were excluded from the primary analysis. Such exclusions can bias estimates and undermine randomization. A proper ITT analysis should retain all randomized participants, with missing outcomes handled statistically rather than by exclusion.
- An interim analysis reportedly informed an updated power calculation, but the manuscript does not provide details on its results or whether these affected methodological decisions (e.g., inclusion/exclusion criteria, analytical strategy).
- The use of last observation carried forward (LOCF) in conjunction with MMRM is statistically inconsistent. MMRM already handles missing data under the missing-at-random (MAR) assumption using maximum likelihood estimation. LOCF violates that assumption, biases variance estimates, and can distort treatment effects. The analysis should rely solely on MMRM or use multiple or reference-based imputation methods for sensitivity checks
- Twelve preregistered accelerometry outcomes were listed, yet only four are reported. Unless clearly justified (e.g., data loss or redundancy), this constitutes selective outcome reporting. Either all preregistered outcomes should appear in the supplementary material, or the omission should be transparently explained. Without correction for multiple testing, secondary and exploratory results must be explicitly labeled as exploratory to avoid overstating significance.
- Methodologically, the trial would benefit from additional detail on randomization, allocation concealment, and blinding procedures (per CONSORT 2025). The manuscript does not specify sequence generation, block sizes, or whether raters were involved in dose adjustments, which could compromise blinding given pramipexole's side-effect profile. Adverse-event assessment procedures are also insufficiently described.

Appropriate use of statistics and treatment of uncertainties
The statistical framework is partially sound but inconsistently applied.
- For the secondary outcomes, reporting both non-significant endpoint differences and significant "main effects across time" without clarifying which estimand was primary risks post-hoc reinterpretation.

- Multiplicity is another concern. Multiple endpoints (SHAPS, MADRS-S, HDRS-6, GAD-7, ISI, AES-S, BBQ, 12 accelerometry variables, fMRI outcomes) and multiple visits (weeks 3, 6, 9) create numerous testing opportunities. The manuscript does not describe any adjustment for family-wise error or false-discovery rate. Without such correction, reported p-values must be considered nominal.

Conclusions: robustness, validity, reliability
The finding that pramipexole improves anhedonia is supported by the primary SHAPS analysis but should be interpreted with caution. The lack of consistent effects on standard depression scales weaken claims of overall antidepressant efficacy. Assertions that pramipexole may be suitable for less-resistant depression are speculative without corresponding subgroup or sensitivity analyses. Long-term efficacy and tolerability remain untested beyond the nine-week period, limiting validity. Nevertheless, the consistent mechanistic evidence from fMRI and activity data provides convergent support for dopaminergic modulation of motivation, enhancing the biological plausibility of the findings.

Suggested improvements
1. Re-run all primary and secondary analyses in a full ITT dataset including all randomized participants.
2. Eliminate LOCF imputation; rely on MMRM or modern multiple imputation techniques.
3. Provide complete reporting of preregistered accelerometry endpoints (e.g., as supplementary data) and clarify how multiplicity was addressed.
4. Explicitly define the statistical hierarchy: which contrast (week 9 or averaged effect) is primary, which outcomes are secondary, and how multiplicity was controlled.
5. Add full CONSORT 2025-required details: randomization method, blinding maintenance, AE definitions, and any protocol changes.
6. Discuss the potential bias introduced by the interim analysis and describe its influence on subsequent study conduct.
7. Temper the interpretation of efficacy in less-resistant or dysthymic patients unless additional analyses support this claim.
8. Add a clearer discussion of the lack of significant effects on MADRS-S and HDRS-6 and the implications for overall clinical benefit, especially in contrast to Browning et al. (Lancet Psychiatry, 2025), who demonstrated antidepressant efficacy in treatment-resistant depression.

References
Prior work is cited appropriately.

Clarity and context
The manuscript is well written and visually appealing, but some elements of transparency are missing.
- The flow diagram should follow CONSORT standards, listing all randomized participants, discontinuations, and analysis sets.
- Statistical group comparisons at baseline are inappropriate in randomized trials, as any differences arise by chance. Baseline data should be reported descriptively only.
- Present absolute values alongside percentages in the baseline table and include clinically relevant variables (duration of current episode, concomitant psychotherapy).
- The abstract and conclusions should more accurately reflect that the primary improvement was limited to anhedonia, with broader depressive outcomes not reaching statistical significance.

Overall, this is a well-executed and conceptually important trial with strong mechanistic evidence. Addressing the statistical and reporting issues noted above would substantially strengthen its credibility and impact.

Congratulations to the authors for this ambitious and innovative study, and best wishes from Munich,
[redacted]

Reviewer #3

(Remarks to the Author)
A. Summary of Key Results
Participants treated with pramipexole exhibited a significantly greater reduction in anhedonic symptoms compared with those receiving placebo. Consistent benefits were also observed across secondary measures assessing anhedonia and apathy. Objective accelerometry data corroborated these effects, showing increased physical activity among individuals in the pramipexole group. Functional imaging further demonstrated enhanced reward-related activation in the ventral striatum, which was associated with reductions in anhedonic symptom severity. Overall, pramipexole was well tolerated, with low attrition and no serious adverse events reported.

B. Originality
The study builds on previous clinical and observational evidence by offering new mechanistic insights into pramipexole's antidepressant effects. It demonstrates that pramipexole engages the brain's reward system while also being the first antidepressant RCT to include objective measures of physical activity, showing that the drug alleviates anhedonia both subjectively and through measurable increases in daily activity.
C. Data & methodology: validity of approach, quality of data, quality of presentation
Mixed-model analyses demonstrated that pramipexole led to significantly greater improvement in anhedonic symptoms compared with placebo, with consistent effects across all time points and robustness in completer analyses. Secondary outcomes showed notable reductions in anhedonia and apathy, particularly in motivational domains, with a modest overall

benefit on depressive symptoms but no significant effects on anxiety, insomnia, or quality of life. Accelerometry data indicated increased light physical activity in the pramipexole group, suggesting enhanced behavioral engagement independent of self-reported anhedonia. Neuroimaging results revealed preserved ventral striatal activation during reward anticipation in the pramipexole group, consistent with dopaminergic modulation of reward processing. The treatment was well tolerated, with no serious adverse events; side effects such as sleep disturbance, nausea, and dizziness were more common but manageable, and no participants discontinued due to impulse-control symptoms.
D. Appropriate use of statistics and treatment of uncertainties
Mixed-effects models for longitudinal designs and paired t-test for pre-post comparison are often used in the literature. Though mixed-effects model does not require imputation of missing values, however proper imputation of missing values are always recommended, which the article did.
E. Conclusions: robustness, validity, reliability
This article demonstrated with small samples that pramipexole is an effective and well-tolerated augmentation treatment for anhedonia in mood disorders, offering both clinical and mechanistic evidence of efficacy. By targeting patients with pronounced anhedonia, the trial confirmed that pramipexole alleviates motivational deficits, enhances physical activity, and preserves ventral striatal activation associated with reward processing. The study extends prior findings by incorporating objective measures such as accelerometry and fMRI, strengthening the evidence for dopaminergic modulation as a mechanism of action. Pramipexole showed benefits across depressive subtypes—including MDD, dysthymia, and bipolar depression—supporting its use as a transdiagnostic, symptom-focused treatment. The drug was generally well tolerated, with mostly mild and manageable side effects and a low dropout rate. Together, these results highlight pramipexole as a safe, efficacious, and mechanistically targeted therapy for anhedonic depression, and illustrate the value of multimodal, precision-based approaches in antidepressant research. However, the study sample size was small, hence in order to get a definite usable result more studies with large samples are required.
F. Suggested improvements
The main finding of the study is that, at the population level, patients randomized to the pramipexole intervention showed greater improvement than those receiving placebo. However, several important questions remain unanswered: Which patients within the intervention group derived the most benefit—was it a general effect or limited to those with specific characteristics? What proportion of patients experienced meaningful symptom improvement? And, critically, can we identify the subgroup of individuals most likely to respond favorably to pramipexole?
The neuroimaging component of the study is at the rudimentary level, does not give a deep understanding of the mediation or causal mediation effects of the networks.
G. References: appropriate credit to previous work
References are appropriately cited.
H. Clarity and context
The article is well written.

Reviewer #4

(Remarks to the Author)
This article reports on a robust, methodologically sophisticated, and clinically significant randomized controlled trial (RCT). It provides compelling evidence that pramipexole is an effective and well-tolerated augmentation therapy for anhedonia across mood disorders. The study's major strength lies in its multi-modal assessment strategy, which moves beyond subjective reports to include objective behavioral (accelerometry) and neurobiological (fMRI) measures of target engagement.
Technical Strengths
1. The study is based on a precise and mechanistically-grounded hypothesis. It goes beyond a generic "depression" diagnosis to target a specific symptom cluster (anhedonia) with a drug (a dopamine D2/D3 receptor agonist) that directly engages the hypothesized underlying neurobiology (mesolimbic dopamine pathway dysfunction). This aligns well with the RDoC framework and may prospectively support precision psychiatry.
2. The entire study is based on a rigorous transdiagnostic design and shows that the treatment effect is linked to the symptom (anhedonia) rather than the diagnostic label. This may increase the generalizability and clinical relevance of the findings.
3. The authors provide a multi-modal outcome assessment, which may be the study's most significant strength. The primary outcome (SHAPS) was specific to anhedonia, and secondary scales (DARS, AES-S) provided a nuanced view of motivational and consummatory pleasure. The use of accelerometry is certainly an innovation in antidepressant trials. It provides an objective, ecologically valid measure of real-world behavior change (increased light physical activity), which is a direct behavioral correlate of improved motivation and energy. The finding that this objective measure aligned with subjective reports strongly supports the clinical efficacy of pramipexole. The use of neuroimaging data (7-Tesla fMRI) with the Monetary Incentive Delay (MID) task is also an interesting twist. The high magnetic field strength provides high signal-to-noise ratio for imaging small subcortical structures like the ventral striatum. Demonstrating a significant between-group difference in reward-related activation provides direct evidence of target engagement in the brain's reward circuitry.
4. The core statistical methodology is based on Mixed-Model Repeated Measures (MMRM), a gold standard for analyzing longitudinal clinical trial data, and uses all available data points and handles missing data appropriately. The Hedge's g effect sizes allow for a clear interpretation of the magnitude of the effects and the sensitivity analyses with/without Last

Observation Carried Forward (LOCF).
A remarkably low dropout rate of 6% in a 9-week trial, with no serious adverse events attests to the feasibility and acceptability of this treatment regimen in clinical practice; the slow titration schedule likely contributed to this.

Potential Study Limitations
1. Partial Unblinding in the Placebo Group: This is the most significant methodological weakness. The fact that 66.7% of the placebo group correctly guessed their allocation (p=0.053) introduces a potential for bias, particularly for subjective self-report measures like SHAPS, DARS, and AES-S. Participants knowing they are on placebo may experience disappointment, amplifying their perception of no improvement (a nocebo effect). However, this weakness is substantially mitigated by the positive findings on the objective accelerometry data, which is less susceptible to expectation bias.
The effect sizes are modest. The primary outcome effect size (Hedge's g=0.63) is marginal, which is not atypical for psychopharmacology trials but may suggest the effect is not overwhelming.
The effect on the fMRI outcome was statistically significant but small (g=0.32) and the correlation between clinical improvement and neural change within the pramipexole group shows only a trend (p=0.06). This indicates that while pramipexole engages the ventral striatum on a group level, the link between this engagement and individual symptom improvement is less robust and requires further investigation in larger samples.
While the overall intention-to-treat sample (n=82) is reasonable, the sample sizes for the accelerometry (n=61) and especially the fMRI (n=48) sub-studies are smaller. This reduces the power of these analyses and increases the risk of Type II errors (missing real effects), see the statistical-power comment at the end.
Patients remained on their existing antidepressants/mood stabilizers, which is rational for an augmentation study, but introduces (drug-interaction) variability. The authors did acknowledge a significant baseline imbalance in SNRI use, as a potential confounder.
The 9-week duration establishes efficacy but tells less about the potential long-term durability of the response, the need for continued treatment, or the potential for tolerance or long-term side effects.
I believe the study demonstrates that pramipexole improves motivational anhedonia, but its effect on consummatory anhedonia is less clear.

A specific technical comment: Please double check, and explicate in the appendix, the exact details of the study power calculations. I used Lu, Luo, & Chen (2008) methodology (implemented in R's longpower package), to verify the MMRM power analysis for this study's longitudinal designs. I am getting somewhat different results, a sample-size estimate of 256 (far exceeding the reported sample-size of n=80). If IBM SPSS was used for power calculations, perhaps try to cross-check with R.

Version 2:

Reviewer comments:

Reviewer #1

(Remarks to the Author)
The authors have substantially improved the reporting of methods and results and now offer a more balanced discussion of their primary and secondary outcome findings. I welcome the additional inclusion of follow-up data, even if open-label and thus preliminary in nature. The authors have adequately addressed my previous concerns, and I recommend acceptance for publication.

Reviewer #3

(Remarks to the Author)
A. Summary of the key results
The primary analysis is conducted using mixed-model repeated measures (MMRM) in the modified intention-to-treat sample with baseline adjustment, which is an appropriate framework for longitudinal clinical trial data. The reported treatment effect on SHAPS scores at week 9 (mean difference −4.04; 95% CI −6.89 to −1.18; p=0.006; Hedges' g ≈ 0.62) indicates a statistically detectable improvement associated with pramipexole during the randomized phase. Sensitivity analysis using last-observation-carried-forward produced similar estimates. Baseline SHAPS values were broadly comparable between treatment groups, and estimated marginal mean comparisons favored pramipexole at the evaluated timepoints.
B. Originality and significance: if not novel, please include reference
The trial involved a relatively small sample (about 85 participants), which may limit statistical power and precision of effect estimates. The selected population with severe anhedonia may restrict generalizability to broader depression populations. Mechanistic conclusions remain indirect because outcomes are primarily symptom based. In addition, the open label extension lacks a control group, baseline imbalance in concomitant medication could introduce confounding, and sensitivity analyses relied on last observation carried forward methods. Prior randomized placebo-controlled trials provide convergent evidence that pramipexole may have antidepressant efficacy in bipolar and treatment-resistant depression, but direct replication of the present anhedonia-focused trial appears limited.
C. Data & methodology: validity of approach, quality of data, quality of presentation
The revised manuscript provides more detailed experimental documentation, including demographic descriptions of participants who discontinued early and reporting of the open-label extension phase. It extends the scope by describing an open-label extension study and reporting how many participants enrolled and completed the six-month follow-up. It presents statistical results such as treatment effect estimates, p-values, effect sizes (Hedge's g), and correlations between neural

activation and symptom improvement.
D. Appropriate use of statistics and treatment of uncertainties
The revised manuscript is concise and focused. It directly reports the study outcomes and includes numerical estimates such as effect sizes, p-values, and sample sizes. This makes it clear in communicating the key findings of the study. It interprets the study findings and concludes that pramipexole is an efficacious and safe add-on treatment for anhedonia in mood disorders. It links clinical improvement to changes in reward-related neural activity.
E. Conclusions: robustness, validity, reliability
Several considerations should be noted when interpreting these findings. Although response and remission rates were numerically higher in the pramipexole group, these differences were not statistically significant and therefore provide weaker support for treatment benefit than the continuous SHAPS analysis. In addition, improvement observed during the open-label extension phase was reported descriptively and without a concurrent control group, which limits causal interpretation of changes during this period. The regression analysis identifying predictors of symptom change was exploratory in nature and should be interpreted accordingly. Finally, sensitivity analyses relied on last-observation-carried-forward imputation for missing data, and while the results were similar to the primary analysis, this approach represents only one possible strategy for handling missing outcomes.
F. Clarity and context: lucidity of abstract/summary, appropriateness of abstract, introduction and conclusions
This revised manuscript primarily summarizes empirical findings from a randomized controlled trial evaluating the dopamine agonist pramipexole for the treatment of anhedonia in mood disorders. It provides the background and theoretical rationale motivating the study. The revised manuscript is concise and focused. It directly reports the study outcomes and includes numerical estimates such as effect sizes, p-values, and sample sizes. This makes it clear in communicating the key findings of the study. It contributes some empirical evidence derived from the clinical trial. It presents treatment effects, behavioral outcomes, neuroimaging findings, and safety information.
G. Overall Assessment
The revised manuscript presents the quantitative results of the study. It provides a broader and more detailed clinical narrative that includes screening context and long-term follow-up through an open-label extension study. The study established the conceptual and biological rationale for the investigation with a limited sample. The introduction defines the research question, while the results section provides the evidence addressing that question. Small sample concerns limit the findings of the results.

Reviewer #4

(Remarks to the Author)
The revised manuscript provides a more clear and direct picture of the study design, research findings, and offers appropriate interpretations. Thank you for seriously reviewing and addressing the prior recommendations.

I really like the addition of the tracking from 9-weeks up to 5 months. Here is a small remaining question related to the additional data:

* Figure 2. Longitudinal changes from baseline in Snaith–Hamilton Pleasure Scale (SHAPS) scores and
231 light physical activity (LPA) levels: Please cross-check the graph, there appears to be a conflict with the x-axis (e.g., Snaith–Hamilton Pleasure Scale (SHAPS) scores at week 9 can;t be prior to months 1 and 2). Is this just a labeling issue, or a problem with the result presentation?
Also, for LPA, bottom graph), same issue and apparently no difference between pramipexole (purple line) or placebo (grey line) between week 10 and month 5? Please clarify.

Open Access This Peer Review File is licensed under a Creative Commons Attribution 4.0 International License, which permits use, sharing, adaptation, distribution and reproduction in any medium or format, as long as you give appropriate credit to the original author(s) and the source, provide a link to the Creative Commons license, and indicate if changes were made.
In cases where reviewers are anonymous, credit should be given to 'Anonymous Referee' and the source.
The images or other third party material in this Peer Review File are included in the article's Creative Commons license, unless indicated otherwise in a credit line to the material. If material is not included in the article's Creative Commons license and your intended use is not permitted by statutory regulation or exceeds the permitted use, you will need to obtain permission directly from the copyright holder.
To view a copy of this license, visit https://creativecommons.org/licenses/by/4.0/

Comments from the Editors

While the referees find your work of some interest, we were ultimately not convinced this work was sufficiently clinically impactful for us to consider further. Most notably we were concerned about the short follow-up (9 weeks), the small sample size and possible concerns of blinding integrity. We compared the current work to a piece recently published, https://www.thelancet.com/journals/lanpsy/article/PIIS2215-0366(25)00194-4/fulltext, which followed participants for 48 weeks and was associated with adverse effects.

Reply from authors

We thank the editor and reviewers for their thoughtful consideration of our manuscript and appreciate the opportunity to clarify how our study addresses clinically meaningful questions.

1) Follow-up duration

We acknowledge the limitation of the 9-week follow-up period. To address this, we have recently completed a 6-month open-label extension in the RCT cohort to assess the durability of efficacy and tolerability. In addition, we have accelerometry data for the extension phase.

In a revision, we would include these long-term outcomes (efficacy, safety endpoints & accelerometry data) as an extension analysis, which we believe will substantially strengthen the clinical impact. Preliminary analyses suggest that these data are very promising, with several patients achieving stable clinical remission and robust evidence that pramipexole has a positive effect on physical activity over time.

2) Sample size

-We acknowledge that the sample size is modest; however, it is comparable to prior mechanistic and experimental therapeutics RCTs in high-impact journals (e.g., Krystal et al., 2020, Nature Medicine) and was powered a priori to detect a clinically meaningful effect, with prespecified outcomes and analyses. Importantly, this is an academically independent study, which supports an unbiased evaluation of both clinical and mechanistic outcomes. The use of pre-registration of analyses and primary/secondary outcome variables does not only maximize rigor but is also an improvement from prior efforts in related areas (e.g., Krystal et al., 2020).

-The study integrates clinical efficacy, mechanistic readouts, and objective measures including actigraphy/accelerometry, fMRI and other readouts which to our knowledge is unique in psychiatric RCTs. This multimodal approach provides converging evidence from subjective and objective outcomes enhancing interpretability despite the sample size.

3) Blinding integrity

-Very few psychiatric RCTs assess blinding integrity, despite growing consensus that they should. This is a particular concern in psychedelic trials, where effective blinding is often virtually impossible and the validity of findings has accordingly been questioned. In contrast, we prospectively assessed and transparently reported blinding in our study. Although there was a trend toward reduced blinding in the placebo arm, guess accuracy in the active arm did not exceed chance to an extent that could plausibly explain the primary results, even at higher pramipexole doses.

-The inclusion of objective outcome measures which are less vulnerable to expectancy effects, further mitigates concerns about functional unblinding. In addition, our positive target-engagement findings (fMRI) provide convergent evidence that the observed clinical effects reflect genuine pharmacological action rather than artefacts of compromised blinding.

-To address this more fully, we have added a more detailed analysis of placebo versus active-treatment guesses to clarify the reasons underlying correct guesses. For details, please see our response to Reviewer #4 below.

<u>Reviewers' Comment:</u>

Reviewer #1 (Remarks to the Author): Summary of the key results

This randomized controlled trial compared pramipexole with placebo over nine weeks in patients with major depressive disorder, bipolar disorder, or dysthymia who exhibited pronounced anhedonia. Pramipexole significantly improved anhedonia (SHAPS) relative to placebo, the pre-specified primary outcome. Broader depressive symptom scales (MADRS-S and HDRS-6) showed non-significant between-group differences at the week-9 endpoint. Side effects aligned with pramipexole's known profile and did not increase dropout rates. fMRI and accelerometry analyses supported target engagement in motivational and reward systems.

Originality and significance

The study's originality lies in its symptom-based, transdiagnostic design, recruiting patients with pronounced anhedonia rather than based on global depression severity. This distinguishes it from recent work such as Browning et al. (Lancet Psychiatry, 2025), which focused on treatment-resistant depression. The integration of neuroimaging and mobile-sensing data adds mechanistic depth and strengthens the link between dopaminergic modulation and motivational improvement.

Data & methodology

The trial is well conceived but weakened by analytic deviations and incomplete reporting :

Reviewers' Comment:

Two participants without post-baseline data and one ineligible but randomized subject were excluded from the primary analysis. Such exclusions can bias estimates and undermine randomization. A proper ITT analysis should retain all randomized participants, with missing outcomes handled statistically rather than by exclusion.

Reply from authors

-We appreciate this important point. In our Statistical Analysis Plan, the ITT population was defined a priori as:

"Primary and secondary outcome measures related to the efficacy of pramipexole will be analyzed in the intention-to-treat (ITT) population, which is all randomized patients with at least one follow-up assessment, i.e. all patients who reached the week 3 assessment."

Accordingly, two participants without any post baseline data and one randomized but ineligible subject were excluded from the primary efficacy analyses, in line with this pre specified definition. We agree that, in principle, all randomized participants should be retained in an ITT framework. In our case, the two participants without any post-baseline data are included in the CONSORT flow and baseline summaries, but they contribute no information to the primary endpoint because they have no follow-up assessments from which a change score could be calculated.

We will make this handling of missing data more explicit in the Methods and will clarify that additional analyses including these patients with purely imputed endpoints would not be informative.

We also acknowledge that the exclusion of a randomized but ineligible participant can, in theory, introduce bias. However, this individual should not have been randomized according to the protocol and was removed immediately once the ineligibility was detected. We will clarify this in the revised manuscript and will present all ITT and per-protocol numbers transparently so that readers can fully assess the impact of this deviation.

Reviewers' Comment:

An interim analysis reportedly informed an updated power calculation, but the manuscript does not provide details on its results or whether these affected methodological decisions (e.g., inclusion/exclusion criteria, analytical strategy).

Reply from authors

The pre-planned interim analysis did not influence any methodological decisions other than a modest increase in the recruitment target by five participants, as described in the Methods section. No changes were made to inclusion/exclusion criteria, trial procedures, or the analytical strategy based on interim results.

In response to this comment, we would clarify this explicitly in the revised manuscript by adding:

"The interim analysis did not affect any other methodological decisions."

Reviewers' Comment:

The use of last observation carried forward (LOCF) in conjunction with MMRM is statistically inconsistent. MMRM already handles missing data under the missing-at-random (MAR) assumption using maximum likelihood estimation. LOCF violates that assumption, biases variance estimates, and can distort treatment effects. The analysis should rely solely on MMRM or use multiple or reference-based imputation methods for sensitivity checks

Reply from authors

Thank you for this important comment.

In our Statistical Analysis Plan, we prespecified two ITT approaches for handling missing data:

"We will conduct two separate ITT analyses:

(1) In the event of missing measurements at different time points, we will compensate by using the last observation carried forward (LOCF) method.

(2) One analysis with no imputed values."

In practice, this led us to combine LOCF with MMRM as the primary analysis and to use MMRM without imputation as a secondary analysis. As we report in the manuscript, both approaches yielded very similar results.

We agree with the reviewer that, under a MAR assumption, MMRM using maximum likelihood is sufficient and that LOCF may be redundant.

In a revised manuscript, we would therefore:

1. Rely on MMRM without LOCF as the primary ITT analysis, and

2. Relegate the MMRM+LOCF analysis to a secondary/sensitivity analysis in the Supplement, or omit it entirely, depending on the Editor's preference.

We would also revise the Methods and Statistical Analysis sections to:

- Remove LOCF as part of the main analytic strategy;

- Clarify that the primary analysis uses MMRM under MAR with maximum likelihood estimation; and

- Explicitly state that sensitivity analyses (with and without any imputation) produced consistent findings, supporting the robustness of the results to the choice of missing-data handling strategy.

As noted in our response to the related comment on ITT, including participants without any follow-up data would not materially change the results: we have already conducted analyses using both LOCF and observed (non-imputed) data, and the conclusions are unchanged across these approaches.

Note to the Editors:

-We would appreciate guidance on how best to address Reviewer #1's comment regarding the handling of missing data. We are fully prepared to present as the main analysis (in line with this recommendation) (i.e., relying on MMRM without LOCF). At the same time, we note that our current approach followed the pre-specified Statistical Analysis Plan, and that the other two reviewers considered the missing-data handling acceptable; one even explicitly wrote:

"Though mixed-effects model does not require imputation of missing values, however proper imputation of missing values are always recommended, which the article did."

Reviewers' Comment:

Twelve preregistered accelerometry outcomes were listed, yet only four are reported. Unless clearly justified (e.g., data loss or redundancy), this constitutes selective outcome reporting. Either all preregistered outcomes should appear in the supplementary material, or the omission should be transparently explained. Without correction for multiple testing, secondary and exploratory results must be explicitly labeled as exploratory to avoid overstating significance.

Reply from authors

We thank the reviewer for this important comment and fully agree that preregistered outcomes should be handled transparently.

For the parameter "distance travelled" specified in the protocol, we relied on functionality available in a different accelerometer model. During implementation, we chose a device that was more practical and reliable for continuous monitoring but did not support all of the advanced features required for this planned variable. Consequently, although core actigraphy-based measures were successfully obtained, "distance travelled" and some other secondary metrics could not be derived as originally intended.

-In the present manuscript, we report light physical activity, awake time, sedentary time, and moderate-to-vigorous physical activity, as these variables are most directly related to our primary clinical question and are the most established variables regarding processing and validation. All these measures are not independent variables but components of the same 24-hour time budget. An increase in one category necessarily corresponds to a decrease in another, making these measures highly collinear and conceptually interdependent rather than separate hypotheses.

-In the revised version, we can also report step count, which is conceptually closely related to these already reported variables. The remaining preregistered accelerometry outcomes are:

1. **Detailed sleep parameters** (e.g., sleep latency, wake after sleep onset, deep sleep, sleep efficiency) and **Distributional measures of movement patterns**

These require substantial additional methodological work specific to this patient population (e.g., developing and validating algorithms and cut-offs in depressive cohorts). Completing and properly validating these analyses would require a few months of additional work.

To address concerns about selective reporting, we will:

- Clearly list all preregistered accelerometry outcomes in the Methods or Supplement,
- Specify which outcomes are reported in this manuscript and justify that choice as mentioned above

-Regarding multiplicity, we will:

1. State explicitly that no formal correction for multiple testing was applied to secondary and exploratory outcomes.

2. Emphasize in the Discussion that they should be interpreted with appropriate caution and viewed as requiring replication, given the increased risk of Type I error in the absence of multiplicity adjustment.

We hope that this transparent handling of the preregistered accelerometry outcomes satisfactorily addresses the reviewer's concerns.

Reviewers' Comment:

- Methodologically, the trial would benefit from additional detail on randomization, allocation concealment, and blinding procedures (per CONSORT 2025). The manuscript does not specify sequence generation, block sizes, or whether raters were involved in dose adjustments, which could compromise blinding given pramipexole's side-effect profile. Adverse-event assessment procedures are also insufficiently described.

Reply from authors

In response to this comment, we would revise the methods section (Intervention) as follows:

"Extended release pramipexole tablets from STADA Nordic were labelled and repackaged consistent with EU guidelines and good manufacturing practice (GMP). Identical placebo tablets were produced by Ardena in line with Good Manufacturing Practice. All tablets were packed in boxes and assigned randomized numbers. Only Ardena and an unblinded research nurse (who was not involved in patient

assessments) had access to the code list connecting the randomization numbers to treatment allocation. Pramipexole tablets were taken once daily, preferable at nighttime. Patients, research physicians and nurses involved in patient assessments were blind for treatment allocation. Since both pramipexole and placebo were packaged by Ardena, eight different logs for the study medication were created, and four different strengths were ordered and combined to achieve the correct dosing. For each strength, both pramipexole and placebo were available. These logs were accessible only to the unblinded staff who packed and labeled all boxes with the participants' randomization numbers. The unblinded staff was not involved in symptom assessments. Blinded study personnel then administrated these to the patients using flexible dosing schedule (Supplementary Table 4). An unblinded statistician at Clinical Studies Sweden Forum South prepared the random allocation sequence and established the randomization list. Apart from the preparation of the allocation sequence, the statistician was not involved in the study. The block size varied randomly between 4 and 6, and the order within the blocks was generated randomly using a computer-based algorithm. Only the statistician who created the randomization list was aware of the random variation in block size and was also the only person who had access to the randomization list. At the week 9 visit, after clinical ratings, patients were asked if they believe they had been allocated to pramipexole or placebo. At the start of the study, participants only had two options: pramipexole or placebo. During the study, several patients described their guess as a "coin-toss". We therefore added the possibility of answering "unsure".

We would also add:

"To maintain blinding in the RCT, a separate team of study physicians and nurses conducted the open-label extension phase. Thus, none of the physicians responsible for patient assessments in the RCT participated in the open-label phase, ensuring they remained unaware of any side effects or efficacy signals that could compromise blinding during the randomized phase."

We would also add a new section in Methods, safety assessments:

"At each visit, including telephone follow-ups, participants were systematically asked about AEs. All reported AEs, including known side effects of pramipexole, were documented in an individual patient log. Each AE was evaluated by the study physician with regard to (a) severity, (b) likelihood of relatedness to pramipexole, and (c) whether it met criteria for a serious adverse event. The study physicians regularly discussed AE assessments to promote consistent and harmonised evaluation across patients."

Protocol changes are summarized in a table at the end of the Study Protocol (attached in the original submission)

Reviewers' Comment:

Appropriate use of statistics and treatment of uncertainties
The statistical framework is partially sound but inconsistently applied.
- For the secondary outcomes, reporting both non-significant endpoint differences and significant "main effects across time" without clarifying which estimand was primary risks post-hoc reinterpretation.

Reply from authors

We thank the reviewer for highlighting this point. We agree that the distinction between the endpoint estimand and the longitudinal "main effect across time" needs to be made more explicit.

In our Statistical Analysis Plan, the **primary outcome** was defined as the between group difference in absolute change from baseline to week 9 on the SHAPS (i.e. an endpoint estimand). However, the **sample size calculation** was based on detecting a treatment main effect in a mixed effects model, which is closely related but not identical to the endpoint contrast.

In the manuscript, we therefore reported both (i) the between group difference in change at the week 9 endpoint, consistent with the prespecified primary outcome, and (ii) the overall treatment main effect from the MMRM, as a complementary longitudinal estimand. We acknowledge that this dual reporting may appear inconsistent if the hierarchy between these estimands is not clearly stated.

In a revised version, we would:

- Clearly state in the Methods that the **primary estimand** is the between group difference in change from baseline to week 9, as prespecified in the SAP;
- Explicitly describe the MMRM treatment main effect as a **secondary, supportive estimand** that leverages all available longitudinal data; and
- Harmonize the Results and Discussion to consistently treat the endpoint contrast as primary, and the longitudinal main effect as supplementary, avoiding any post hoc reinterpretation.

We hope that this clarification and reframing address the reviewer's concern about the consistency of the statistical framework.

Reviewers' Comment:

- Multiplicity is another concern. Multiple endpoints (SHAPS, MADRS-S, HDRS-6, GAD-7, ISI, AES-S, BBQ, 12 accelerometry variables, fMRI outcomes) and multiple visits (weeks 3, 6, 9) create numerous testing opportunities. The manuscript does not describe any adjustment for family-wise error or false-discovery rate. Without such correction, reported p-values must be considered nominal.

Reply from authors

We thank the reviewer for raising the issue of multiplicity. We agree that the number of endpoints creates multiple testing opportunities.

In our Statistical Analysis Plan, we did not pre-specify any formal adjustment for family wise error rate or false discovery rate beyond designating the primary clinical endpoint. The only outcome defined as primary was the between group difference in change from baseline to week 9 on the SHAPS. All other clinical scales, accelerometry variables, and fMRI outcomes were prespecified as secondary or exploratory.

In a revised manuscript, we will therefore:

- Explicitly state in the Methods which endpoint is the **single primary outcome**, and
- Clearly label all other endpoints (clinical, accelerometry, fMRI) as **secondary or exploratory**,
- Describe all associated p-values as **nominal**, and emphasise that they should be interpreted with appropriate caution in light of multiplicity.

We hope that this clarification and reframing adequately addresses the concern about multiplicity and prevents overinterpretation of secondary and exploratory findings.

Reviewers' Comment:

Conclusions: robustness, validity, reliability
The finding that pramipexole improves anhedonia is supported by the primary SHAPS analysis but should be interpreted with caution. The lack of consistent effects on standard depression scales weaken claims of overall antidepressant efficacy. Assertions that pramipexole may be suitable for less-resistant depression are speculative without corresponding subgroup or sensitivity analyses. Long-term efficacy and tolerability remain untested beyond the nine-week period, limiting validity. Nevertheless, the consistent mechanistic evidence from fMRI and activity data provides convergent support for dopaminergic modulation of motivation, enhancing the biological plausibility of the findings.

Reply from authors

We thank the reviewer for this thoughtful summary and agree that the conclusions should reflect both the strengths and the limitations of the current data.

Regarding **overall antidepressant efficacy**, we concur that the most robust signal is for anhedonia (SHAPS), while effects on broader depression scales are less consistent. In the revised manuscript, we will temper statements about global antidepressant efficacy and more clearly state that the strongest evidence pertains to anhedonic symptoms, with broader mood effects requiring further confirmation.

With respect to **less treatment resistant depression**, we accept that our current data do not justify strong claims about suitability in milder or less resistant populations. We will therefore remove or substantially soften such statements and explicitly label any discussion in this direction as speculative and hypothesis-generating rather than evidential.

Finally, regarding **long term efficacy and tolerability**, we agree that the nine-week RCT limits conclusions about durability. To address this, we will incorporate data from our recently completed 6-month open label extension in the same cohort, summarising longer term symptom trajectories and adverse events.

We hope these revisions address the reviewer's concerns about robustness, validity, and reliability of the conclusions.

Reviewers' Comment:

Suggested improvements

1. Re-run all primary and secondary analyses in a full ITT dataset including all randomized participants.
2. Eliminate LOCF imputation; rely on MMRM or modern multiple imputation techniques.
3. Provide complete reporting of preregistered accelerometry endpoints (e.g., as supplementary data) and clarify how multiplicity was addressed.
4. Explicitly define the statistical hierarchy: which contrast (week 9 or averaged effect) is primary, which outcomes are secondary, and how multiplicity was controlled.
5. Add full CONSORT 2025-required details: randomization method, blinding maintenance, AE definitions, and any protocol changes.
6. Discuss the potential bias introduced by the interim analysis and describe its influence on subsequent study conduct.
7. Temper the interpretation of efficacy in less-resistant or dysthymic patients unless additional analyses support this claim.
8. Add a clearer discussion of the lack of significant effects on MADRS-S and HDRS-6 and the implications for overall clinical benefit, especially in contrast to Browning et

al. (Lancet Psychiatry, 2025), who demonstrated antidepressant efficacy in treatment-resistant depression.

Reply from authors

We thank the reviewer for these constructive comments and believe we have addressed each of them in our responses above.

References
Prior work is cited appropriately.

Reviewers' Comment:
Clarity and context
The manuscript is well written and visually appealing, but some elements of transparency are missing.
- The flow diagram should follow CONSORT standards, listing all randomized participants, discontinuations, and analysis sets.

Reply from authors

We would provide this in a revised version of the paper

Reviewers' Comment:

- Statistical group comparisons at baseline are inappropriate in randomized trials, as any differences arise by chance. Baseline data should be reported descriptively only.

Reply from authors:

This would be done in a revised version of the paper

Reviewers' Comment:

- Present absolute values alongside percentages in the baseline table and include clinically relevant variables (duration of current episode, concomitant psychotherapy).

Reply from authors:

This would be done in a revised version of the paper, although we do not have information about duration of the current episode. During the study, one participant in the placebo arm and two in the pramipexole arm reported receiving concomitant psychotherapy. These were ongoing, stable treatments, as an exclusion criterion was recent initiation of psychotherapy (within 6 weeks) or plans to start psychotherapy during trial participation.

Reviewers' Comment:

- The abstract and conclusions should more accurately reflect that the primary improvement was limited to anhedonia, with broader depressive outcomes not reaching statistical significance.

Reply from authors:

The abstract would be revised accordingly in the next version of the manuscript.

Reviewers' Comment:

Overall, this is a well-executed and conceptually important trial with strong mechanistic evidence. Addressing the statistical and reporting issues noted above would substantially strengthen its credibility and impact.

Congratulations to the authors for this ambitious and innovative study, and best wishes from Munich,
[redacted]

Reply from authors:

We are very grateful for the reviewer's thoughtful and encouraging evaluation of our work.

Reviewer #3 (Remarks to the Author):

A. Summary of Key Results
Participants treated with pramipexole exhibited a significantly greater reduction in anhedonic symptoms compared with those receiving placebo. Consistent benefits were also observed across secondary measures assessing anhedonia and apathy. Objective accelerometry data corroborated these effects, showing increased physical activity among individuals in the pramipexole group. Functional imaging further demonstrated enhanced reward-related activation in the ventral striatum, which was associated with reductions in anhedonic symptom severity. Overall, pramipexole was well tolerated, with low attrition and no serious adverse events reported.

B. Originality
The study builds on previous clinical and observational evidence by offering new mechanistic insights into pramipexole's antidepressant effects. It demonstrates that pramipexole engages the brain's reward system while also being the first antidepressant RCT to include objective measures of physical activity, showing that the drug alleviates anhedonia both subjectively and through measurable increases in daily activity.
C. Data & methodology: validity of approach, quality of data, quality of presentation
Mixed-model analyses demonstrated that pramipexole led to significantly greater improvement in anhedonic symptoms compared with placebo, with consistent effects across all time points and robustness in completer analyses. Secondary outcomes showed notable reductions in anhedonia and apathy, particularly in motivational domains, with a modest overall benefit on depressive symptoms but no significant effects on anxiety, insomnia, or quality of life. Accelerometry data indicated increased light physical activity in the pramipexole group, suggesting enhanced behavioral engagement independent of self-reported anhedonia. Neuroimaging results revealed preserved ventral striatal activation during reward anticipation in the pramipexole group, consistent with dopaminergic modulation of reward processing. The treatment was well tolerated, with no serious adverse events; side effects such as sleep disturbance, nausea, and dizziness were more common but manageable, and no participants discontinued due to impulse-control symptoms.
D. Appropriate use of statistics and treatment of uncertainties
Mixed-effects models for longitudinal designs and paired t-test for pre-post comparison are often used in the literature. Though mixed-effects model does not require imputation of missing values, however proper imputation of missing values are always recommended, which the article did.
E. Conclusions: robustness, validity, reliability
This article demonstrated with small samples that pramipexole is an effective and well-tolerated augmentation treatment for anhedonia in mood disorders, offering both clinical and mechanistic evidence of efficacy. By targeting patients with pronounced anhedonia, the trial confirmed that pramipexole alleviates motivational deficits, enhances physical activity, and preserves ventral striatal activation associated with reward processing. The study extends prior findings by incorporating objective measures such as accelerometry and fMRI, strengthening the evidence for dopaminergic modulation as a mechanism of action. Pramipexole showed benefits across depressive subtypes—including MDD, dysthymia, and bipolar depression—supporting its use as a transdiagnostic, symptom-focused treatment. The drug was generally well tolerated, with mostly mild and manageable side effects and a low

dropout rate. Together, these results highlight pramipexole as a safe, efficacious, and mechanistically targeted therapy for anhedonic depression, and illustrate the value of multimodal, precision-based approaches in antidepressant research. However, the study sample size was small, hence in order to get a definite usable result more studies with large samples are required.

Reviewers' Comment:

F. Suggested improvements
The main finding of the study is that, at the population level, patients randomized to the pramipexole intervention showed greater improvement than those receiving placebo. However, several important questions remain unanswered: Which patients within the intervention group derived the most benefit—was it a general effect or limited to those with specific characteristics? What proportion of patients experienced meaningful symptom improvement? And, critically, can we identify the subgroup of individuals most likely to respond favorably to pramipexole?

Reply from authors:

We agree that these are central questions and are closely aligned with the broader aims of "precision psychiatry." The trial was partly designed with this objective in mind by enriching the sample for patients with more severe anhedonia, a subgroup we hypothesised *a priori* might be particularly responsive to pramipexole.

The fMRI component was likewise partly intended to address heterogeneity of treatment response. In our analyses, we observed a trend towards an association between greater clinical improvement and increased reward-related activation in the ventral striatum, which is consistent with the possibility of a biologically defined subgroup showing enhanced benefit from pramipexole.

In a revised manuscript, we would make this rationale more explicit and we are also willing to add exploratory analyses of baseline clinical and demographic treatment predictors and to describe the proportion of patients achieving predefined thresholds of meaningful symptom improvement, while clearly labeling these analyses as exploratory.

Using the extended follow-up phase, we will also be able to examine response and remission rates over time, recognizing that a 9-week window is typically too short to draw firm conclusions about remission.

Reviewers' Comment:

The neuroimaging component of the study is at the rudimentary level, does not give a deep understanding of the mediation or causal mediation effects of the networks.

Reply from authors:

We thank the reviewer for this comment and agree that a more extensive neuroimaging analysis (e.g. formal mediation or causal mediation modelling of network effects) would be of interest.

However, the fMRI analyses reported in the manuscript were prespecified in the study protocol and Statistical Analysis Plan as our primary target-engagement measures. Our selection of regions of interest was based on prior related work by Krystal et al. Given the sample size and the number of other clinical and mechanistic endpoints, we deliberately limited the neuroimaging component to these predefined contrasts to (i) maintain alignment with the preregistered aims, and (ii) avoid inflating the risk of false-positive findings through extensive post-hoc exploration, particularly in light of the broader multiplicity concerns raised by another reviewer.

In the revised manuscript, we would:

- State more explicitly that the neuroimaging analyses were limited to preregistered target-engagement contrasts;

- Given that we will now include longer-term efficacy data (up to 6 months beyond the 9-week primary endpoint), we will be able to examine whether baseline fMRI characteristics in our regions of interest, as well as treatment-related changes in reward-processing activation, are associated with longer-term response and remission to pramipexole.

We agree that this still falls short of a full causal-mediation framework, and the study was not powered or designed for formal mediation or network-level causal modeling. However, adding these longitudinal imaging–outcome associations will strengthen the neuroimaging component and provide more informative, hypothesis-generating data on putative mechanistic pathways that can be tested with more sophisticated mediation designs in future work. In the Discussion we would acknowledge that we did not perform formal mediation or causal mediation analyses and that this remains an important avenue for future work.

We hope this clarification explains the rationale for the current level of neuroimaging analysis while recognising the value of the deeper approaches the reviewer suggests.

G. References: appropriate credit to previous work
References are appropriately cited.
H. Clarity and context
The article is well written.

Reviewer #4 (Remarks to the Author):

This article reports on a robust, methodologically sophisticated, and clinically significant randomized controlled trial (RCT). It provides compelling evidence that pramipexole is an effective and well-tolerated augmentation therapy for anhedonia across mood disorders. The study's major strength lies in its multi-modal assessment strategy, which moves beyond subjective reports to include objective behavioral (accelerometry) and neurobiological (fMRI) measures of target engagement.
Technical Strengths
1. The study is based on a precise and mechanistically-grounded hypothesis. It goes beyond a generic "depression" diagnosis to target a specific symptom cluster (anhedonia) with a drug (a dopamine D2/D3 receptor agonist) that directly engages the hypothesized underlying neurobiology (mesolimbic dopamine pathway dysfunction). This aligns well with the RDoC framework and may prospectively support precision psychiatry.
2. The entire study is based on a rigorous transdiagnostic design and shows that the treatment effect is linked to the symptom (anhedonia) rather than the diagnostic label. This may increase the generalizability and clinical relevance of the findings.
3. The authors provide a multi-modal outcome assessment, which may be the study's most significant strength. The primary outcome (SHAPS) was specific to anhedonia, and secondary scales (DARS, AES-S) provided a nuanced view of motivational and consummatory pleasure. The use of accelerometry is certainly an innovation in antidepressant trials. It provides an objective, ecologically valid measure of real-world behavior change (increased light physical activity), which is a direct behavioral correlate of improved motivation and energy. The finding that this objective measure aligned with subjective reports strongly supports the clinical efficacy of pramipexole. The use of neuroimaging data (7-Tesla fMRI) with the Monetary Incentive Delay (MID) task is also an interesting twist. The high magnetic field strength provides high signal-to-noise ratio for imaging small subcortical structures like the ventral striatum. Demonstrating a significant between-group difference in reward-related activation provides direct evidence of target engagement in the brain's reward circuitry.
4. The core statistical methodology is based on Mixed-Model Repeated Measures (MMRM), a gold standard for analyzing longitudinal clinical trial data, and uses all available data points and handles missing data appropriately. The Hedge's g effect sizes allow for a clear interpretation of the magnitude of the effects and the sensitivity analyses with/without Last Observation Carried Forward (LOCF).
A remarkably low dropout rate of 6% in a 9-week trial, with no serious adverse events attests to the feasibility and acceptability of this treatment regimen in clinical practice; the slow titration schedule likely contributed to this.

Reviewer Comment:

Potential Study Limitations

1. Partial Unblinding in the Placebo Group: This is the most significant methodological weakness. The fact that 66.7% of the placebo group correctly guessed their allocation (p=0.053) introduces a potential for bias, particularly for subjective self-report measures like SHAPS, DARS, and AES-S. Participants knowing they are on placebo may experience disappointment, amplifying their perception of no improvement (a nocebo effect). However, this weakness is substantially mitigated by the positive findings on the objective accelerometry data, which is less susceptible to expectation bias.

Reply from authors:

Thank you for raising this important point. We agree that assessing and reporting blinding integrity is essential in psychiatric RCTs.

-Few psychiatric RCTs formally evaluate blinding integrity, despite growing consensus that they should. In our trial, we prospectively assessed and transparently reported blinding. Although blinding eroded in the placebo arm, guess accuracy in the pramipexole arm remained near chance and could not plausibly account for the primary efficacy results, including at higher doses.

-To help interpret correct guesses, we asked participants to provide reasons. Among placebo recipients who guessed correctly (n=26), 46% (n=12) cited lack of improvement, 31% (n=8) cited lack of improvement plus absence or minimal side effects, 4% (n=1) cited other reasons, and for 19% (n=5) we did not ask this question. Among pramipexole recipients who guessed correctly (n=18), 33% (n=6) cited improvement, 28% (n=5) cited improvement plus side effects, 11% (n=2) cited side effects alone, and for 28% (n=5) we did not ask the question.

-Overall, these patterns suggest that guess accuracy primarily tracked perceived clinical response—and, to a lesser extent, tolerability—rather than systematic unblinding. This highlights a common challenge in psychiatric trials that rely on subjective outcomes: effective treatments can produce apparent or “false” unblinding, where improvement inform guesses without compromising allocation concealment.

In the revised manuscript we will report the distribution of guesses and reasons as listed above (Results, blinding integrity). We have also revised the discussion (page 23) related to blinding integrity as follows:

“Few psychiatric RCTs evaluate blinding integrity, despite growing consensus that they should. In our trial, we prospectively assessed and transparently reported blinding. About two-thirds of the patients in the placebo group correctly guessed their assignment, whereas guess accuracy in the pramipexole group was near chance. This asymmetry is a study limitation, as it could introduce expectancy effects and bias subjective symptom reports. Participants most often attributed correct guesses to clinical improvement (pramipexole) or lack of improvement (placebo), sometimes

combined with the presence (pramipexole) or absence (placebo) of side effects. These patterns highlight a broader challenge in psychiatric RCTs that rely on subjective outcomes: guess accuracy tends to track perceived response and, to a lesser extent, tolerability, rather than systematic unblinding. To address this, we incorporated more objective outcomes and target-engagement measures, including accelerometry and fMRI, which help mitigate bias and strengthen the robustness of our findings."

Reviewer Comment:

The effect sizes are modest. The primary outcome effect size (Hedge's g=0.63) is marginal, which is not atypical for psychopharmacology trials but may suggest the effect is not overwhelming.

Reply from authors:

We appreciate the reviewer's comment and agree that the primary effect size is in the moderate range. At the same time, we note that this magnitude is larger than many effect sizes typically observed in antidepressant RCTs, particularly in treatment-resistant or otherwise enriched samples, and that it was obtained in a rigorously controlled, transdiagnostic trial.

Reviewer Comment:

The effect on the fMRI outcome was statistically significant but small (g=0.32) and the correlation between clinical improvement and neural change within the pramipexole group shows only a trend (p=0.06). This indicates that while pramipexole engages the ventral striatum on a group level, the link between this engagement and individual symptom improvement is less robust and requires further investigation in larger samples.

Author response

We thank the reviewer for this thoughtful comment and fully agree that the relationship between neural target engagement and individual clinical response is an important question that warrants further investigation in larger samples.

However, we would like to clarify a misunderstanding regarding the effect size. The Hedges' g for the main fMRI contrast is 0.64, not 0.32. The value 0.32 in the manuscript refers to the estimated sigma parameter, not the standardized between-group effect size. This discrepancy reflects a typo error on our part, for which we apologize. Thus, the magnitude of the group-level ventral striatal

engagement is in the moderate range and aligned with the primary clinical effect size.

That said, the reviewer's broader point still holds: although we observe statistically significant target engagement at the group level, the within-pramipexole correlation between neural change and symptom improvement is only at trend level ($p = 0.06$) and should be interpreted cautiously.

In the revised manuscript, we would:

- Correct any wording that might have contributed to the confusion about the effect size and explicitly state that the main fMRI effect is $g = 0.64$.
- Acknowledge in the Discussion that, despite moderate group-level target engagement, the association between neural change and individual clinical benefit is not robust in this sample and remains a key question for future, larger studies.

We hope this clarification and added a more nuanced language to address the reviewer's concern.

Reviewer Comment:

While the overall intention-to-treat sample (n=82) is reasonable, the sample sizes for the accelerometry (n=61) and especially the fMRI (n=48) sub-studies are smaller. This reduces the power of these analyses and increases the risk of Type II errors (missing real effects), see the statistical-power comment at the end.

Author response

We thank the reviewer for highlighting the issue of sample size and power for the accelerometry and fMRI sub-studies.

The original power calculation was based on the primary clinical outcome (SHAPS) and did not explicitly target the accelerometry or fMRI endpoints. As a result, the accelerometry (n = 61) and especially the fMRI (n = 48) analyses are indeed less well powered, and we agree that this increases the risk of Type II errors.

In the revised manuscript, we would:

- State explicitly in the Methods that the trial was powered for the primary SHAPS outcome, not for accelerometry or fMRI effects;

- Emphasise in the Results/Discussion that the accelerometry and fMRI findings should be viewed as secondary, hypothesis-generating analyses, interpreted with appropriate caution in light of the smaller sample sizes and reduced power; and

- Note that the risk of missing true effects (Type II error) is correspondingly higher for these mechanistic outcomes, underscoring the need for replication in larger samples.

We hope this clarification addresses the reviewer's concern about power and the interpretation of the sub-study results.

Reviewer Comment:

Patients remained on their existing antidepressants/mood stabilizers, which is rational for an augmentation study, but introduces (drug-interaction) variability. The authors did acknowledge a significant baseline imbalance in SNRI use, as a potential confounder.

Author response

We agree that allowing patients to remain on their existing antidepressants and mood stabilizers introduces pharmacological heterogeneity, including possible drug–drug interactions. This was a deliberate design choice, intended both to reflect real-world augmentation practice and to ensure clinical safety, but we recognize that it increases variability in treatment response.

As the reviewer notes, there was a baseline imbalance in SNRI use, which we have already identified as a potential confounder. In a revised manuscript, we will:

- More explicitly describe this rationale and its implications in the Methods and Discussion sections; and

- Emphasize that concomitant medications, including the imbalance in SNRI use, may have contributed to variability in outcomes.

To our knowledge, however, there are currently no data indicating that SSRIs or SNRIs systematically alter pramipexole exposure or pharmacokinetics, but we agree that the potential influence of background treatments on clinical response should be acknowledged more clearly as a limitation.

Reviewer Comment:

The 9-week duration establishes efficacy but tells less about the potential long-term durability of the response, the need for continued treatment, or the potential for tolerance or long-term side effects.

Author response

We agree with the reviewer that a 9-week double-blind phase provides only limited information about long-term durability, the need for maintenance treatment, and the potential for tolerance or longer-term adverse effects.

To address this, we will incorporate data from our 6-month open-label extension, conducted in the same RCT cohort, into the revised manuscript. In this extension, participants continued pramipexole under open-label conditions, allowing us to examine:

- The durability of clinical response over 6 months,

- Ongoing tolerability and the emergence of any new or cumulative side effects, and

- Patterns of discontinuation.

Reviewer Comment:

I believe the study demonstrates that pramipexole improves motivational anhedonia, but its effect on consummatory anhedonia is less clear.

Author response

In response to this comment, we would respectfully clarify that our primary outcome, the SHAPS, is generally considered a measure of consummatory anhedonia. By contrast, our secondary outcome, the DARS, captures both consummatory and motivational/anticipatory aspects of anhedonia. Taken together, we therefore interpret the pattern of results as indicating that pramipexole exerts effects on both components of anhedonia.

In the revised manuscript, we would:

- Make this conceptual distinction more explicit in the Introduction and Discussion (with supporting citations),
- Clarify which dimensions of anhedonia each scale is intended to capture, and
- Rephrase our conclusions to state that pramipexole appears to improve both consummatory and motivational aspects of anhedonia.

Reviewer Comment:

> A specific technical comment: Please double check, and explicate in the appendix, the exact details of the study power calculations. I used Lu, Luo, & Chen (2008) methodology (implemented in R's longpower package), to verify the MMRM power analysis for this study's longitudinal designs. I am getting somewhat different results, a sample-size estimate of 256 (far exceeding the reported sample-size of n=80). If IBM SPSS was used for power calculations, perhaps try to cross-check with R.

Author response

We thank the reviewer for this careful technical comment and for independently checking the power calculations.

Our original sample size calculation was performed for the MMRM treatment main effect under a specific set of assumptions (effect size, correlation structure, number and spacing of timepoints, dropout pattern, and variance components). In the SAP, however, we defined the primary outcome as the between-group difference in change from baseline to week 9 on the SHAPS, i.e. an endpoint contrast. Thus, there is a slight mismatch between the estimand used for the power calculation (longitudinal main effect) and the way the primary outcome was framed in the SAP, even though the two are closely related.

In a revised manuscript, we will:

- Provide a more detailed description of our power calculation in an Appendix, including:
    - the assumed standardized effect size,
    - the number and timing of assessments,
    - the assumed within-subject correlation structure,
    - the expected dropout rate, and
    - the software/procedure used.
- Explicitly acknowledge that our calculation targeted the MMRM main effect rather than an endpoint contrast, and that alternative methods (such as Lu, Luo & Chen) under different assumptions can yield higher required sample-size estimates.
- Emphasize that, in light of these issues, our trial should be regarded as moderately powered for the primary clinical effect and underpowered for some secondary/mechanistic outcomes, reinforcing the need to interpret some of those results with caution.

We are also willing to replicate our original calculation using R/longpower under our assumed parameters and report both sets of estimates in the Appendix to enhance transparency.

Editorial queries

Editorial points to be addressed. Please note that not all of these might apply to your manuscript, in which case please respond with “not applicable.” Structural changes to re-organise the manuscript do not need to be tracked.

Author response

We have addressed all editorial points below. Structural changes involving reorganisation of the manuscript have not been tracked, in accordance with the instructions. All substantive revisions to the text have been made using track changes.

* Abstract should be no more than 200 words and for trials should adhere to the CONSORT framework. You must state if the primary outcome was or was not met and provide the effect size and relevant uncertainty estimate. The conclusion of the study must focus only on the primary outcome and safety/tolerability. You must report either all or none of the secondary outcomes, given the space limitation, I would suggest removing all mention of secondary outcomes from the abstract.

Author response

We have rewritten the abstract accordingly. At the same time, we have retained a single brief sentence describing the fMRI and accelerometry findings, clearly labelled as exploratory, as we consider these to reflect key mechanistic aspects of the study and an important part to its novelty. We believe this addition provides helpful context for readers who may only access the abstract, while remaining concise and aligned with the overall guidance. That said, if you feel strongly that this sentence should be omitted, we would of course remove it entirely.

* Please include the trial registration number at the end of the Abstract.

Author response

The requested edit has been done.

* The Introduction should be written for a broad, non-specialist medical reader and provide sufficient context for the work.

Author response

We have revised the intro in accordance with these comments

* Please provide details of your cohort in the first paragraph of the Results. This includes number of individuals screened for enrolment, as well as exact dates of first and last patient enrolment. The CONSORT patient disposition diagram and the baseline characteristics must be included as main non-text items (for which there is a strict limit of 6 tables and/or figures).

Author response

This has been added

* The Results should be structured as followed:
- Patient disposition
- Primary outcome(s)
- Secondary outcomes
- Safety
- Exploratory outcomes
- Sensitivity analyses
- Post-hoc analyses

Author response

We have structured the Results section according to these instructions and incorporated the section “planned statistical analysis” (previously in results) in the methods section. FMRI and accelerometry outcomes are listed under “exploratory outcomes”.

* Please remove any subheadings from the Discussion.

Author response

The requested edit has been done.

* Please ensure all results are presented in the Results section, no new data should be introduced in the Discussion.

Author response

Confirming that no new data were introduced in the Discussion.

* You must include explicit paragraphs of study limitations in the Discussion.

Author response

We have now consolidated the discussion of study limitations into a dedicated paragraph in the Discussion section, beginning with “This study has several limitations that should be considered when interpreting the findings.”

* The overarching conclusion of the study must be based only on the primary outcome and safety data.

Author response

We have revised the conclusion accordingly (both in abstract and in main text)

* The Methods should include a full description of the inclusion and exclusion criteria, as well as study procedures and statistical analyses (including a power calculation).

Author response
We have revised the Methods section accordingly, including key inclusion/exclusion criteria plus a supplementary table with all eligibility criteria. We have also edited the methods section to start with the trial design before patient population and included statistical analyses.

*Please upload the protocol and SAP with the revision materials, so that reviewers and editors have access to them.

Author response

As requested, these documents have been uploaded

* You must ensure that contributions from all individuals in the author list are available in the Author Contributions statement.

Author response

The requested statement has been added.

* Any relevant funding should be declared in a separate funding statement. Please refer to our funding statement guidelines (https://www.nature.com/nature-portfolio/editorial-policies/funding) for more information.

Author response

The requested statement is included.

* Please ensure that all potential competing interests are detailed for all authors. For any authors with no competing interests, this must also be stated.

Author response

Confirming that the requested statement is included.

* Please see our guidelines for the Data and Code Availability Statements. “Available on request” is not acceptable, you must provide details of any restrictions to data and code availability. https://www.nature.com/nature-portfolio/editorial-policies/reporting-standards#availability-of-data

Author response

We have edited the data availability statement to meet Nature Medicine guidelines

* The article file must only contain these items in this order:
• Title
• Author List and affiliations
• Abstract
• Introduction
• Results (with Subheadings)
• Discussion
• Acknowledgements
• Author Contributions
• Competing Interests Statement
• References (for main text only)
• Figure legends (for main text only)
• Tables (note: tables should be pasted into Word files as editable tables, not as images)
• Methods
• Data Availability Statement
• Code Availability Statement
• Methods-only References

Author response

We have restructured the manuscript according to these guidelines.

* Submit both clean and tracked changes versions of the revision as editable Word documents.

Author response

Structural changes involving reorganisation of the manuscript have not been tracked, in accordance with the instructions. All substantive revisions to the text have been made using track changes.

* Include a revised version of any required reporting checklist. The reporting summary can be found at https://www.nature.com/documents/nr-reporting-summary.pdf. Please note that this form is a dynamic 'smart pdf' that must be downloaded and completed in Adobe Reader (i.e. cannot be opened in a web browser).

Author response

We have attached a reporting checklist

Nature journals have recently announced an update to our guidance on reporting on sex and gender in research studies (see here). We strongly encourage researchers to follow the 'Sex and Gender Equity in Research – SAGER – guidelines' and to include sex and gender considerations for studies involving humans, vertebrate animals and cell lines where relevant to the topic of study (an overview can be found here). Authors should use the terms sex (biological attribute) and gender (shaped by social and cultural circumstances) carefully in order to avoid confusing both terms.

When preparing your revised manuscript, please be aware of our guidance on Sex and Gender reporting). Please note that:

1. If the research findings apply to only one sex or gender, that must be indicated in the title and/or abstract.

Author response

N/A

2a. For studies involving vertebrates animal and cell lines- The Reporting Summary should include whether sex was were considered in the study design.

Author response

N/A

2b. For studies involving human research participants- The Reporting Summary should include whether sex and/or gender was considered in the study design and whether sex and/or gender of participants was determined based on self-report or assigned (and methodology used).

Author response

This information has been included.

3. Data should be reported disaggregated for sex and gender where this information has been collected and consent has been obtained for reporting and sharing individual-level data; disaggregated numbers for individual experiments must be provided in the source data as appropriate whereas overall numbers may be provided in the Nature Portfolio Reporting Summary.

Author response

Analyses were conducted in the overall sample. Participant sex is included in the source data, allowing disaggregated analyses.

Information on the 3 points above should be included in the revised manuscript and detailed in the cover letter.

Author response

This has been added in the reporting summary

In addition, please note that if sex- and gender-based analyses have been performed a priori, results should be reported regardless of positive or negative outcome. We discourage conducting post hoc sex- and gender-based analysis if the study design is insufficient (for example, low sample size) to enable meaningful conclusions.

If no sex- and gender-based analyses have been performed, please indicate the reasons for the lack of these analyses in the Reporting Summary.

Author response

In the Reporting Summary we have now added (p. 2): "Sex was recorded for all participants and is reported descriptively. The study was not designed or powered to detect sex-specific effects, and therefore no formal sex-stratified analyses were performed. Participant sex is included in the source data, enabling disaggregated analyses."

When submitting the revised version of your manuscript, please also pay close attention to our href="https://www.nature.com/nature-research/editorial-policies/image-integrity">Digital Image Integrity Guidelines. and to the following points below:

-- that unprocessed scans are clearly labelled and match the gels and western blots presented in figures.
-- that control panels for gels and western blots are appropriately described as loading on sample processing controls
-- all images in the paper are checked for duplication of panels and for splicing of gel lanes.

Author response

N/A

Finally, please ensure that you retain unprocessed data and metadata files after publication, ideally archiving data in perpetuity, as these may be requested during the peer review and production process or after publication if any issues arise.

Author response

Ok

Reviewers' Comments:

Reviewer #1 (Remarks to the Author):

The authors have substantially improved the reporting of methods and results and now offer a more balanced discussion of their primary and secondary outcome findings. I welcome the additional inclusion of follow-up data, even if open-label and thus preliminary in nature. The authors have adequately addressed my previous concerns, and I recommend acceptance for publication.

Author response

We sincerely thank the reviewer for their constructive feedback and are grateful for his positive assessment of the revised manuscript.

Reviewer #3 (Remarks to the Author):

A. Summary of the key results
   The primary analysis is conducted using mixed-model repeated measures (MMRM) in the modified intention-to-treat sample with baseline adjustment, which is an appropriate framework for longitudinal clinical trial data. The reported treatment effect on SHAPS scores at week 9 (mean difference −4.04; 95% CI −6.89 to −1.18; p=0.006; Hedges' g ≈ 0.62) indicates a statistically detectable improvement associated with pramipexole during the randomized phase. Sensitivity analysis using last-observation-carried-forward produced similar estimates. Baseline SHAPS values were broadly comparable between treatment groups, and estimated marginal mean comparisons favored pramipexole at the evaluated timepoints.
   B. Originality and significance: if not novel, please include reference

Remarks from authors

In several instances, the Reviewer raised similar or overlapping points across different sections of their review. We have therefore consolidated these comments and addressed them together.

Comment from reviewer: The trial involved a relatively small sample (about 85 participants), which may limit statistical power and precision of effect estimates.

Author response

We thank the reviewer for this important comment. The sample size was determined based on an a priori power calculation and was sufficient to detect clinically meaningful effects on the primary outcome, which was met.

We agree, however, that the overall sample size may limit the precision of effect estimates and generalizability. This consideration is particularly relevant for the mechanistic analyses (fMRI and accelerometry), which were conducted in smaller subsamples due to the optional nature of these assessments.

In the revised manuscript, we have addressed these important points in several ways. First, we now explicitly label the mechanistic analyses as exploratory (including a dedicated subheading in the Results section). Second, in the Discussion we clearly acknowledge these limitations. Specifically, we note that the lack of adjustment for multiplicity across secondary outcomes increases the risk of Type I error, while the relatively small subsamples reduce statistical power and increase the risk of Type II error. We explicitly state that these factors may lead to both false-positive and false-negative findings and emphasize the need for replication of the mechanistic results in larger, adequately powered studies.

Comment from reviewer: “The selected population with severe anhedonia may restrict generalizability to broader depression populations”

Author response

We agree that the focus on patients with prominent anhedonia may limit the generalizability of the findings to broader, unselected depression populations.

At the same time, this was a deliberate design choice. The study was based on a predictive enrichment strategy, selecting participants with a symptom profile closely aligned with the proposed mechanism of action (dopaminergic modulation of reward processing). Given the substantial clinical and biological heterogeneity of depression, as well as prominent recent failures in drug R&R when continuing to focus on “MDD all-comers”, such approaches have been increasingly advocated to improve signal detection and enable more mechanism-based treatment development.

In the revised manuscript, we now emphasize that the findings should be interpreted as evidence of efficacy within a clinically and mechanistically defined subgroup, rather than across all depression populations. The following has been added to the Limitations paragraph (p. 14):

“The use of a predictive enrichment strategy, enrolling only patients with prominent anhedonia, may limit generalizability to broader depression populations. However, this approach was deliberately chosen to align the study sample with pramipexole’s proposed mechanism of action and to reduce clinical heterogeneity. Accordingly, the findings should be interpreted as primarily applicable to patients with clinically significant anhedonia.”

Comment from reviewer: “Mechanistic conclusions remain indirect because outcomes are primarily symptom based”.

Author response

We thank the reviewer for this important comment. We agree that symptom-based outcomes alone provide only indirect evidence regarding underlying mechanisms. However, the study was specifically designed to complement clinical outcomes with both objective and neurobiological measures.

In addition to symptom ratings, we included functional MRI to assess reward-related ventral striatal activity and accelerometry to capture objective behavioral changes. These measures were incorporated to provide convergent evidence relevant to the proposed dopaminergic mechanism.

We have clarified this point in the revised manuscript, including a more cautious framing of the mechanistic interpretation. Specifically, we now emphasize that the findings provide supportive—rather than definitive—evidence of target engagement, based on the convergence of clinical, behavioral, and neurobiological outcomes (Discussion, limitations, p. 14):

“While the inclusion of these exploratory outcome measures strengthens mechanistic interpretation, these findings should be considered supportive rather than definitive evidence of target engagement”

Comments from reviewer: “the open label extension lacks a control group” and “improvement observed during the open-label extension phase was reported descriptively and without a concurrent control group, which limits causal interpretation of changes during this period.”

Author response

We agree that the open-label extension phase lacks a control group and therefore does not allow for controlled inference regarding treatment efficacy.

The primary purpose of the extension phase was to assess longer-term outcomes, including durability of response, safety, and tolerability. The RCT phase provided the primary evidence for efficacy. We have clarified this distinction in the revised manuscript and now explicitly state that findings from the open-label phase should be interpreted descriptively. We have added to second paragraph in Discussion (p. 11).

“However, since this phase lacked a control group, these findings should be interpreted descriptively and do not permit causal inference regarding efficacy, but instead inform on longer-term safety and durability of response.”

Comment from reviewer: “baseline imbalance in concomitant medication could introduce confounding” and “the regression analysis identifying predictors of symptom change was exploratory in nature and should be interpreted accordingly”

Author response

We agree that baseline imbalances in concomitant medication could potentially introduce confounding and therefore examined this carefully.

In the revised manuscript, we explicitly addressed this issue by including concomitant medication use (including SNRI treatment) as a covariate in post-hoc analyses. In a stepwise linear regression model predicting change in SHAPS from baseline to week 9, treatment allocation remained the strongest predictor of outcome. SNRI use was not significantly associated with treatment response and did not alter the primary findings.

To address this comment, we now revised the discussion as follows (p. 11):

“Although SNRI use was imbalanced between groups, there is no evidence that SNRIs meaningfully affect pramipexole pharmacokinetics, and post-hoc analyses indicated no impact on the results; however, residual confounding cannot be fully excluded.”

We also clearly frame this as a post-hoc analysis both in Results and Discussion

Comments from reviewer: “sensitivity analyses relied on last observation carried forward methods” and “sensitivity analyses relied on last-observation-carried-forward imputation for missing data, and while the results were similar to the primary analysis, this approach represents only one possible strategy for handling missing outcomes.”

Author response

We agree that LOCF represents only one of several possible approaches for handling missing data. In this study, LOCF was included as a sensitivity analysis to assess the robustness of the findings under an alternative assumption regarding missingness, complementing the primary analyses based on mixed-effects models.

Importantly, the results were consistent across these approaches, supporting the robustness of the primary findings. We have clarified the role of the LOCF analysis in the revised manuscript and explicitly present it as a sensitivity analysis, with a dedicated subsection in the Results section.

Comments from reviewer:

Prior randomized placebo-controlled trials provide convergent evidence that pramipexole may have antidepressant efficacy in bipolar and treatment-resistant depression, but direct replication of the present anhedonia-focused trial appears limited.

Author response

We agree that there is emerging evidence supporting the antidepressant effects of pramipexole; however, to our knowledge, this is the first RCT specifically targeting patients with clinically significant anhedonia.

In bipolar depression, the most recent RCT (McAllister-Williams et al., 2025) was inconclusive due to limited statistical power (n = 39) and highlighted the need for further studies. In contrast, a recent large-scale RCT in treatment-resistant unipolar depression (Browning et al., 2025) demonstrated a robust antidepressant effect, including improvements in anhedonia (SHAPS).

To address this legitimate point, we have added the following text to the Discussion section (p. 11):

“Although prior studies support the antidepressant effects of pramipexole, our study is the first to specifically target patients with clinically significant anhedonia. Our findings extend this literature by focusing on a mechanistically defined subgroup characterized by prominent anhedonia”

Comments from reviewer:

C. Data & methodology: validity of approach, quality of data, quality of presentation
The revised manuscript provides more detailed experimental documentation, including demographic descriptions of participants who discontinued early and reporting of the open-label extension phase. It extends the scope by describing an open-label extension study and reporting how many participants enrolled and completed the six-month follow-up. It presents statistical results such as treatment effect estimates, p-values, effect sizes (Hedge's g), and correlations between neural activation and symptom improvement.
D. Appropriate use of statistics and treatment of uncertainties
The revised manuscript is concise and focused. It directly reports the study outcomes and includes numerical estimates such as effect sizes, p-values, and sample sizes. This makes it clear in communicating the key findings of the study. It interprets the study findings and concludes that pramipexole is an efficacious and safe add-on treatment for anhedonia in mood disorders. It links clinical improvement to changes in reward-related neural activity.

Author response
We thank the reviewer for their positive and thoughtful evaluation of the revised manuscript and are pleased that the additional analyses and clarifications have improved the transparency and clarity of the study.

E. Conclusions: robustness, validity, reliability
Several considerations should be noted when interpreting these findings.

Comment from reviewer:

Although response and remission rates were numerically higher in the pramipexole group, these differences were not statistically significant and therefore provide weaker support for treatment benefit than the continuous SHAPS analysis.

Author response

Thanks for this important comment. We agree that the absence of statistically significant differences in response and remission rates should be interpreted with caution.

The RCT was designed and powered based on a pre-specified continuous primary outcome (SHAPS), which we selected as the most sensitive measure of change in anhedonia. Dichotomized outcomes such as response and remission are generally less sensitive and reduce statistical power, particularly when applied to scales such as the SHAPS that are not primarily validated for dichotomous thresholds. Accordingly, these outcomes were included as secondary measures.

We have clarified this in the revised manuscript and now explicitly note that the non-significant findings for SHAPS response and remission likely reflect limited statistical power for these endpoints. Importantly, these results were directionally consistent with the primary outcome. We have added this to the Limitations (p. 14):

"The lack of statistically significant differences in dichotomized SHAPS remission and response outcomes may reflect reduced statistical power and the inherent limitations of dichotomizing continuous measures"

Comments from reviewer:
In addition, improvement observed during the open-label extension phase was reported descriptively and without a concurrent control group, which limits causal interpretation of changes during this period. The regression analysis identifying predictors of symptom change was exploratory in nature and should be interpreted accordingly. Finally, sensitivity analyses relied on last-observation-carried-forward imputation for missing data, and while the results were similar to the primary analysis, this approach represents only one possible strategy for handling missing outcomes.

Author response

These concerns have been addressed above

Comments from reviewer:

F. Clarity and context: lucidity of abstract/summary, appropriateness of abstract, introduction and conclusions
This revised manuscript primarily summarizes empirical findings from a randomized controlled trial evaluating the dopamine agonist pramipexole for the treatment of anhedonia in mood disorders. It provides the background and theoretical rationale motivating the study. The revised manuscript is concise and focused. It directly reports the study outcomes and includes numerical estimates such as effect sizes, p-values, and sample sizes. This makes it clear in communicating the key findings of the study. It contributes some empirical evidence derived from the clinical trial. It presents treatment effects, behavioral outcomes, neuroimaging findings, and safety information.
G. Overall Assessment
The revised manuscript presents the quantitative results of the study. It provides a broader and more detailed clinical narrative that includes screening context and long-term follow-up through an open-label extension study. The study established the conceptual and biological rationale for the investigation with a limited sample. The introduction defines the research question, while the results section provides the evidence addressing that question. Small sample concerns limit the findings of the results.

Author response

We thank the reviewer for their positive and thoughtful evaluation of the revised manuscript.

Reviewer #4 (Remarks to the Author):

The revised manuscript provides a more clear and direct picture of the study design, research findings, and offers appropriate interpretations. Thank you for seriously reviewing and addressing the prior recommendations.

I really like the addition of the tracking from 9-weeks up to 5 months.

Author response

We thank the reviewer for their thoughtful and positive feedback and are pleased that the revisions have improved the clarity of the manuscript. We also appreciate the reviewer's support for the inclusion of the longer-term follow-up data.

Comment from reviewer:

Here is a small remaining question related to the additional data:

* Figure 2. Longitudinal changes from baseline in Snaith–Hamilton Pleasure Scale (SHAPS) scores and
231 light physical activity (LPA) levels: Please cross-check the graph, there appears to be a conflict with the x-axis (e.g., Snaith–Hamilton Pleasure Scale (SHAPS) scores at week 9 can;t be prior to months 1 and 2). Is this just a labeling issue, or a problem with the result presentation?
Also, for LPA, bottom graph), same issue and apparently no difference between pramipexole (purple line) or placebo (grey line) between week 10 and month 5? Please clarify.

Author response

After unblinding, the x-axis time scale changes from weeks to months; month labels indicate time since unblinding, not time since RCT initiation. This clarification has been added to the figure legend, and a zigzag pattern has been included on the x-axis to indicate a discontinuous timeline.

For the LPA outcome, the apparent lack of difference between the pramipexole (purple) and placebo (grey; switching to purple after unblinding) groups from unblinding at week 9 through month 5, reflects the post-unblinding study design, as both groups received active treatment and between-group differences were no longer anticipated.

Importantly, LPA increased significantly from unblinding (week 9) to month 5 in participants who transitioned from placebo to pramipexole (Hedges' $g = 0.72$, $p = 0.045$) as reported in the manuscript. A comparable increase in LPA was observed earlier in the trial among participants randomized to pramipexole, from baseline to month 3 ($p < 0.001$), corresponding to a similar duration of active treatment exposure (approximately five months). Accordingly, the figure is intended to illustrate improvement in LPA under active treatment in both groups during the extended phase, whereas inference regarding placebo versus pramipexole is restricted to the randomized, double-blind phase of the trial.
